# Global Incidence and Mortality of Gastric Cancer, 1980-2018

**DOI:** 10.1001/jamanetworkopen.2021.18457

**Published:** 2021-07-26

**Authors:** Martin C. S. Wong, Junjie Huang, Paul S. F. Chan, Peter Choi, Xiang Qian Lao, Shannon Melissa Chan, Anthony Teoh, Peter Liang

**Affiliations:** 1 The Jockey Club School of Public Health and Primary Care, Faculty of Medicine, Chinese University of Hong Kong, Hong Kong; 2 Department of Global Health, School of Public Health, The Peking University, Beijing, China; 3 School of Population Medicine and Public Health, Chinese Academy of Medical Sciences and Peking Union Medical College, Beijing, China; 4 Department of Surgery, Faculty of Medicine, Chinese University of Hong Kong, Hong Kong; 5 Division of Gastroenterology, Department of Medicine, NYU Langone Health, New York, New York

## Abstract

**Importance:**

Gastric cancer is one of the most common cancers, with a high mortality-to-incidence ratio. It is uncertain whether developed nations may encounter an increasing burden of gastric cancer in young adults, as occurs for other cancers.

**Objectives:**

To evaluate the incidence and mortality of gastric cancer and compare the global incidence trends between younger (<40 years) and older (≥40 years) populations.

**Design, Setting, and Participants:**

This population-based cohort study analyzed data from global and national cancer registries, including data from 1980 to 2018, with at least 15 calendar years of incidence and mortality data. Data on age-standardized incidence and mortality rates of gastric cancer among 48 countries were retrieved from the Surveillance, Epidemiology, and End Results Program, the National Cancer Institute, the Nordic Cancer Registries, and the World Health Organization Mortality Database. The 10-year incidence trend of gastric cancer was assessed by age and sex. The 2018 GLOBOCAN database was used for reporting the global incidence and mortality of gastric cancer, the most recent data available at the time of analysis. Analyses were performed between January 10, 2020, and March 20, 2020.

**Main Outcomes and Measures:**

The average annual percent change (AAPC) of the incidence and mortality trends as evaluated by joinpoint regression analysis.

**Results:**

A total of 1 033 701 new cases of gastric cancer and 782 685 related deaths were reported in 2018. Overall, the incidence of gastric cancer decreased in 29 countries, and mortality decreased in 41 countries. The age-standardized incidence of gastric cancer decreased from a range of 2.6 to 59.1 in 1980 to a range of 2.5 to 56.8 in 2018 per 100 000 persons. The overall age-standardized mortality rate changed from a range of 1.3 to 25.8 in 1980 to a range of 1.5 to 18.5 in 2018 per 100 000 persons, but increasing mortality was observed in Thailand (female: AAPC, 5.30; 95% CI, 4.38-6.23; *P* < .001; male: AAPC, 3.92; 95% CI, 2.14-5.74; *P* < .001). The incidence of gastric cancer decreased in most regions among individuals 40 years or older and increased in populations younger than 40 years in several countries, including Sweden (male: AAPC, 13.92; 95% CI, 7.16-21.11; *P* = .001), Ecuador (female: AAPC, 6.05; 95% CI, 1.40-10.92; *P* = .02), and the UK (male: AAPC, 4.27; 95% CI, 0.15-8.55; *P* = .04; female: AAPC, 3.60; 95% CI, 3.59-3.61; *P* < .001).

**Conclusions and Relevance:**

In this population-based cohort study, an increasing incidence of gastric cancer was observed in younger individuals in some countries, highlighting the need for more preventive strategies in younger populations. Future research should explore the reasons for these epidemiologic trends.

## Key Points

**Question:**

Is the global burden of gastric cancer increasing in younger adults compared with older adults?

**Findings:**

In this cohort study of data from 1980 to 2018 covering more than 1 million cases of gastric cancer, an increasing incidence of gastric cancer was observed in individuals younger than 40 years in a significant number of countries, including Sweden, the UK, and Ecuador.

**Meaning:**

These findings suggest that the prevention of gastric cancer should become a priority in clinical guidelines and policy agendas to ameliorate its associated morbidity and mortality, especially among younger populations.

## Teaser

This cohort study uses data from national and global cancer registries to evaluate the incidence and mortality of gastric cancer between 1980 and 2018 and compares the global incidence between younger and older populations.

## Introduction

Worldwide, gastric cancer is one of the most common cancers, contributing to more than 1 million cases per year and 5.7% of all cancer diagnoses.^1,2^ Its prognosis is poor as evidenced by the 5-year survival rate and because most cases are already metastatic when diagnosed.^2^ The incidence of gastric cancer has wide geographic variation by up to 15- to 20-fold^3^ and is more commonly diagnosed in developed nations.^3^ High-risk regions include East Asia, Eastern Europe, and Central and South America.^4^ According to the International Agency for Research on Cancer, its incidence rate was significantly higher in countries with a high-middle Human Development Index (HDI) than in those with a low-middle HDI (20 vs 6.6 per 100 000 population).^5^

Although the incidence of gastric cancer has been decreasing in the past few decades,^3^ this decrease is much less marked in some populations, such as US White individuals and individuals in Canada, Colombia, Brazil, Denmark, Germany, India, and Israel.^4^ Recognized causes of stomach cancer include infection with *Helicobacter pylori* (which is the main risk factor)^6^; gastric ulcer disease^7^; gastroesophageal reflux disease^8,9^; obesity^2^; cigarette smoking^10^; chemical exposure to high-temperature particulate, asbestos, or metals^2,11^; consumption of high-salt foods^12^; *N*-methyl-*N*-nitro-*N*-nitrosoguanidine^13^; preserved or grain-fed meat^13^; coffee^14^; alcohol^15^; gastric surgery^16^; radiation exposure^17^; Epstein-Barr virus^18^; and inherited mutation of GSTM-1–null phenotype or *CDH1* (OMIM 192090) gene.^19^ Male sex, lower socioeconomic status, and certain races/ethnicities, such as Asian/Pacific Islander, American Indian/Alaska Native, and Hispanic, are associated with a higher risk of gastric cancer.^2^ Thus far, few reports^2,4^ have evaluated the global incidence and mortality of gastric cancer. Previous studies^4,20^ have not reported the cumulative risk of gastric cancer incidence and death by region and HDI. Temporal trends have been analyzed overall without age stratification, and changes in mortality rates have not been not examined.

In the past decades, there was a worldwide trend of rapid urbanization, industrialization, and adoption of a westernized diet. A previous global analysis^21^ of incidence and mortality rates of colorectal cancer in 39 countries found a significantly greater incidence increase among younger individuals (<50 years) when compared with populations older than 50 years. Because gastric cancer and colorectal cancer shared a certain proportion of risk factors, the former cancer might also have a similar trend. Studying the incidence of gastric cancer in young individuals is important because younger patients tend to have signet ring cell and poorly differentiated cancer with worse prognosis. Younger patients also tend to present late with more advanced disease because of a lower self-perceived risk of the cancer. The objectives of this study are to evaluate the global incidence, mortality, and temporal trend of gastric cancer and test the hypothesis that younger populations have a trend toward increasing incidence as compared with older individuals.

## Methods

### Data Source

We used 40 years of age as the cutoff for analysis because most previous literature^22,23^ defined early-onset gastric cancer using this age point. We retrieved the data on HDI for each country or city, a composite indicator of income per capita, period of education, and life expectancy from the United Nations.^24^ The GLOBOCAN database was used for the global incidence and mortality of gastric cancer in 2018^37^; we separately presented the incidence and mortality of gastric cancer in 2018 because they were the most recent and comprehensive data at the time of analysis.The HDI scores were defined as follows: low, less than 0.550; medium, 0.550 to 0.699; high, 0.700 to 0.799; and very high, 0.800 or greater.^24^ Deidentified data from 1980 to 2018 with at least 15 calendar years of incidence and mortality data were extracted from global or national databases (eTable 1 in the Supplement). The representative countries were selected if they had the following: (1) population-based registries that recorded cancer incidence and mortality for a specified period; (2) cancer registries by international rules to ensure comparability; (3) data that are comprehensive and available for subgroup by age and sex; and (4) validation from extensive previous publications that examined the global trends of cancer incidence and mortality. To obtain incidence data, we searched country-specific registries based on the *Cancer Incidence in Five Continents* (*CI5*), volumes I to XI.^25^ To retrieve the most updated incidence data, we used publicly available data from the Surveillance, Epidemiology, and End Results program, the National Cancer Institute,^25,26^ and the Nordic Cancer Registries (for European countries).^26,27,28,29^ Data contained in these registries are considered the standards for quality among cancer databases because they are comprehensive.^26,27,28,29,30^ We identified gastric cancer based on the *International Classification of Diseases, 10th Revision (ICD-10)* code C16. We used the World Health Organization Mortality Database to retrieve mortality figures,^31,32^ which were classified using *ICD-10* (code C16). Finally, a total of 48 countries were selected for the trend analysis. However, the incidence data were not available for Belgium, Latvia, Portugal, Russia, and Singapore. Among the countries, 26 reported national cancer registries, whereas 22 were represented by cancer registries from major cities. In addition, all data were adjusted for age and computed into age-standardized rates based on the world’s standard population.^33^ This study was approved by the Survey and Behavioral Research Ethics Committee, Chinese University of Hong Kong, and was determined not to involve human participants; informed consent was therefore waived.

The study was reported according to the recommendations from Global Cancer Observatory (GCO), International Agency for Research on Cancer, World Heath Organization.

### Statistical Analysis

The analyses were performed between January 10 and March 20, 2020. We evaluated the incidence and mortality trends in various nations based on average annual percent change (AAPC) by joinpoint regression analysis.^34^ The analysis was restricted to the data from registries that have been in operation throughout the entire study period. We performed a logarithmic transformation of the incidence and mortality data, and SEs were calculated based on binomial approximation.^35^ We used a maximum of 3 joinpoints as the option of analysis based on a previous study.^35^ We derived the AAPC as an average of APCs using geometric weighting in populations of different age stratum (<40 vs ≥40 years) and sex. Only the recent 10 years of incidence and mortality data were used to evaluate the time trend. Countries with missing or zero values in their decade data were excluded from the analysis because a joinpoint regression could not be conducted in this circumstance. Weights equivalent to each segment’s length were apportioned for the specified interval.^36^ The approximate 95% CI for AAPC was calculated by the empirical quantile method. The *P* value for a 2-sided test that the true AAPC is 0 is calculated based on a 2-tailed *t* test distribution. Because multiple tests could be performed, the significance level of each test was adjusted to control the overall type I error using the Bonferroni adjustment. A 2-sided *P* < .05 (number of tests in the regression) was considered to be statistically significant.^34^ The number of tests was determined by the number of joinpoints used. The joinpoint regression analysis was performed using Joinpoint Regression Program, version 4.8.0.1 (Surveillance Research Program, National Cancer Institute).

## Results

### Incidence and Mortality Rates of Gastric Cancer in 2018

**Figure 1.**
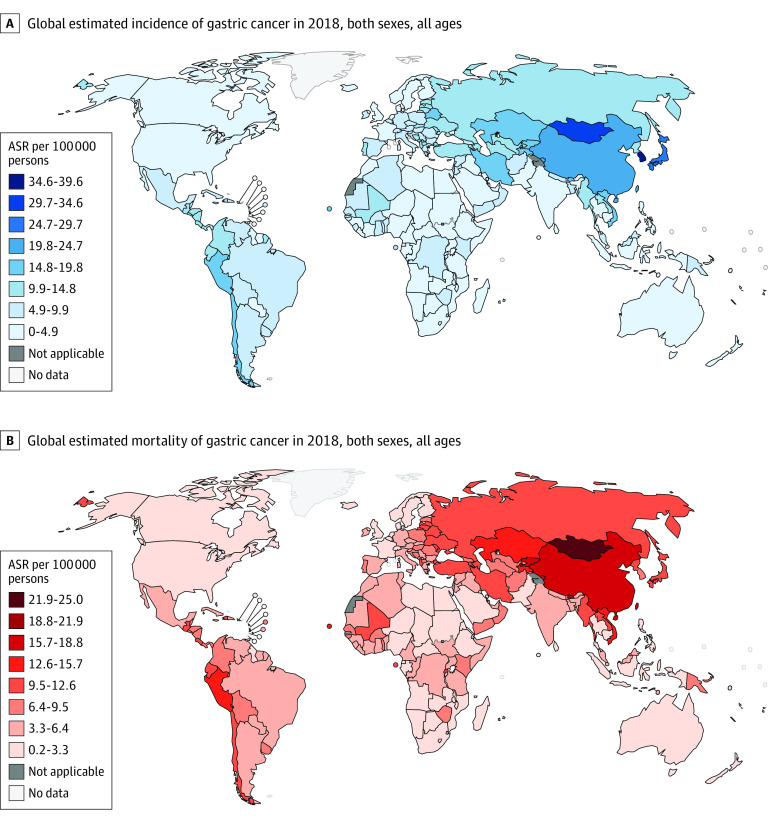
Global Incidence Rates of Gastric Cancer Data are from Bray et al.^1^ Reprinted with permission from Ferlay et al.^5^ ASR indicates age-standardized rate.

A total of 1 033 701 new cases of gastric cancer and 782 685 related deaths were reported in 2018 (Figure 1; eTable 2 in the Supplement).^37^ The cumulative risk of gastric cancer was the highest in Eastern Asia (2.64%) and lowest in southern Africa (0.42%) and highest in countries with high HDI (1.97%) and lowest in countries with low HDI (0.49%). The cumulative risk of gastric cancer death was the highest in Eastern Asia (1.84%) and lowest in Micronesia (0.21%) and highest in countries with high HDI (1.61%) and lowest in countries with low HDI (0.48%).

### Temporal Trends of Gastric Cancer

The incidence and mortality trends of each country are shown in eFigure 1 in the Supplement, and the corresponding findings from the joinpoint regression analysis are presented in eFigure 2 in the Supplement. Overall, the incidence of gastric cancer decreased in 29, countries and mortality decreased in 41 countries. The age-standardized incidence of gastric cancer decreased from a range of 2.6 to 59.1 to a range of 2.5 to 56.8 per 100 000 persons. The overall age-standardized mortality rate changed from a range of 1.3 to 25.8 in 1980 to a range of 1.5 to 18.5 in 2018 per 100 000 persons.

### Incidence of Gastric Cancer in Younger vs Older Individuals

**Figure 2.**
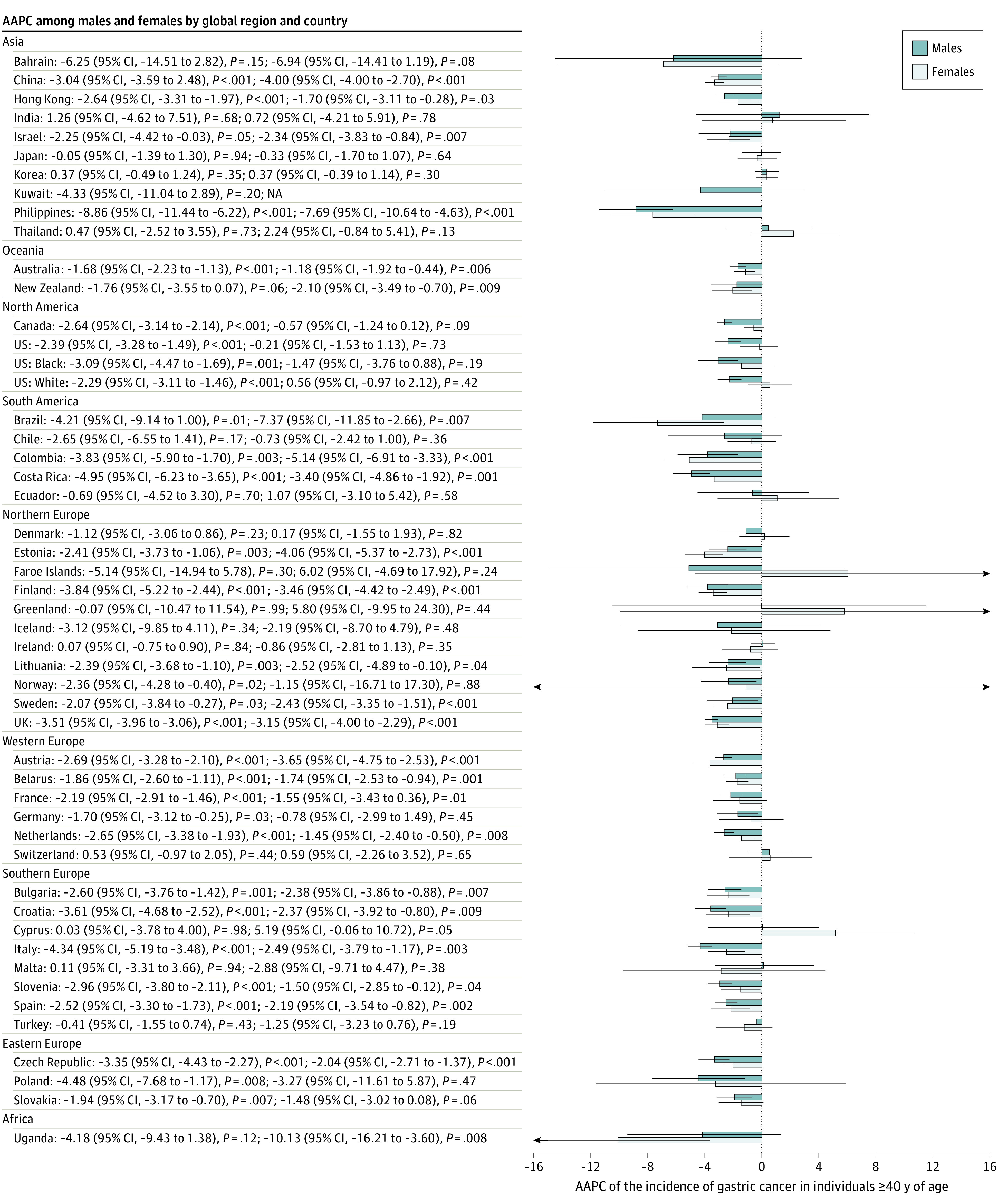
Average Annual Percent Change (AAPC) of the Incidence of Gastric Cancer in Individuals 40 Years or Older Error bars indicate 95% CIs. NA indicates not available.

**Figure 3.**
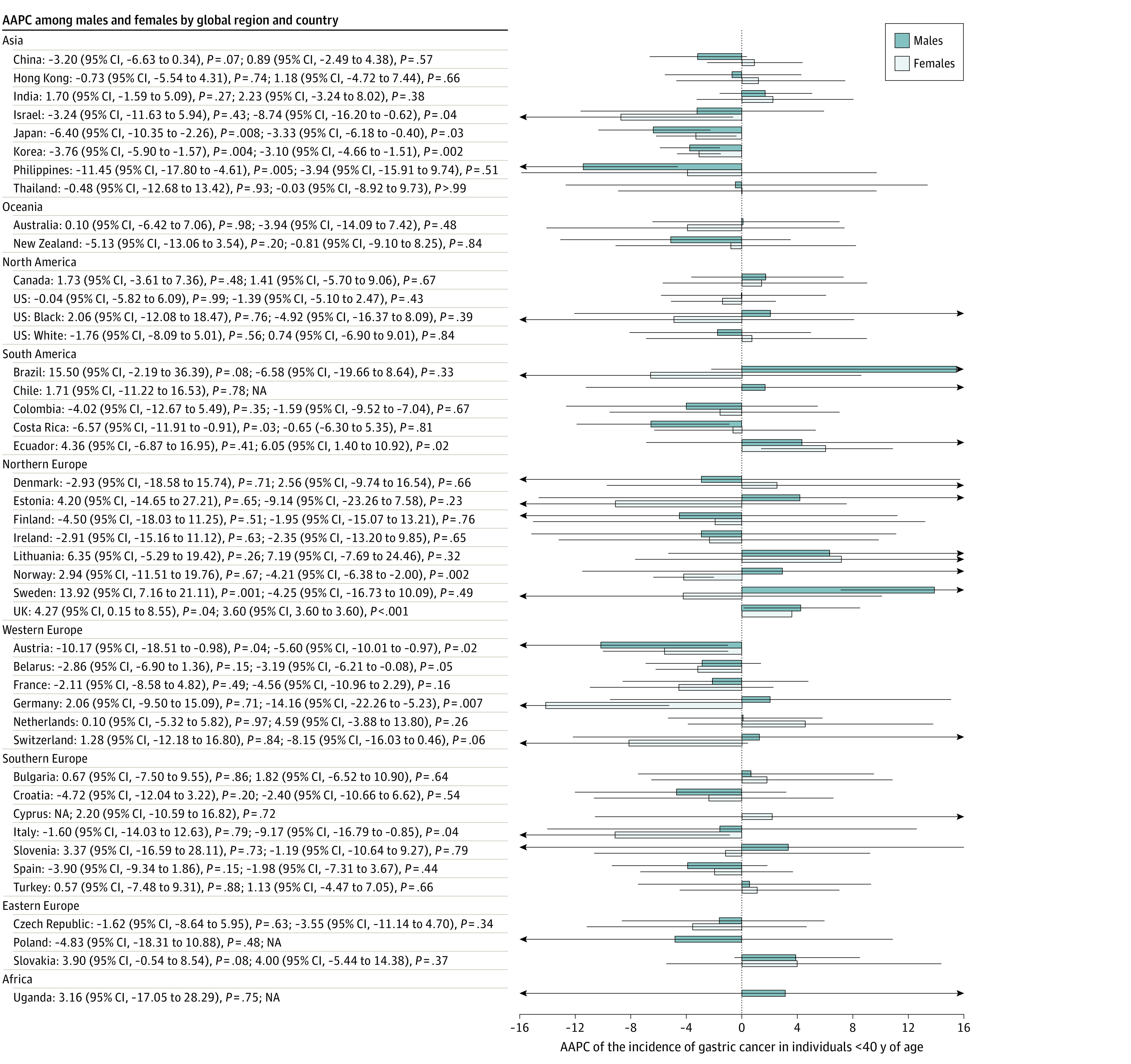
Average Annual Percent Change (AAPC) of the Incidence of Gastric Cancer in Individuals Younger Than 40 Years

The incidence of gastric cancer decreased in most countries (30 of 48) among individuals 40 years or older (Figure 2) and increased in populations younger than 40 years (Figure 3) in a number of countries, including Sweden (male: AAPC, 13.92; 95% CI, 7.16-21.11; *P* = .001), Ecuador (female: AAPC, 6.05; 95% CI, 1.40-10.92; *P* = .02), and the UK (male: AAPC, 4.27; 95% CI, 0.15-8.55; *P* = .04; female: AAPC, 3.60; 95% CI, 3.59-3.61; *P* < .001).

### Incidence Trend by Sex

**Figure 4.**
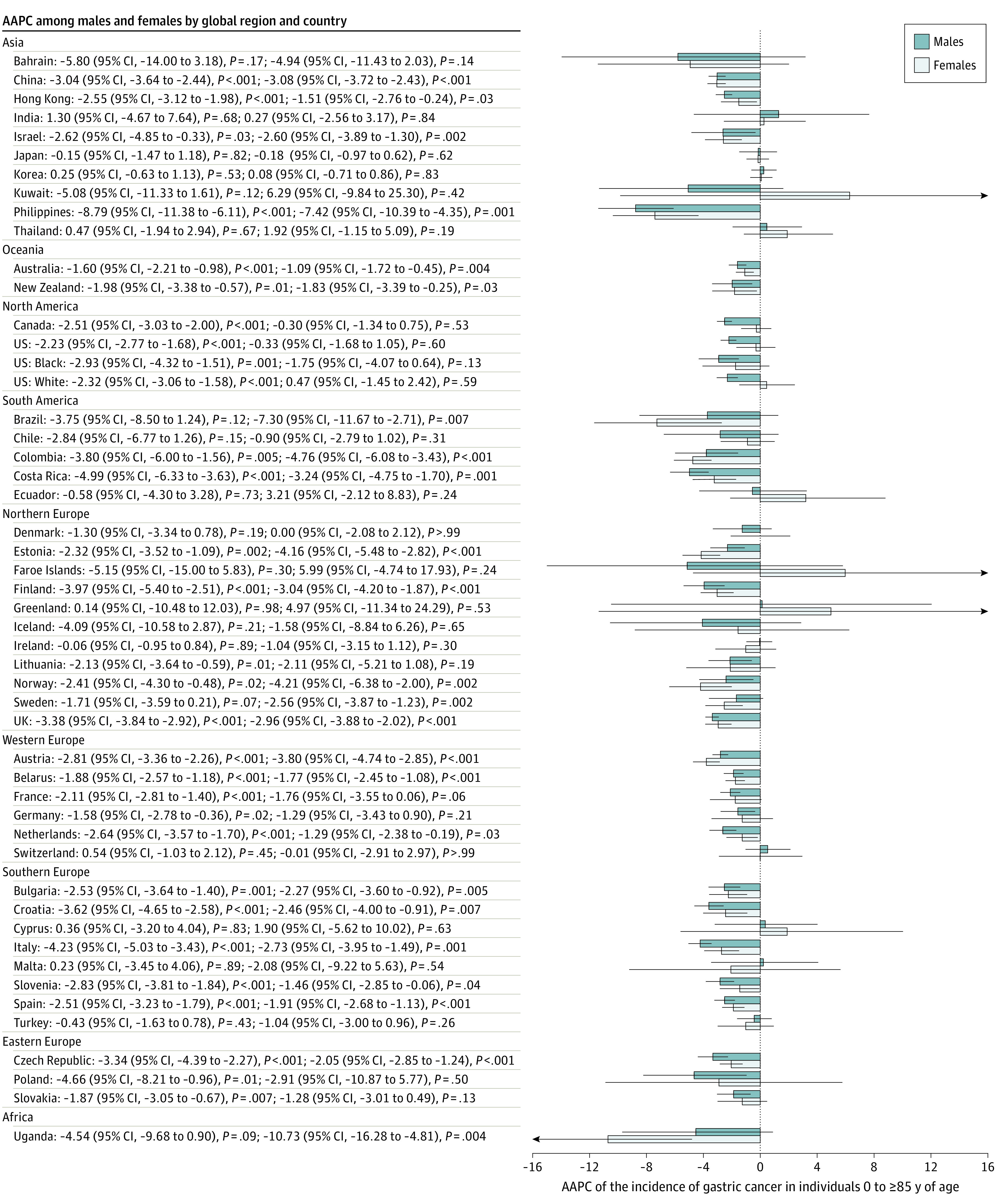
Average Annual Percent Change (AAPC) of the Incidence of Gastric Cancer in Individuals 0 to 85 Years or Older Error bars indicate 95% CIs.

Among men, 29 countries had a decrease in incidence, and 19 countries reported stable trends (Figure 4). None of the 48 countries had an increasing trend. Of all 29 countries with reduction in incidence, 18 were reported in Europe, including Poland (AAPC, −4.66; 95% CI, −8.21 to −0.96; *P* = .01), Italy (AAPC, −4.23; 95% CI, −5.03 to −3.43; *P* < .001), the UK (AAPC, −3.38; 95% CI, −3.84 to −2.92; *P* < .001), and Finland (AAPC, −3.97; 95% CI, −5.40 to −2.51; *P* < .001). Among women, 23 countries had a reduction in incidence, and 25 countries reported stable trends. Most of the decreases in incidence occurred in Northern Europe and Southern Europe. Uganda (AAPC, −10.73; 95% CI, −16.28 to −4.81; *P* = .004), the Philippines (AAPC, −7.42; 95% CI, −10.39 to −4.35; *P* = .001), and Brazil (AAPC, −7.30; 95% CI, −11.67 to −2.71; *P* = .007) had the largest incidence decreases.

### Mortality Trend by Sex

**Figure 5.**
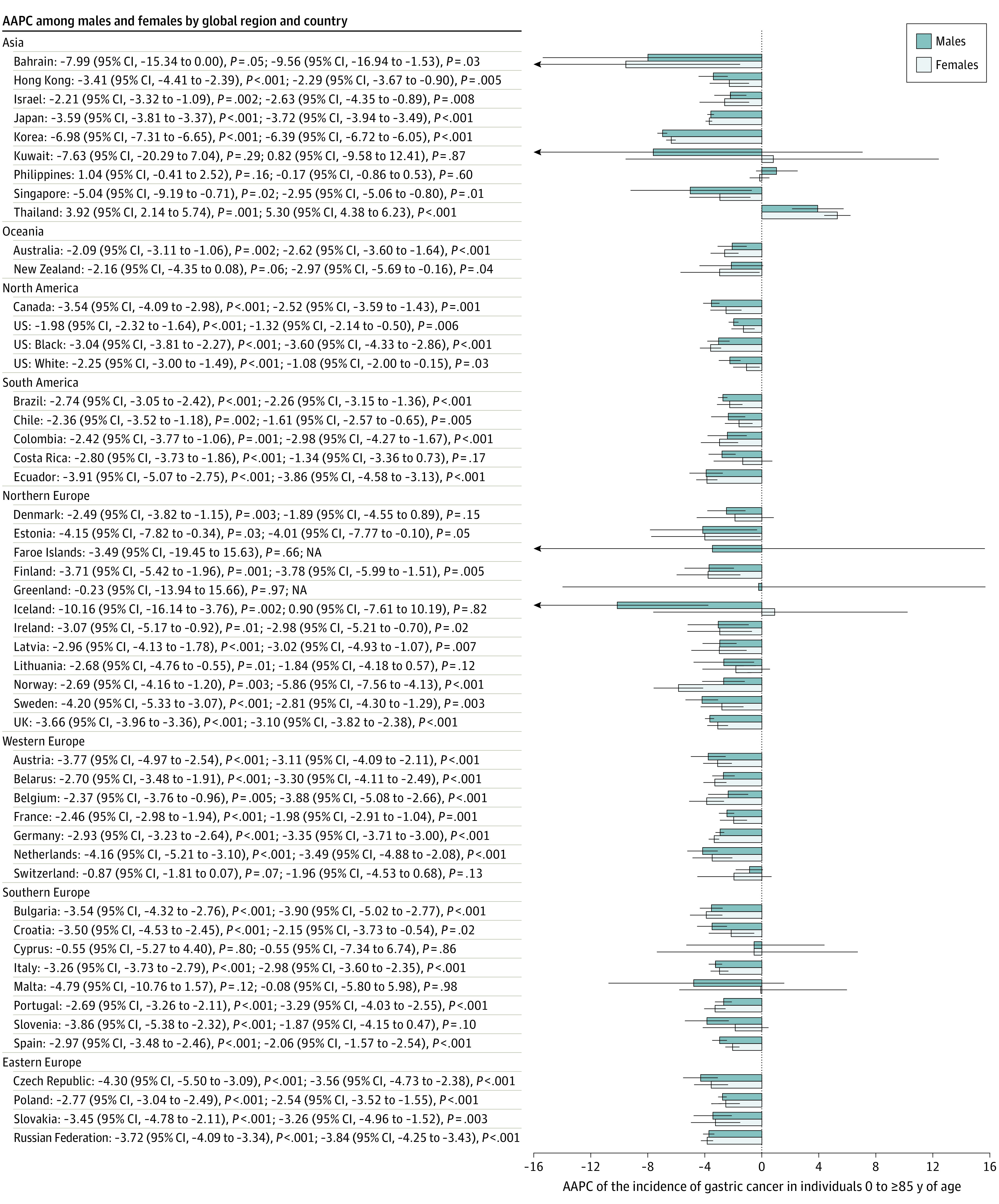
Average Annual Percent Change (AAPC) of the Mortality of Gastric Cancer in Individuals 0 to 85 Years or Older Error bars indicate 95% CIs. NA indicates not available.

In men, the only country that had a trend toward increasing mortality was Thailand (AAPC, 3.92; 95% CI, 2.14-5.74; *P* = .001) (Figure 5). A total of 41 of 48 countries reported decreasing mortality rates, and 25 of these decreases occurred in European countries. Among them, the Czech Republic (AAPC, −4.30; 95% CI, −5.50 to −3.09; *P* < .001), the Netherlands (AAPC, −4.16; 95% CI, −5.21 to −3.10; *P* < .001), and Estonia (AAPC, −4.15; 95% CI, −7.82 to −0.34; *P* = .03) had the most marked reduction. In women, Thailand (AAPC, 5.30; 95% CI, 4.38 to 6.23; *P* < .001) was the only country where an increase in mortality rates was observed. Among all 49 countries, 39 countries reported decreasing mortality rates, whereas 9 countries had stable trends. A total of 23 European countries reported a decrease in mortality trends. Countries that had the largest mortality decreases included Norway (AAPC, −5.86; 95% CI, −7.56 to −4.13; *P* < .001), Estonia (AAPC, −4.01; 95% CI, −7.77 to −0.10; *P* = .05), Ecuador (AAPC, −3.86; 95% CI, −4.58 to −3.13; *P* < .001), and Finland (AAPC, −3.78; 95% CI, −5.99 to −1.51; *P* = .005).

## Discussion

Several major findings were derived from this cohort study. First, the highest gastric cancer incidence and mortality were found in Eastern Asia. In addition, most countries reported a decreasing trend in gastric cancer incidence and mortality in the past decade, especially for male patients and those 40 years or older. However, an increasing incidence of gastric cancer was observed in individuals younger than 40 years in some countries, including Sweden, Ecuador, and the UK.

There was a substantial variation in the epidemiologic trends of gastric cancer across the regions. This study found that the highest incidence tended to predominate in Eastern Asia, which is consistent with findings from previous studies.^4,38^ The report from 2018 GLOBOCAN found that the incidence of gastric cancer was markedly increased in Eastern Asia, whereas the incidence in Northern America and Northern Europe was lower and comparable to that observed in Africa.^1^ This finding can partly be explained by the varied distribution of gastric cancer–related risk factors.^4^ *H pylori* has been classified as a class I carcinogen by the World Health Organization and is the most important risk factor for gastric cancer.^39^ It was estimated that nearly 90% of the noncardia gastric cancer cases (75% of all gastric cancers) were attributable to *H pylori* infection.^40^ Regions with a high incidence of gastric cancer were prone to high *H pylori* seroprevalence rates. Among the countries in Eastern Asia, the overall seroprevalence rate of *H pylori* was as high as 59.6% in South Korea^41^ followed by 58.1% in China^42^ and 39.3% in Japan^43^ between 2000 and 2010. However, for some regions, such as South Asia and Africa, the seroprevalence rate of *H pylori* was high, but the gastric cancer incidences are relatively low.^4^ This paradox—the lower burden of gastric cancer in the context of high prevalence of *H pylori*—is variably referred to as the Asian or African enigma.^44^ The reasons behind this phenomenon remain unclear and may reflect the complex interactions among host, bacterial, and environmental factors, such as poor sanitation.^45^ Dietary (high-salt diet with few vegetables) and lifestyle factors (smoking, coffee, and alcohol consumption) account for 33% to 50% of all gastric cancers.^46^ A previous study^12^ found that dietary intake of high-salt foods increased the risk of developing gastric cancer. Some researchers also observed an association between the availability of refrigerators and the risk of gastric cancer.^47^ Smoking increases the risk of gastric cancer by 50% to 60%,^48^ whereas the findings on alcohol remain inconsistent for gastric cancer.^15^ The risk of gastric cancer among individuals with obesity was increased by 22%.^49^ Other less common risk factors included chemical exposure to high-temperature particulate, asbestos, or metals^2,11^; *N*-methyl-*N*-nitro-*N*-nitrosoguanidine^13^; radiation exposure^17^; and Epstein-Barr virus.^18^

Although there were disparities in the disease burden of gastric cancer globally, we observed an overall decreasing trend of its incidence and mortality in the past decade. The substantial change in the incidence of gastric cancer may indicate the important roles of *H pylori* infection, lifestyle, and other environmental factors rather than genetics.^50^ The decreasing incidence may be attributable to the decrease in *H pylori* to a great extent. A study^45^ found that the decrease in *H pylori* infection paralleled a decrease in the incidence of gastric cancer in Asia. In European countries, a similar association was also observed in which there was a continuous decrease in gastric cancer, reflecting a steadily decreasing risk of *H pylori* in the population, probably because of improvement of sanitation and medical therapies.^51^ In addition, the decrease in smoking prevalence in most regions and populations (especially among male patients) may account for the incidence decrease. According to a recent report by the World Health Organization,^52^ the prevalence of current smoking decreased from 33.5% in 2000 to 29.3% in 2015 and was estimated to be 27.7% in 2025 if current interventions in tobacco control remained. The decreasing trend of gastric cancer may also be associated with reduction in salt consumption observed in some Asian countries. A study^53^ among 12 provinces in China found that salt intake decreased by 22.2% from 2000 to 2010 among the population. A similar trend was also found in Japan, although the salt intake was still high compared with that in European countries.^54^ Other factors contributing to the decreasing trend may include continuous improvements in food preservation technology and the supply of fresh fruits and vegetables.^55^ The favorable trend in mortality could be attributed to early cancer detection and surgical and oncologic advances. Despite the favorable trend, the decreases are less evident for some regions and populations, especially for the younger population. Despite the overall decreasing trend of gastric cancer, a study^56^ from Sweden found that the incidence of cardia gastric cancer had been increasing rapidly, especially in younger women. The main risk factors for cardia gastric cancer include obesity and gastroesophageal reflux disease, whereas the main risk factors for noncardia gastric cancer include *H pylori* infection and high intake of salty and smoked food.^57^

The reason for the recent increasing incidence of early-onset gastric cancer among the younger population remains unclear.^58^ Because the prevalences of *H pylori* infection, smoking, and alcohol drinking have been decreasing among the younger population, the increasing trend of early-onset gastric cancer may be attributable to other factors. One possible explanation could be the increasing trend of obesity and central obesity among the young population. A substantial body of evidence has confirmed the association between excess body weight and the risk of gastric cancer.^59^ It has been postulated that excess body weight may lead to cancer via insulin resistance, adipokine aberrations, subclinical low-grade inflammation with oxidative stress, altered gut microbiomes, microenvironmental perturbations, and circadian rhythm disruption.^59^ A meta-analysis^49^ found that excess body weight was associated with an increased risk of cardia gastric cancer but not of noncardia gastric cancer. Evidence from the World Health Organization indicates that childhood obesity has also increased more than 4.5-fold from 1975 (4%) to 2016 (18%).^60^ This obesity could be partially driving the increase in incidence among the younger population. A previous study^61^ found a larger increase in individuals 15 to 40 years of age (16.3% to 33.9%) compared with those older than 40 years (43.6% to 57.9%). Therefore, according to emerging evidence,^62^ cancer may have shifted to the younger population. Increasing coffee consumption in the younger population might also be contributory; a study^63^ indicated that high coffee consumption increased the risk of gastric cancer by 36%. Despite a slight decrease of 2% in overall coffee consumption in the population between 2008 and 2016, the consumption tripled among those 18 to 24 years of age from 13% to 36% and more than doubled among those 25 to 39 years of age from 19% to 41%, according to a US national report.^64^ This increasing incidence of gastric cancer in the younger population may also be associated with immigration from high-risk regions to low-risk regions and more invasive diagnostic procedures (endoscopy) among the younger population.^65^

More intensive, evidence-based prevention strategies should be implemented and become a top priority in clinical guidelines and policy agendas to ameliorate the morbidity and mortality of gastric cancer, especially among the younger population. These findings call for a targeted approach for early identification of and intervention for individuals at high risk to reduce the impact on their well-being, that of their families, and societal productivity. For instance, early diagnosis of gastric cancer may be achievable through endoscopic screening among the high-risk population. However, these screening programs should be implemented together with the eradication of *H pylori,* especially among the younger population.

### Limitations

This study has some limitations. First, underreporting of gastric cancer was more likely to occur in developing countries because of a less sophisticated reporting infrastructure of registries. Second, the incidences could have been overestimated in countries with higher HDIs because some regions were represented by estimates from their major cities. Third, the cancer registry system may vary across different countries and can change over time. Fourth, the analysis of incidence and mortality by age group for different subtypes of gastric cancer was not available. Fifth, the current analysis cannot include detailed information on the staging of gastric cancer; therefore, the association between pathogenesis and the timing of different gastric cancers could not be analyzed. Sixth, the results of trend analysis for some countries may not be available because of missing values.

## Conclusions

A decrease in incidence and mortality of gastric cancer was observed in this study. This may be attributable to the implementation of early prevention strategies and the improvement of effective therapeutic interventions. Public health policies should monitor risk factors, such as *H pylori* infection, smoking, alcohol drinking, and poor dietary habits, to minimize the effect on the health care system. For the younger population, although some of these factors are likely nonmodifiable, preventive measures that involve increased physical activity, which confers a benefit beyond that of reducing caloric expenditure; promotion of healthy dietary habits; and raising awareness among the general public are warranted. Future studies are warranted to investigate the reasons behind the increased incidence of gastric cancer in the younger population.

## Notes

Supplement. **eTable 1.** Data Source for the Age-Standardized Incidence/Mortality Rates **eTable 2.** The Incidence/Mortality of Gastric Cancer Worldwide and by Region **eFigure 1.** The Plots of Incidence and Mortality Trends for Each Country **eFigure 2.** The Graphs of the Joinpoint Regression Output
