# Supplementary material for: Global Incidence and Mortality of Gastric Cancer, 1980-2018
Source: JAMA Netw Open. 2021 Jul 26;4(7):e2118457. doi: 10.1001/jamanetworkopen.2021.18457 (PMC8314143; doi:10.1001/jamanetworkopen.2021.18457)
Supplement: Supplement. — eTable 1. Data Source for the Age-Standardized Incidence/Mortality Rates eTable 2. The Incidence/Mortality of Gastric Cancer Worldwide and by Region eFigure 1. The Plots of Incidence and Mortality Trends for Each Country eFigure 2. The Graphs of the Joinpoint Regression Output [file jamanetwopen-e2118457-s001.pdf]

Supplementary Online Content

Wong MCS, Huang J, Chan PSF, et al. Global incidence and mortality from gastric cancer, 1980-2018. *JAMA Netw Open*. 2021;4(7):e2118457. doi:10.1001/jamanetworkopen.2021.18457

eTable 1. Data Source for the Age-Standardized Incidence/Mortality Rates

eTable 2. The Incidence/Mortality of Gastric Cancer Worldwide and by Region

eFigure 1. The Plots of Incidence and Mortality Trends for Each Country

eFigure 2. The Graphs of the Joinpoint Regression Output

This supplementary material has been provided by the authors to give readers additional information about their work.

© 2021 Wong MCS et al. *JAMA Network Open.*

eTable 1: Data source for the age-standardized incidence and mortality rates of gastric cancer

	Incidence	Mortality
Australia	CI5 (1993-2012)	WHO (1979-2015)
Austria	CI5 (1998-2012)	WHO (1980-2016)
Bahrain	CI5 (1998-2012)	WHO (1985-2014)
Belarus	CI5 (1983-2012)	WHO (1981-2014)
Belgium	n/a	WHO(1979-2015)
Brazil	CI5 (1993-2012)[1]	WHO (1979-2015)
Bulgaria	CI5 (1998-2012)	WHO (1980-2014)
Canada	CI5 (1983-2012)[2]	WHO (1979-2013)
Chile	CI5 (1998-2012)[3]	WHO (1980-2015)
China	CI5 (1998-2012)[4]	n/a
Colombia	CI5 (1983-2012)[5]	WHO (1984-2015)
Costa Rica	CI5 (1982-2011)	WHO (1980-2014)
Croatia	CI5 (1988-2012)	WHO (1985-2016)
Cyprus	CI5 (1998-2012)	WHO (1999-2016)
Czech Republic	CI5 (1983-2012)	WHO (1986-2016)
Denmark	NORDCAN (1943-2016)	NORDCAN (1951-2016)
Ecuador	CI5 (1985-2012)[6]	WHO (1979-2015)
Estonia	CI5 (1983-2012)	WHO (1981-2015)
Faroe Islands	NORDCAN (1983-2013)	NORDCAN (1960-2015)
Finland	NORDCAN (1953-2016)	NORDCAN (1953-2016)
France	CI5 (1998-2012)[7]	WHO (1979-2014)
Germany	CI5 (1998-2012)[8]	WHO (1990-2015)
Greenland	NORDCAN (1968-2016)	NORDCAN (1983-2016)
Hong Kong, SAR, China	CI5 (1983-2012)	WHO (1979-2015)
Iceland	NORDCAN (1951-2016)	NORDCAN (1955-2016)
India	CI5(1983-2012)[9]	n/a
Ireland	CI5 (1994-2012)	WHO (1979-2014)
Israel	CI5 (1963-2012)	WHO (1979-2015)
Italy	CI5 (1998-2010) [10]	WHO (1979-2015)
Japan	CI5 (1998-2010)[11]	WHO (1979-2015)

© 2021 Wong MCS et al. *JAMA Network Open.*

Korea	CI5 (1999-2012)[12]	WHO (1985-2016)[13]
Kuwait	CI5 (1998-2012)	WHO (1979-2014)
Latvia	n/a	WHO (1980-2015)
Lithuania	CI5 (1988-2012)	WHO (1981-2016)
Malta	CI5 (1993-2012)	WHO(1979-2015)
Netherlands	CI5 (1989-2012)	WHO (1979-2016)
New Zealand	CI5 (1983-2012)	WHO (1979-2013)
Norway	NORDCAN (1953-2012)	NORDCAN (1953-2016)
Philippines	CI5 (1983-2012)[14]	WHO (1992-2011)
Poland	CI5 (1998-2012)[15]	WHO (1980-2015)
Portugal	n/a	WHO (1980-2014)
Russian Federation	n/a	WHO (1980-2015)
Singapore	n/a	WHO (1979-2015)
Slovakia	CI5 (1971-2010)	WHO (1992-2014)
Slovenia	CI5 (1983-2012)	WHO (1985-2015)
Spain	CI5 (1993-2010)[16]	WHO (1980-2015)
Sweden	NORDCAN (1960-2016)	NORDCAN (1952-2016)
Switzerland	CI5 (1998-2012)[17]	WHO (1995-2015)
Thailand	CI5 (1993-2012)[18]	WHO (1979-2016)
Turkey	CI5 (1998-2012)[19]	WHO (2009-2015)
Uganda	CI5 (1993-2012)[20]	n/a
United Kingdom	CI5 (1995-2012)[21]	WHO (1979-2015)
USA	SEER(1975-2016) [22]	SEER(1968-2017) [22]
USA Black	SEER(1975-2016) [22]	SEER(1968-2017) [22]
USA White	SEER(1975-2016) [22]	SEER(1968-2017) [22]

n/a" not available; CI5: Cancer Incidence in Five Continents V; NORDCAN: Nordic Cancer Registries' SEER: USA: National Institutes of Health (NIH); WHO: World Health Organization

1. Brazil, Goiania
2. Canada (excl. Nunavut, Quebec and Yukon)
3. Chile, Valdivia
4. China (5 registries)
5. Colombia, Cali

© 2021 Wong MCS et al. *JAMA Network Open.*

6. Ecuador, Quito
7. France (9 registries)
8. Germany (2 registries)
9. India, Chennai
10. Italy (8 registries)
11. Japan (4 registries)
12. Korea (5 registries)
13. Republic of Korea
14. Philippines, Manila
15. Poland, Kielce
16. Spain (9 registries)
17. Switzerland (6 registries)
18. Thailand (4 registries)
19. Turkey (2 registries)
20. Uganda, Kampala
21. UK, England
22. USA, (9 registries)

Reference:

a. CI5:http://ci5.iarc.fr/CI5plus/Pages/table1_sel.aspx

b. NORDCAN:http://www-dep.iarc.fr/NORDCAN/english/frame.asp

c. SEER: http://seer.cancer.gov/data/seerstat/

d. WHO: http://apps.who.int/healthinfo/statistics/mortality/whodpms/

© 2021 Wong MCS et al. *JAMA Network Open.*

eTable 2: The incidence of mortality of gastric cancer worldwide and by region

	Incidence						Mortality					
	Both sexes		Males		Females		Both sexes		Males		Females	
	New cases	Cum. risk 0-74 (%)	New cases	Cum. risk 0-74 (%)	New cases	Cum. risk 0-74 (%)	Deaths	Cum. risk 0-74 (%)	Deaths	Cum. risk 0-74 (%)	Deaths	Cum. risk 0-74 (%)
Eastern Africa	9,215	0.5	4,572	0.53	4,643	0.47	8,908	0.49	4,404	0.53	4,504	0.47
Middle Africa	4,143	0.51	2,213	0.6	1,930	0.44	3,765	0.5	1,996	0.58	1,769	0.44
Northern Africa	7,702	0.45	4,518	0.56	3,184	0.34	6,727	0.38	4,066	0.48	2,661	0.28
Southern Africa	2,008	0.42	1,176	0.6	832	0.29	1,636	0.34	965	0.48	671	0.23
Western Africa	8,080	0.48	4,546	0.56	3,534	0.41	7,671	0.46	4,214	0.53	3,457	0.4
Caribbean	4,125	0.75	2,406	0.98	1,719	0.54	3,203	0.53	1,907	0.72	1,296	0.36
Central America	12,881	0.81	6,756	0.93	6,125	0.7	10,439	0.63	5,544	0.73	4,895	0.53
South America	50,052	1.06	29,720	1.46	20,332	0.73	38,272	0.78	23,323	1.1	14,949	0.51
North America	29,275	0.48	18,488	0.65	10,787	0.31	13,403	0.19	8,021	0.26	5,382	0.13
Eastern Asia	619,226	2.64	428,298	3.79	190,928	1.5	453,513	1.84	311,227	2.65	142,286	1.04
South-Eastern Asia	38,028	0.64	23,513	0.87	14,515	0.45	32,033	0.53	19,572	0.71	12,461	0.37
South-Central Asia	92,819	0.62	61,536	0.84	31,283	0.4	81,837	0.55	54,422	0.75	27,415	0.35
Western Asia	19,655	0.98	12,230	1.31	7,425	0.68	16,992	0.84	10,725	1.15	6,267	0.56
Central and Eastern Europe	64,482	1.41	38,427	2.13	26,055	0.89	53,268	1.1	31,836	1.72	21,432	0.67
Western Europe	27,596	0.67	17,795	0.93	9,801	0.41	18,425	0.36	11,421	0.5	7,004	0.22
Southern Europe	29,811	0.87	18,276	1.22	11,535	0.55	22,460	0.55	13,629	0.79	8,831	0.34
Northern Europe	11,244	0.51	7,113	0.68	4,131	0.34	8,014	0.31	4,994	0.43	3,020	0.2
Australia and New Zealand	2,702	0.53	1,796	0.74	906	0.32	1,517	0.26	959	0.35	558	0.17
Melanesia	583	0.89	327	1.17	256	0.66	532	0.84	293	1.1	239	0.62
Polynesia	43	0.83	28	1.07	15	0.61	53	0.86	28	0.92	25	0.8
Micronesia	31	0.69	20	0.93	11	0.47	17	0.21	9	0.1	8	0.33
Low HDI	22,717	0.49	12,486	0.57	10,231	0.41	21,568	0.48	11,688	0.55	9,880	0.41
Medium HDI	125,631	0.6	80,479	0.81	45,152	0.4	110,362	0.52	70,872	0.71	39,490	0.35
High HDI	568,166	1.97	386,728	2.82	181,438	1.16	479,659	1.61	325,453	2.32	154,206	0.94
Very high HDI	316,953	1.23	203,918	1.78	113,035	0.73	170,920	0.55	105,437	0.79	65,483	0.33
World	1,033,701	1.31	683,754	1.87	349,947	0.79	782,685	0.95	513,555	1.36	269,130	0.57

© 2021 Wong MCS et al. *JAMA Network Open.*

Data source: GLOBOCAN 2018 Graph production: IARC (http://gco.iarc.fr/today) World Health Organization

© 2021 Wong MCS et al. *JAMA Network Open.*

eFigure 1: The plots of incidence and mortality trends for each country

male

Asia

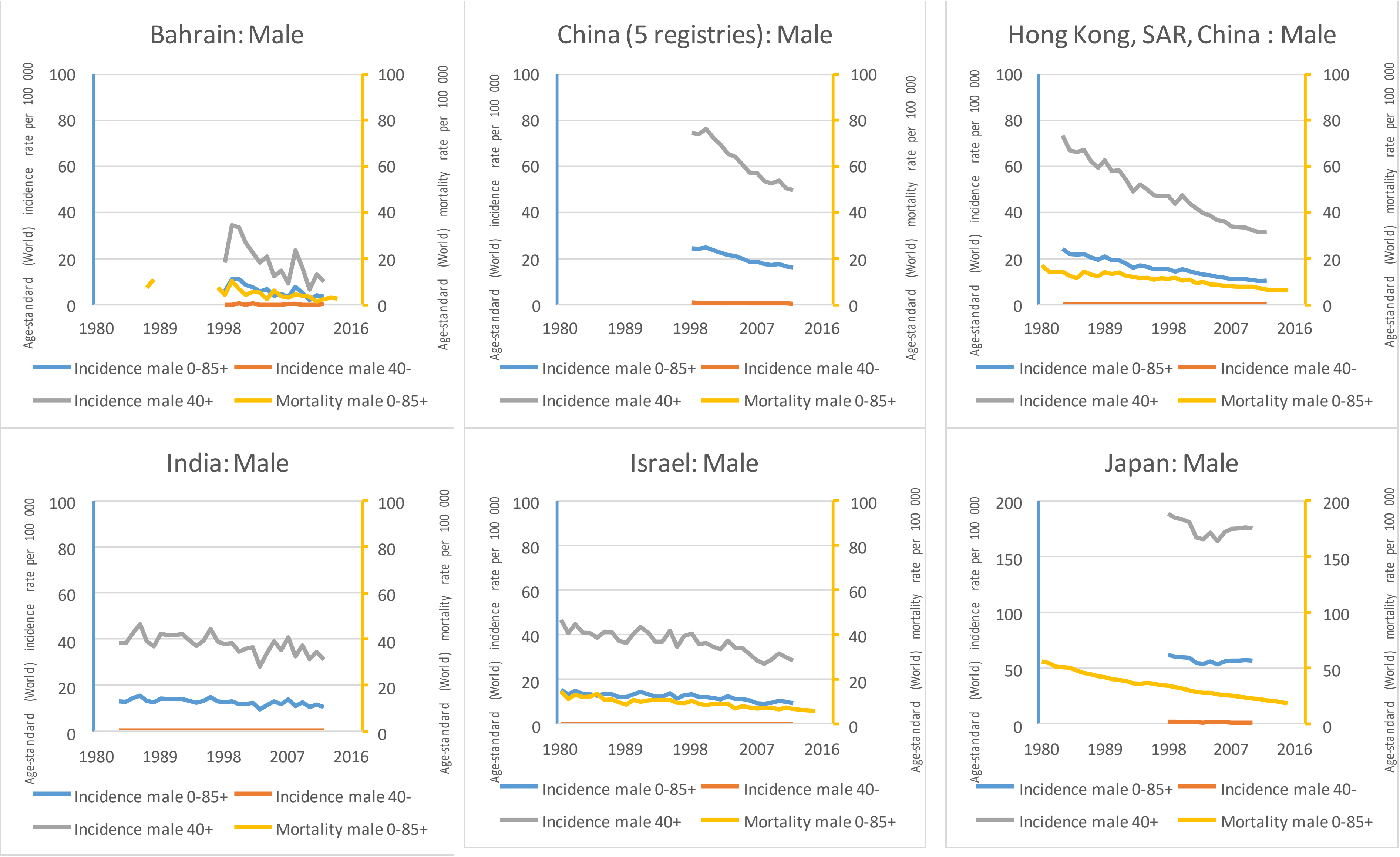

© 2021 Wong MCS et al. *JAMA Network Open.*

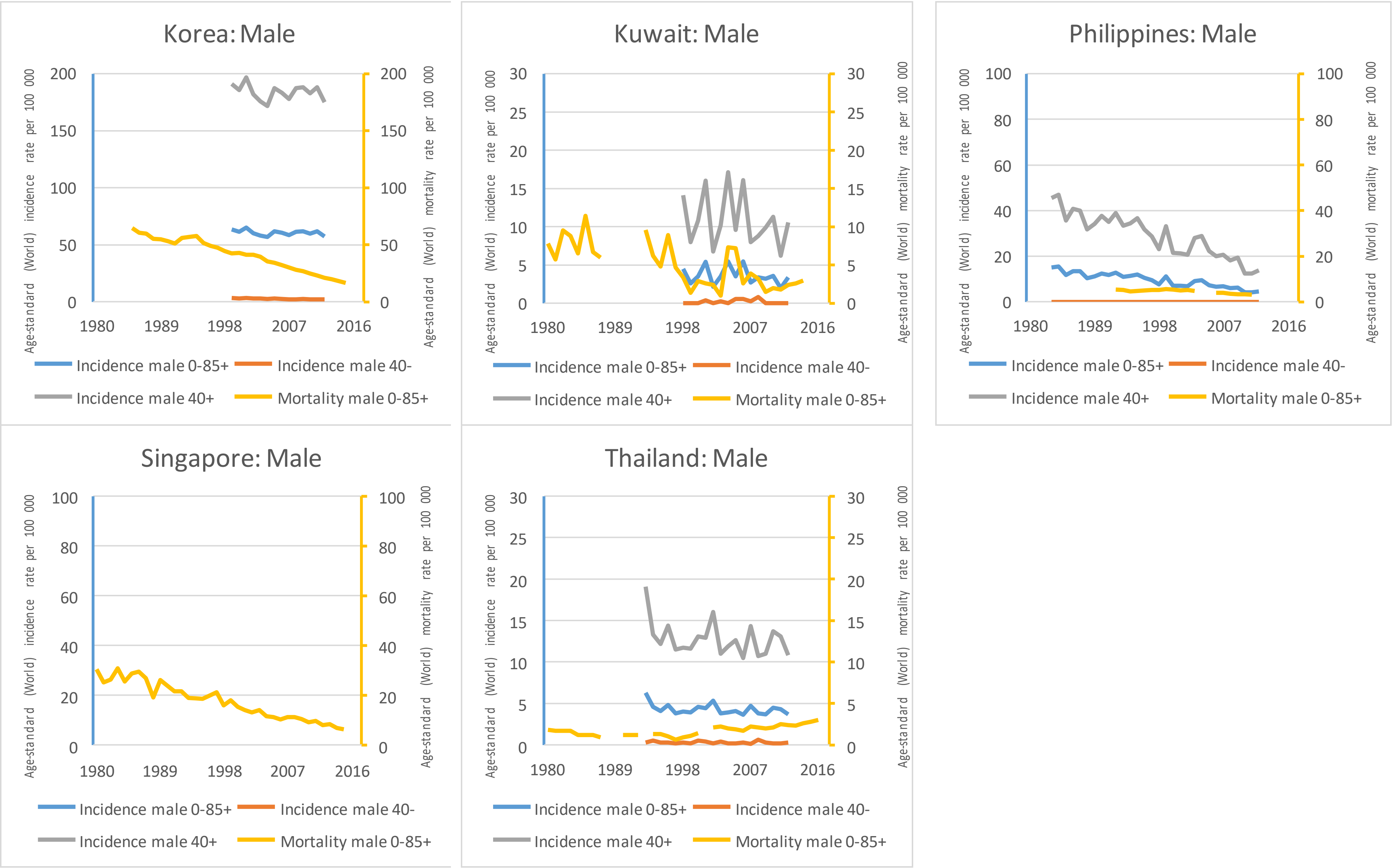

© 2021 Wong MCS et al. *JAMA Network Open.*

Oceania

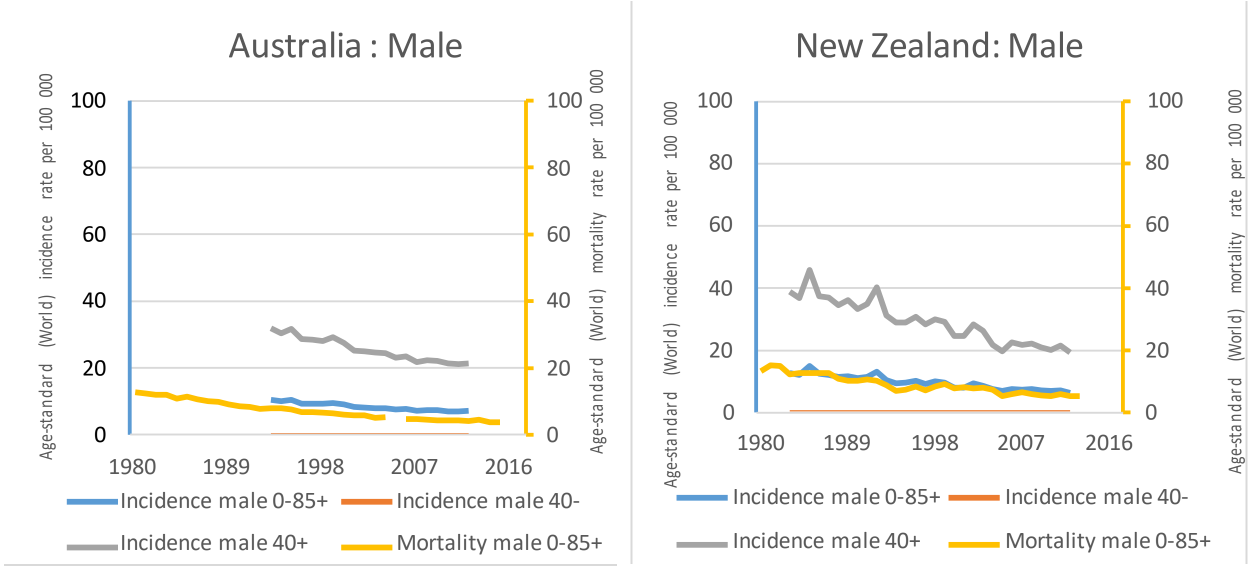

Northern America

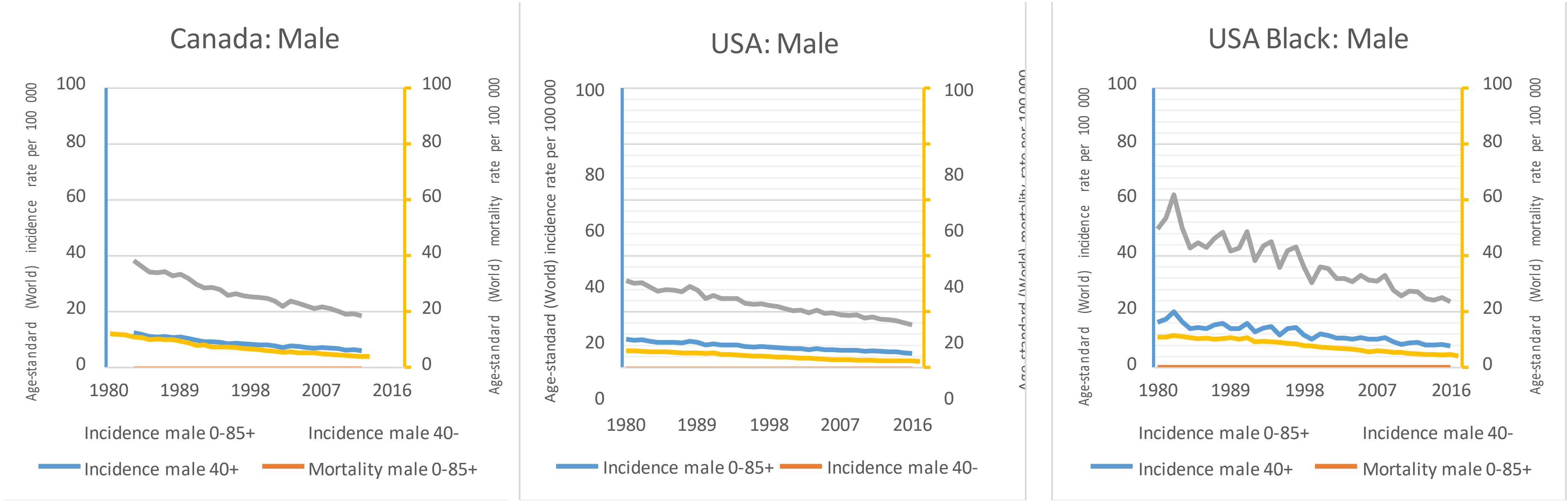

© 2021 Wong MCS et al. *JAMA Network Open.*

Incidence male 40+

Mortality male 0-85+

© 2021 Wong MCS et al. *JAMA Network Open.*

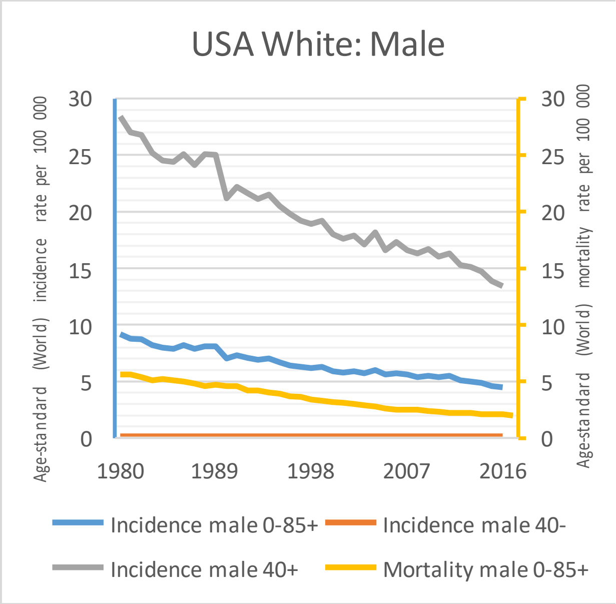

Southern America

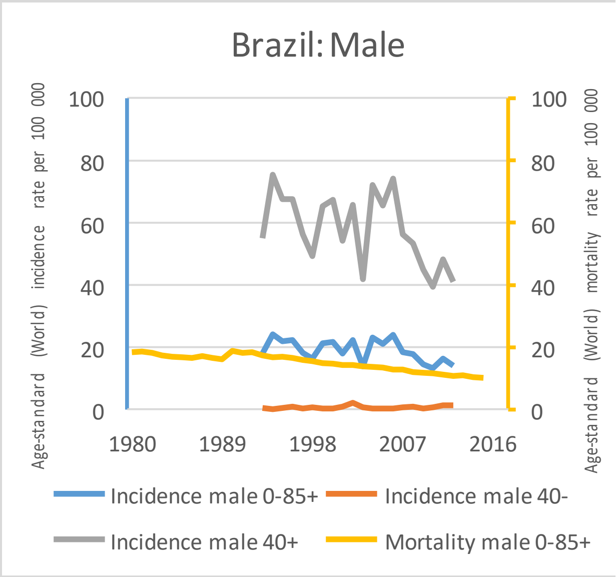

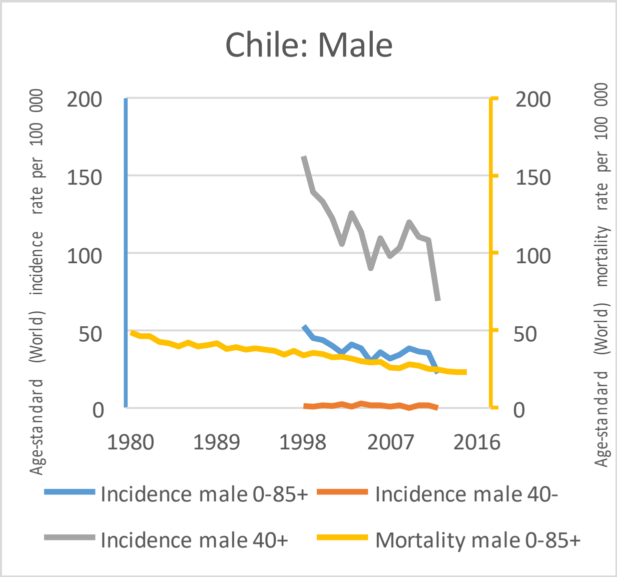

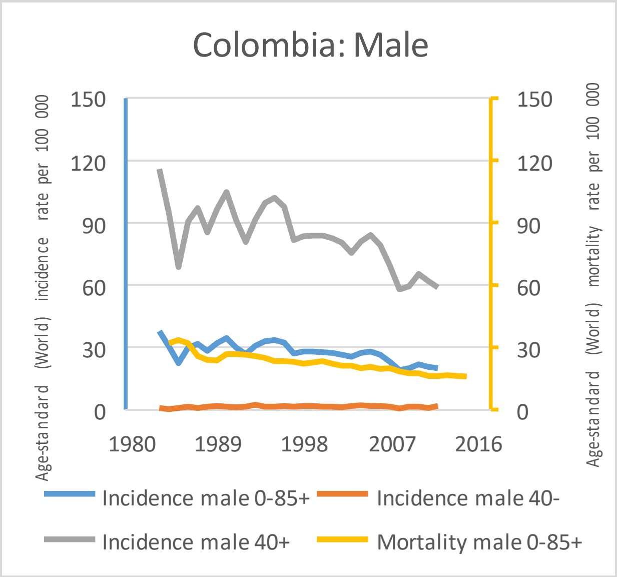

© 2021 Wong MCS et al. *JAMA Network Open.*

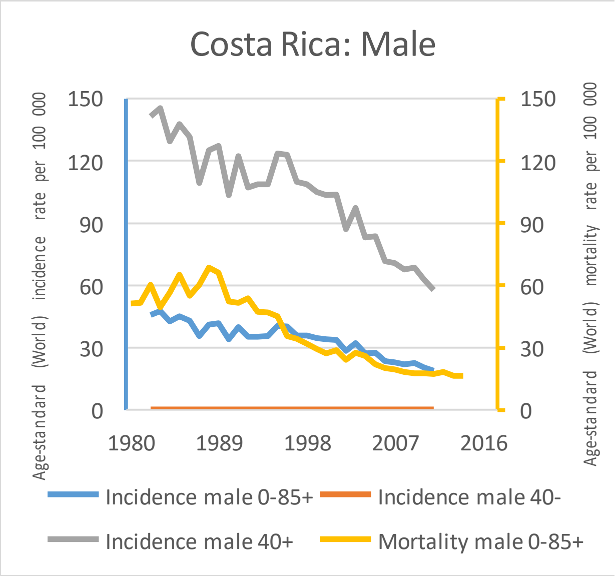

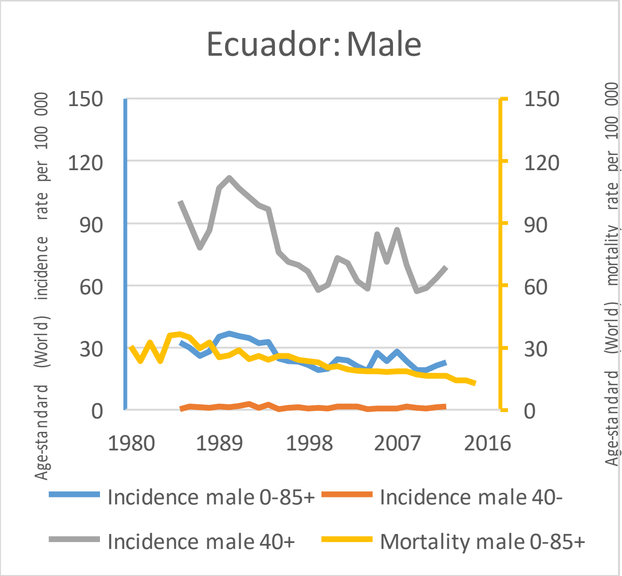

Northern Europe

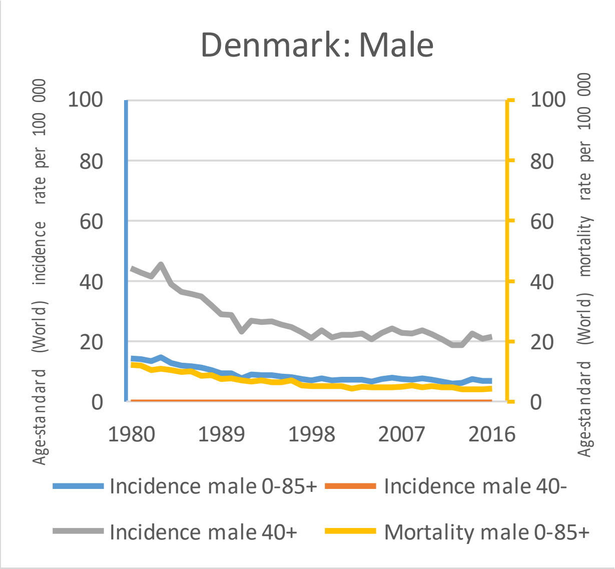

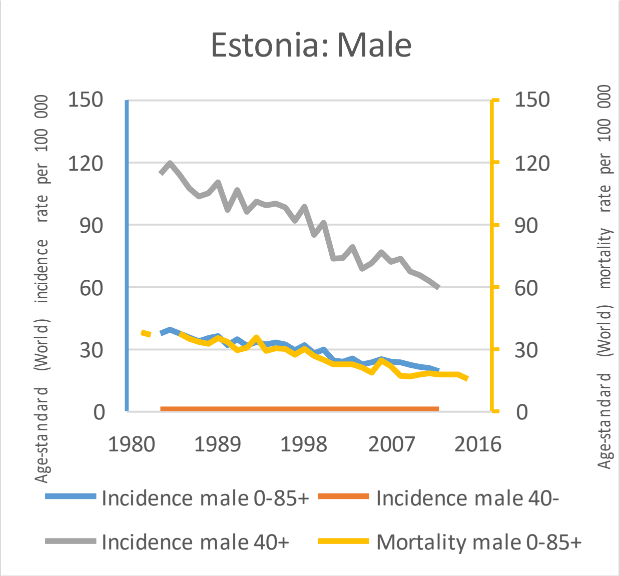

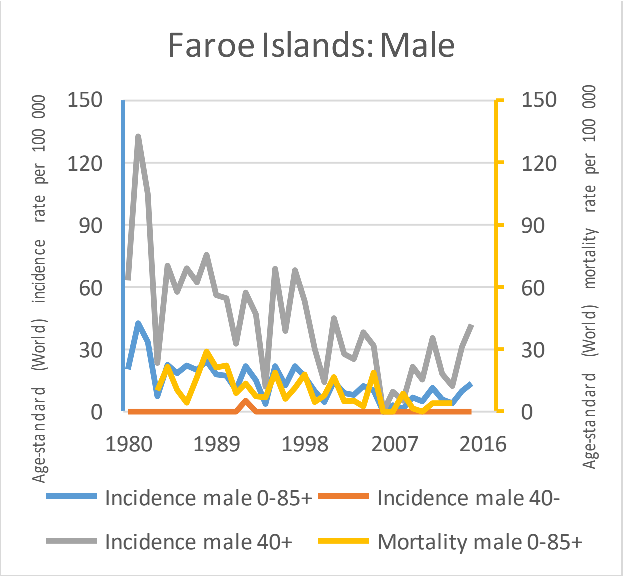

© 2021 Wong MCS et al. *JAMA Network Open.*

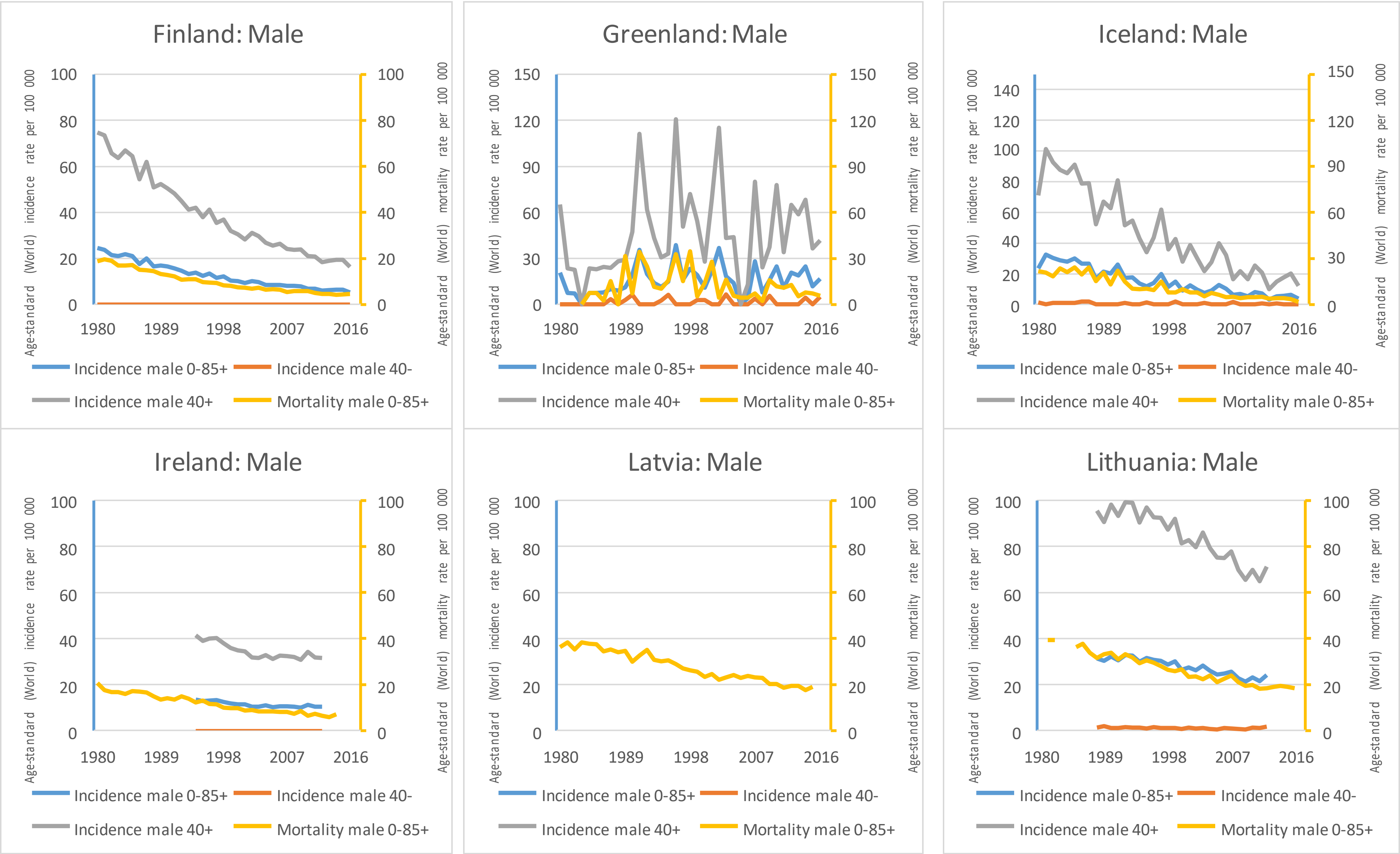

© 2021 Wong MCS et al. *JAMA Network Open*.

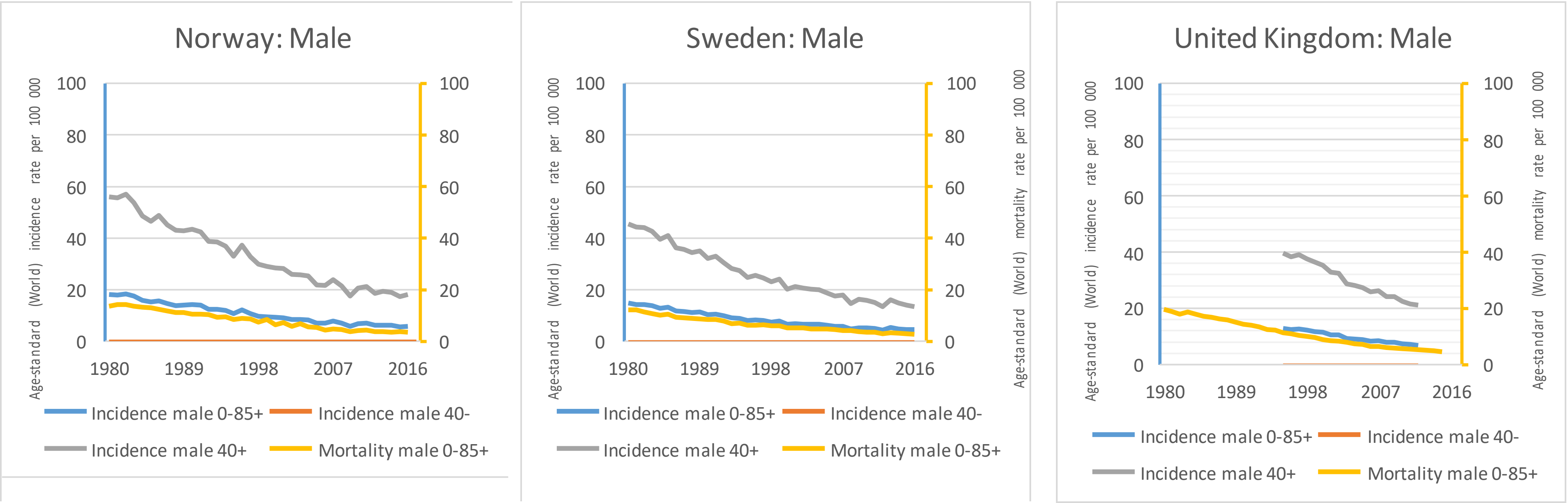

Western Europe

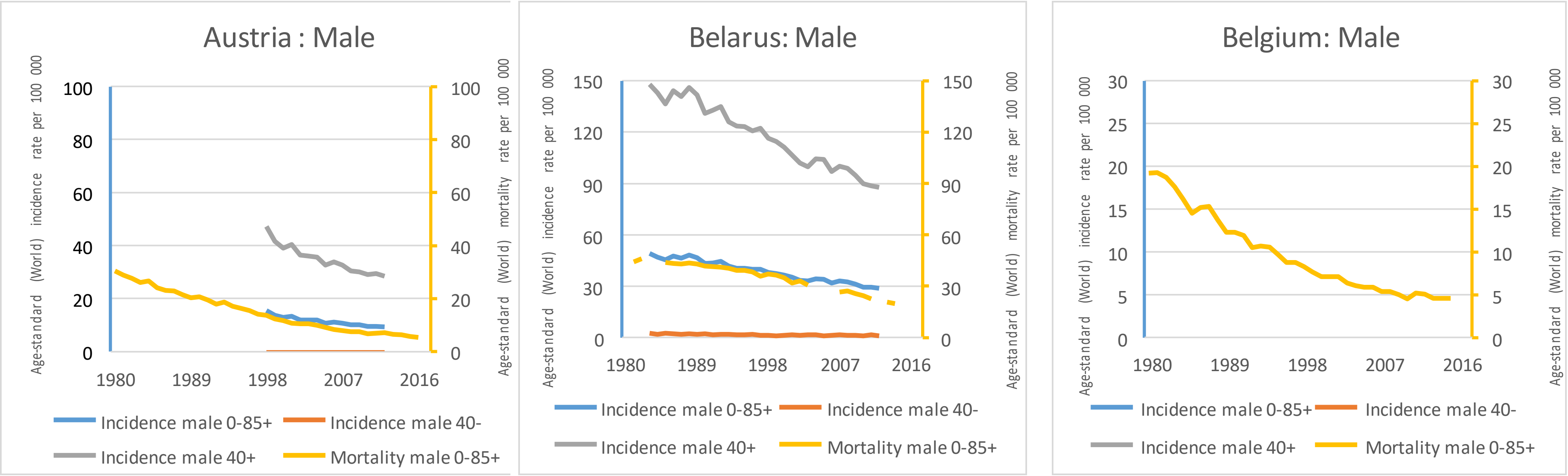

© 2021 Wong MCS et al. *JAMA Network Open.*

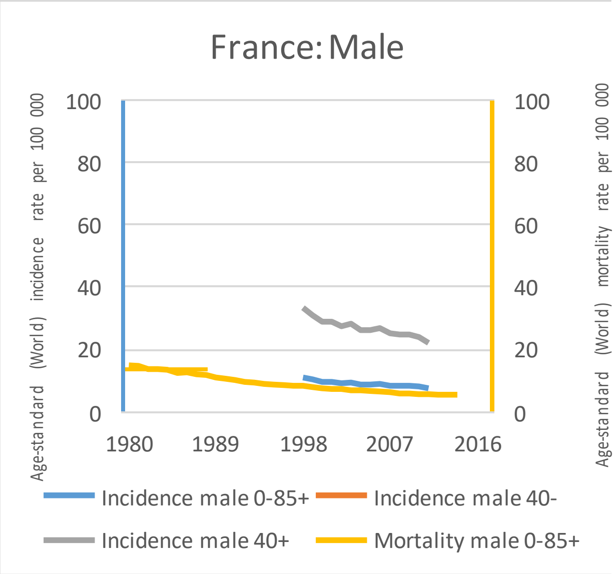

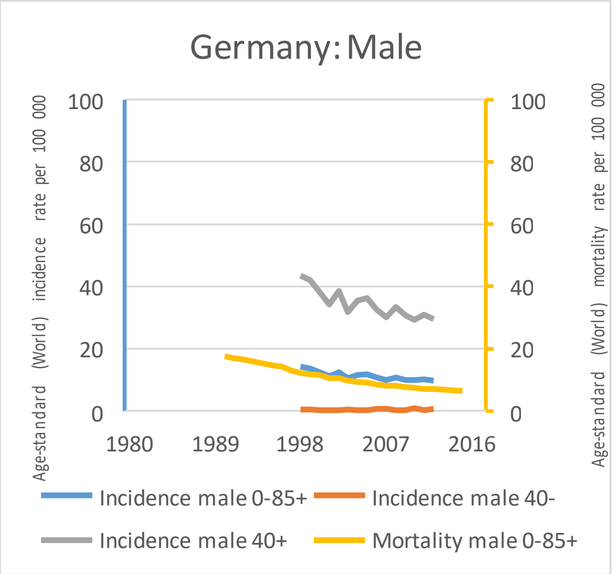

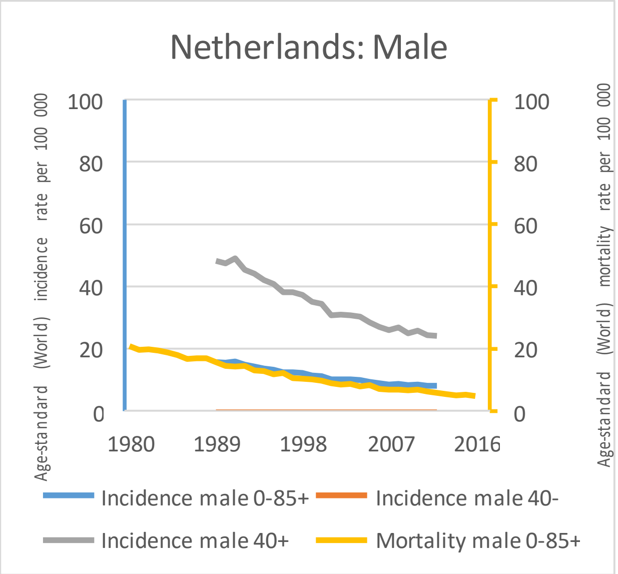

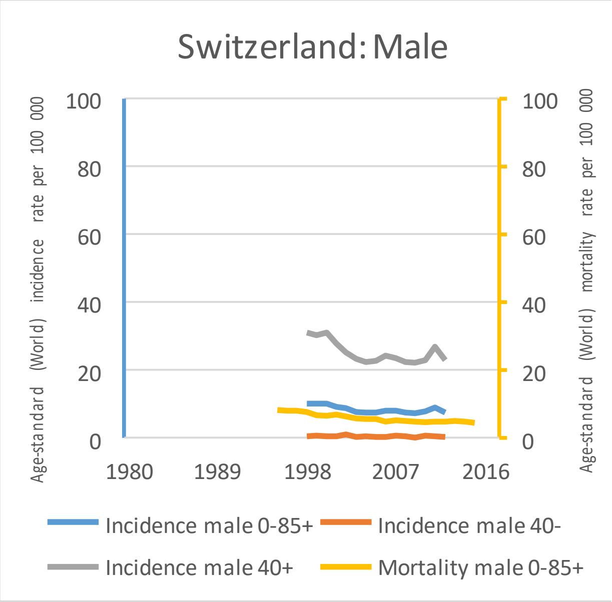

© 2021 Wong MCS et al. *JAMA Network Open.*

Southern Europe

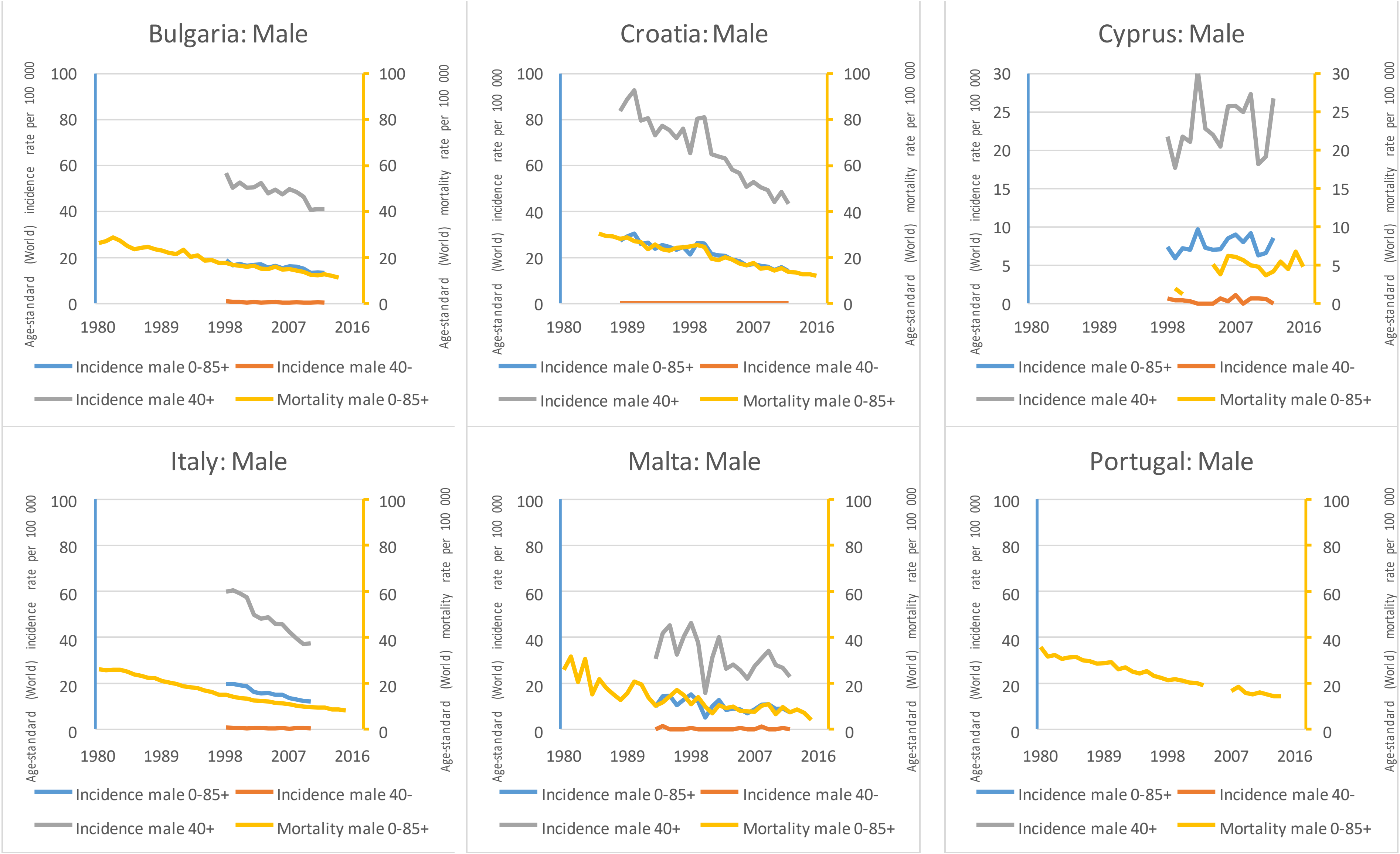

© 2021 Wong MCS et al. *JAMA Network Open.*

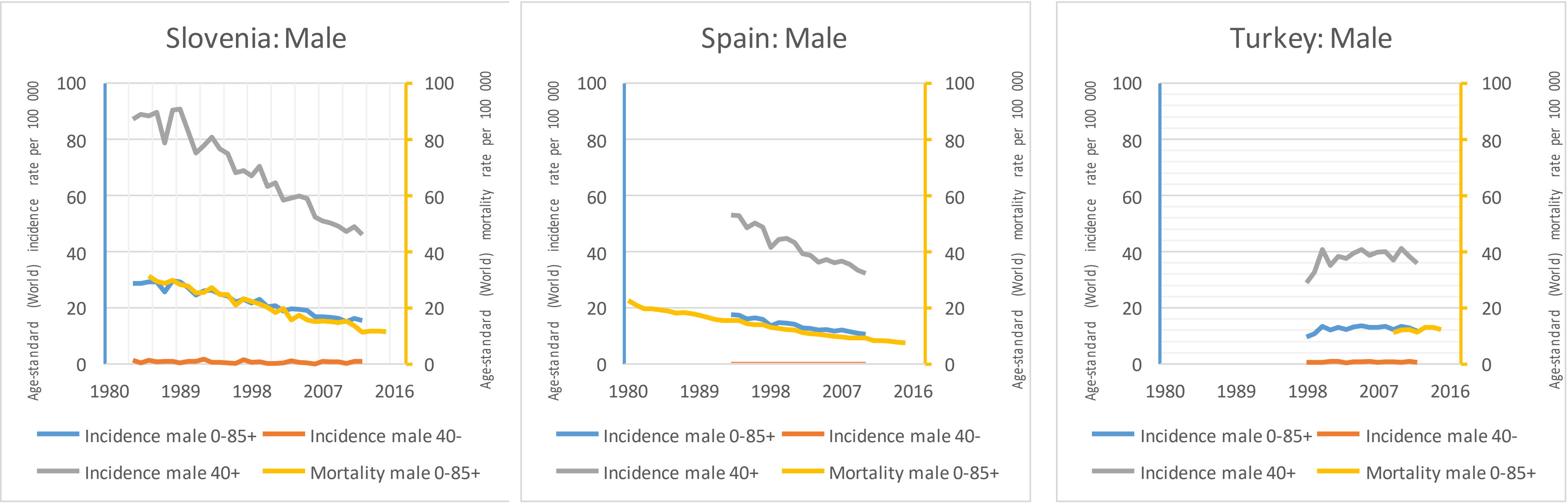

Eastern Europe

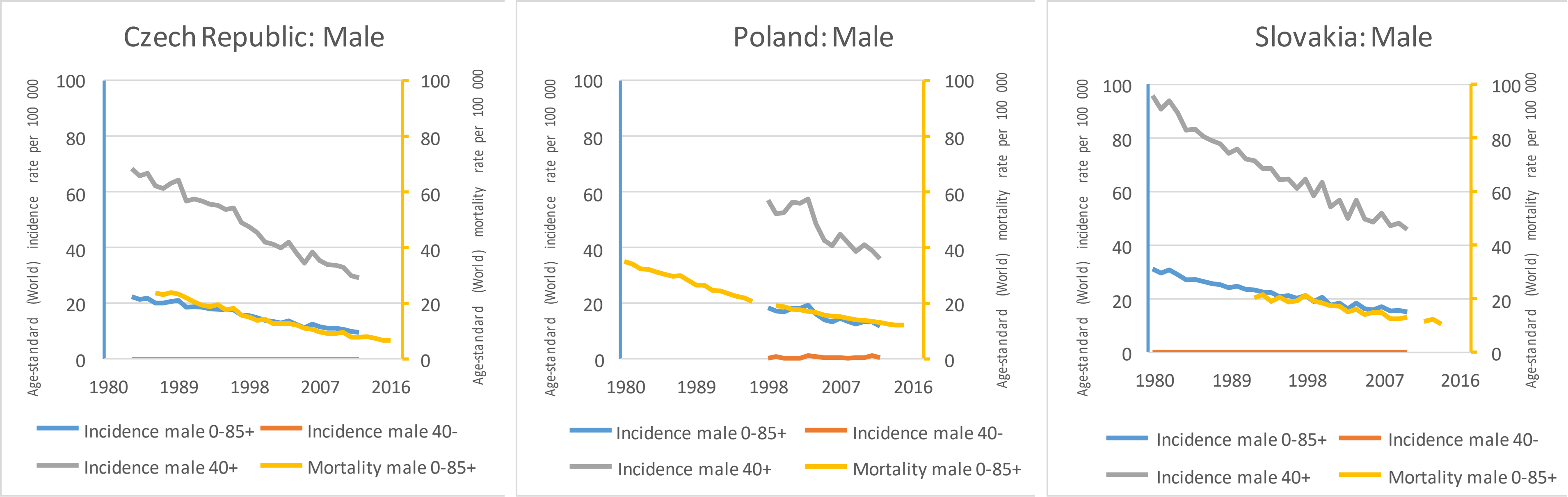

© 2021 Wong MCS et al. *JAMA Network Open.*

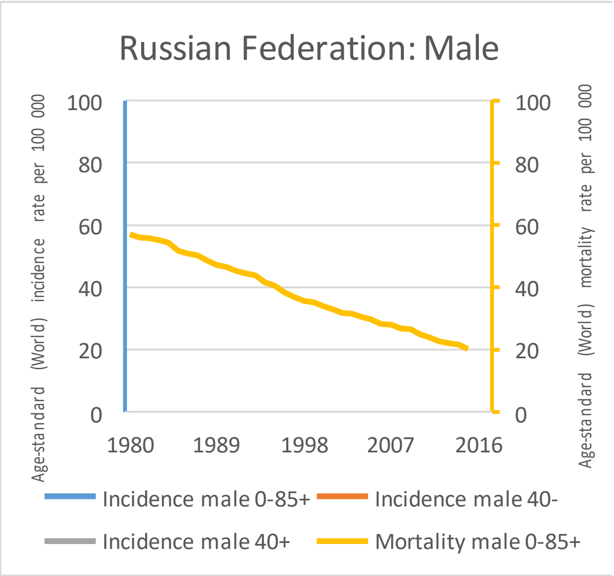

Africa

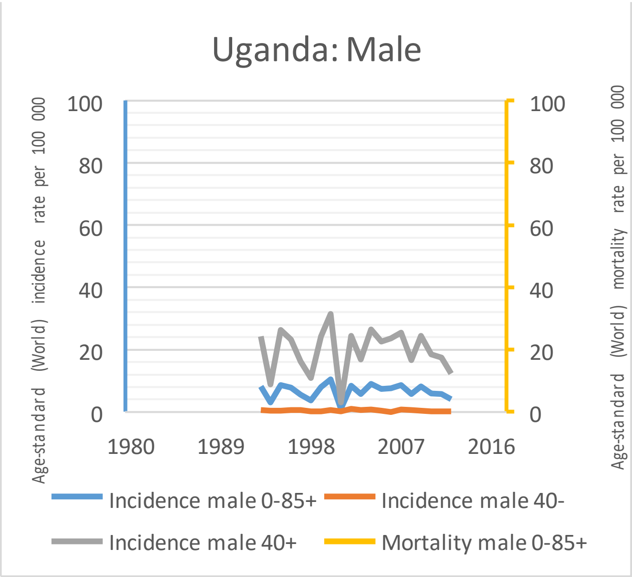

© 2021 Wong MCS et al. *JAMA Network Open.*

female

Asia

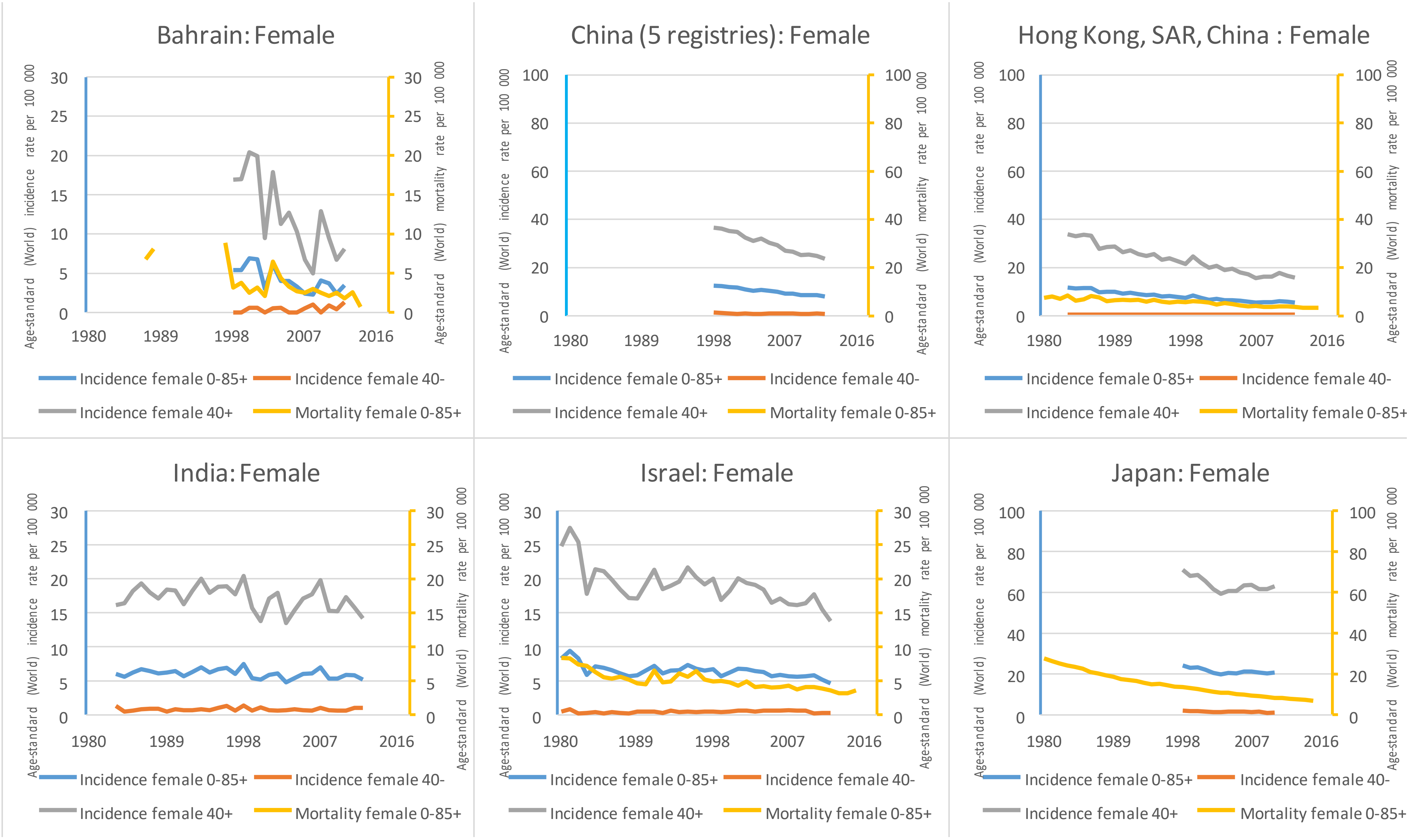

© 2021 Wong MCS et al. *JAMA Network Open.*

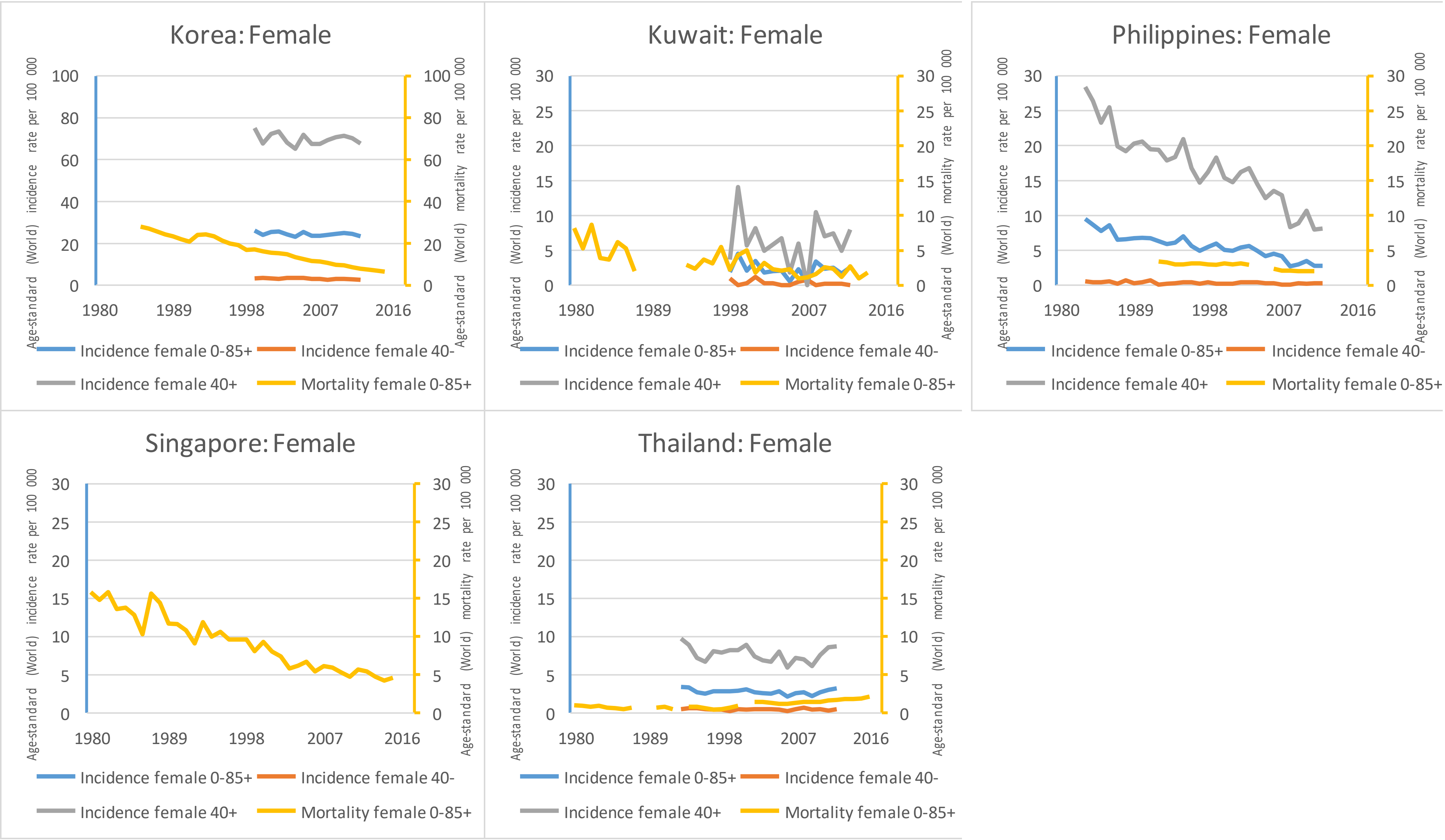

© 2021 Wong MCS et al. *JAMA Network Open.*

Oceania

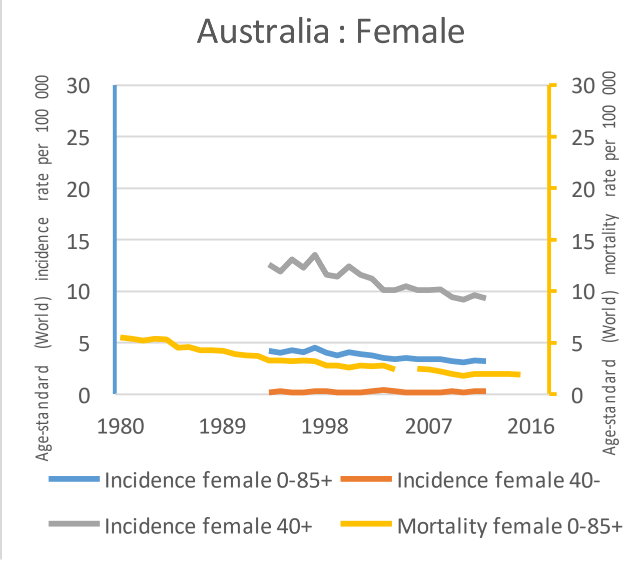

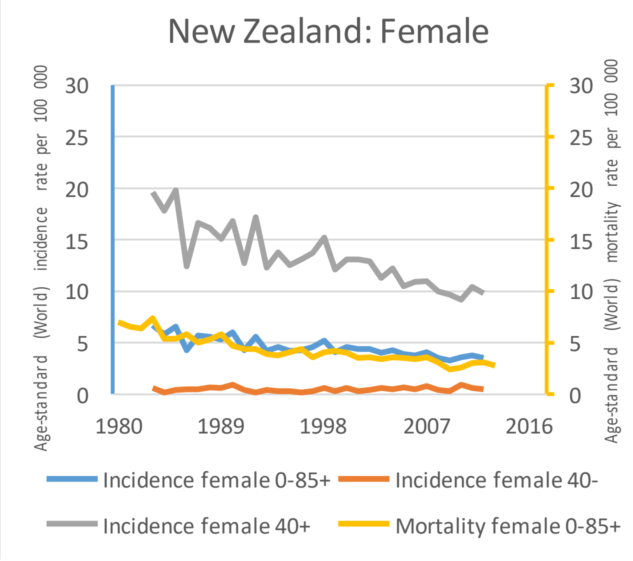

Northern America

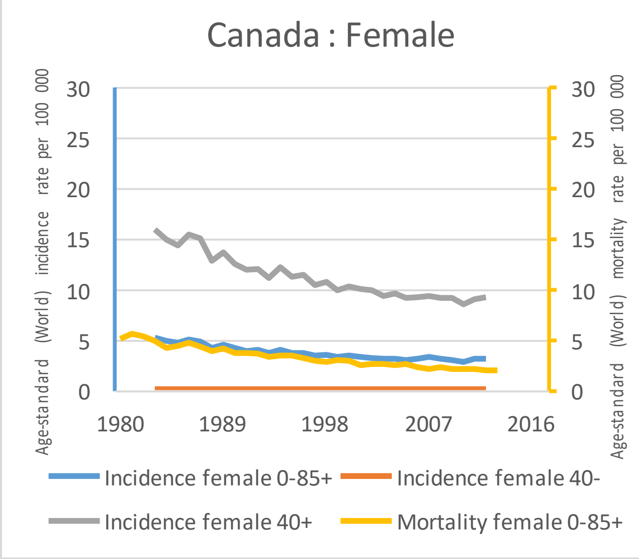

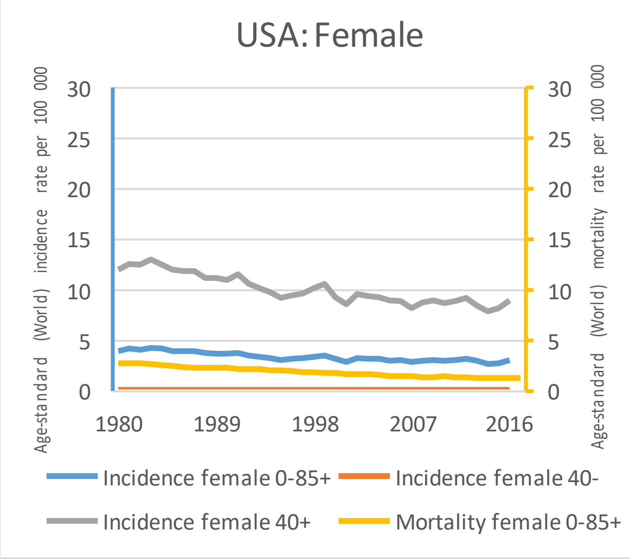

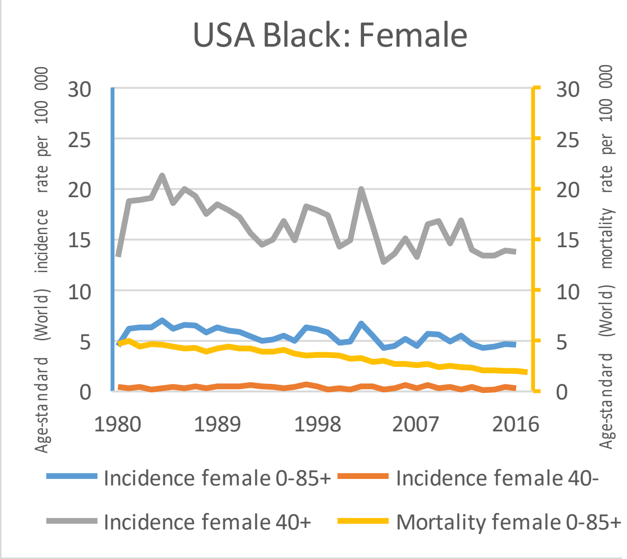

© 2021 Wong MCS et al. *JAMA Network Open.*

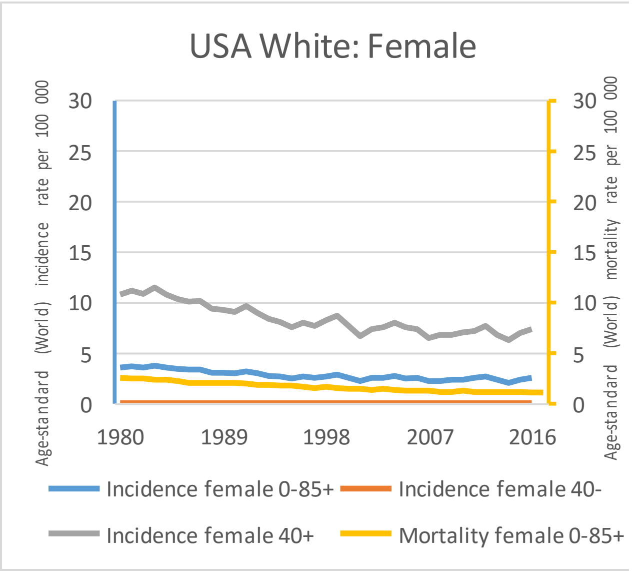

Southern America

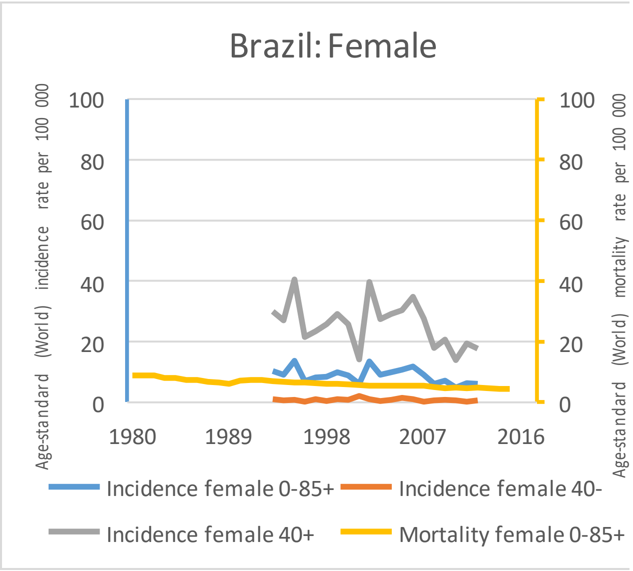

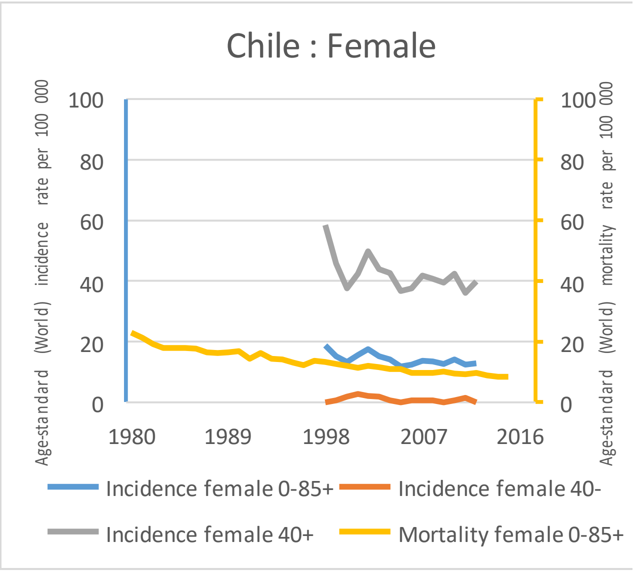

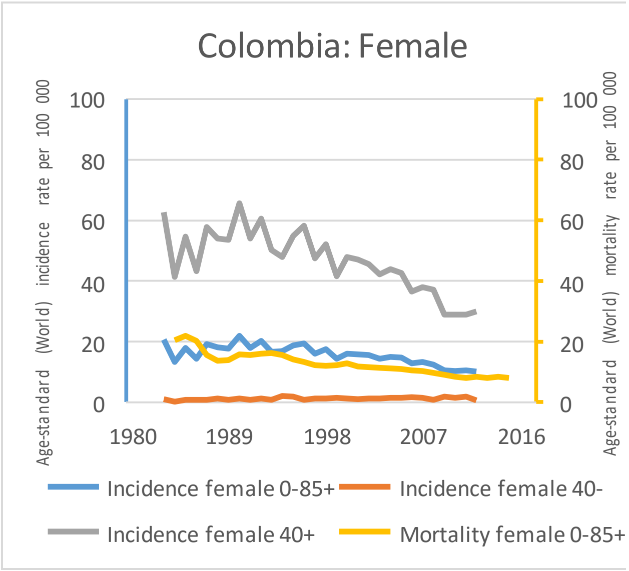

© 2021 Wong MCS et al. *JAMA Network Open.*

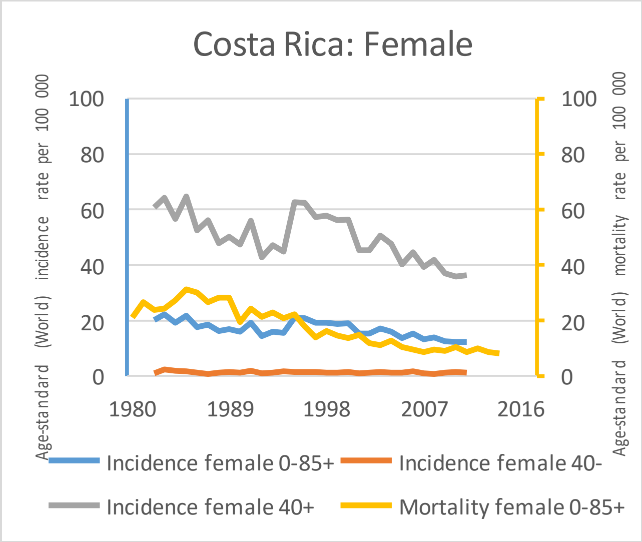

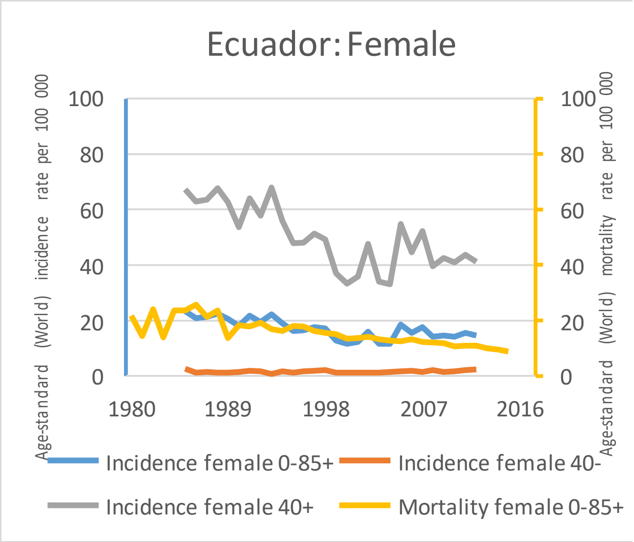

Northern Europe

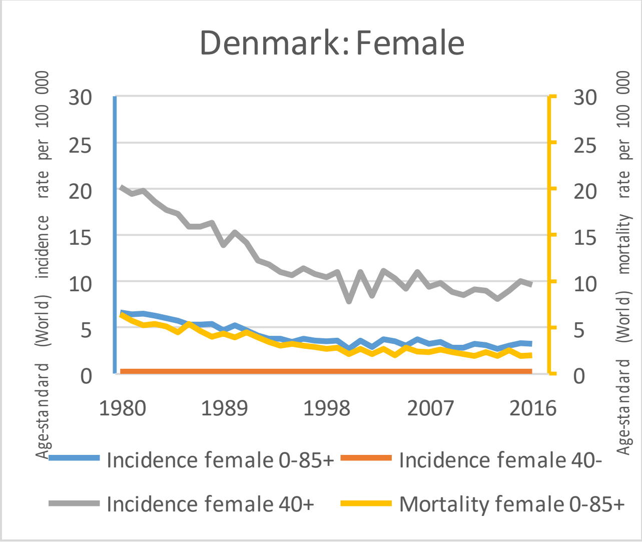

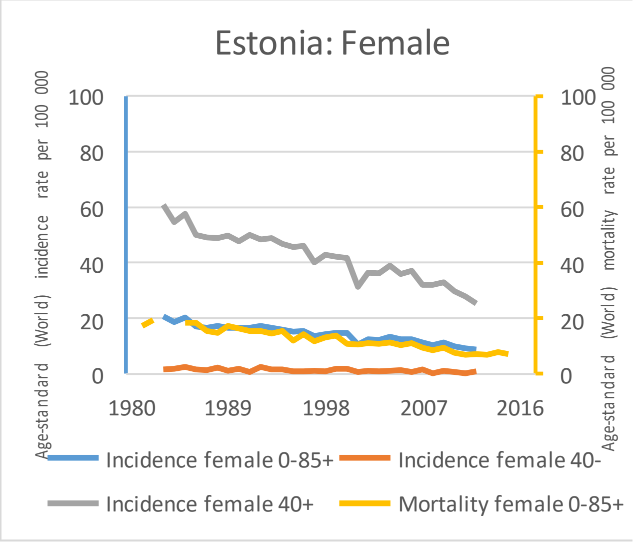

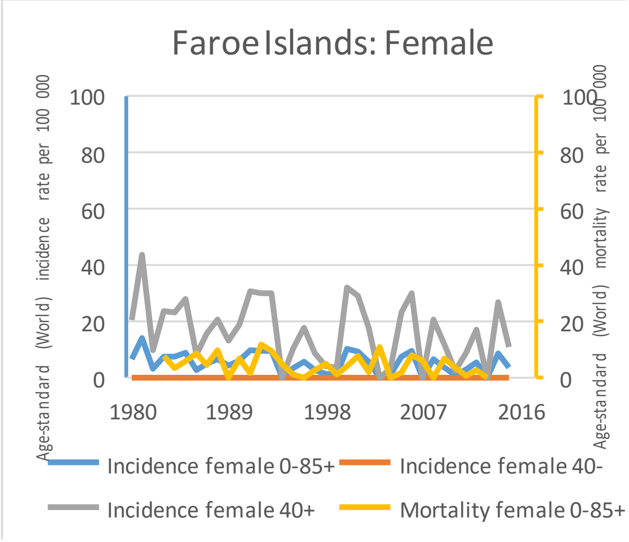

© 2021 Wong MCS et al. *JAMA Network Open.*

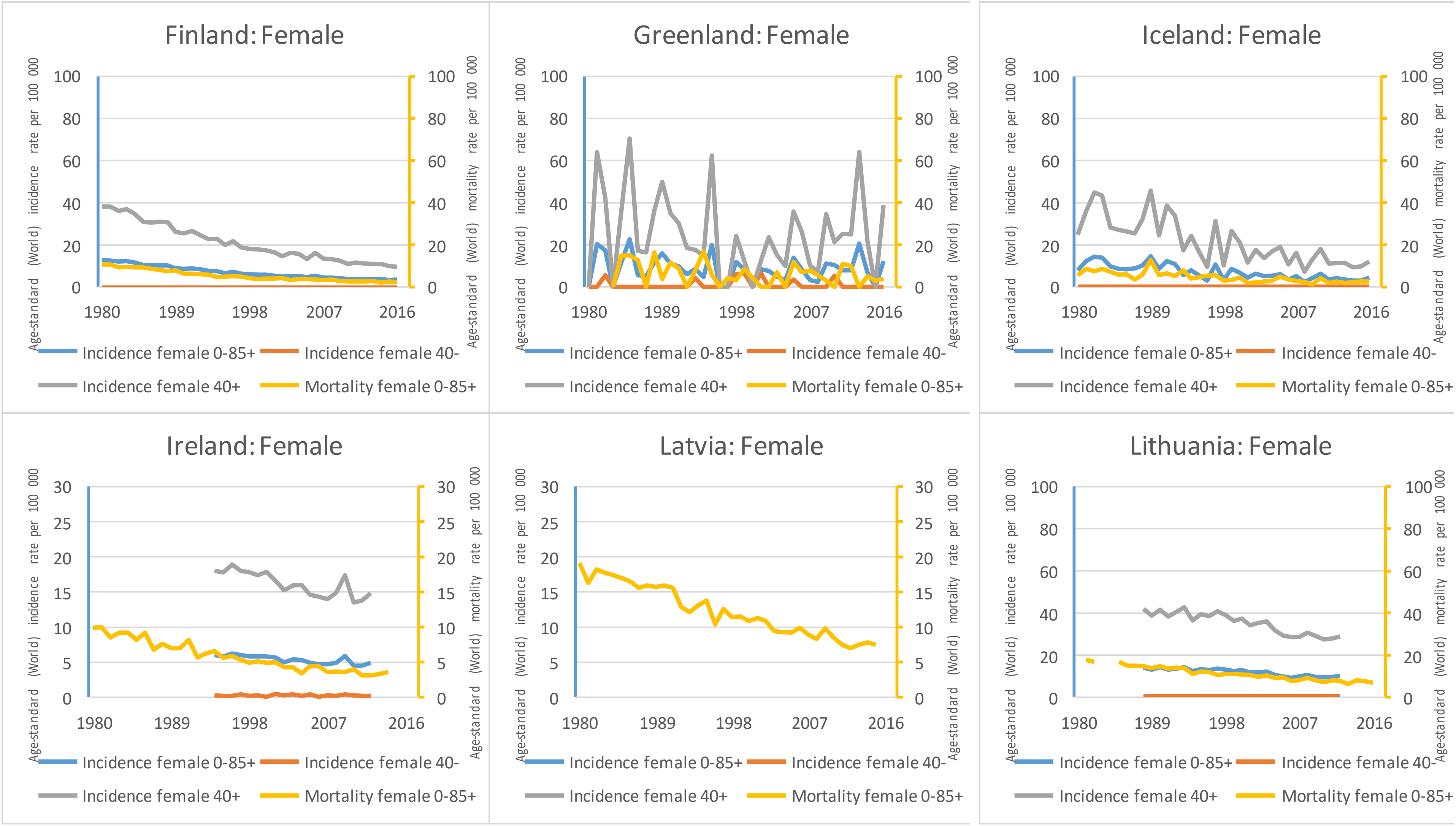

© 2021 Wong MCS et al. *JAMA Network Open.*

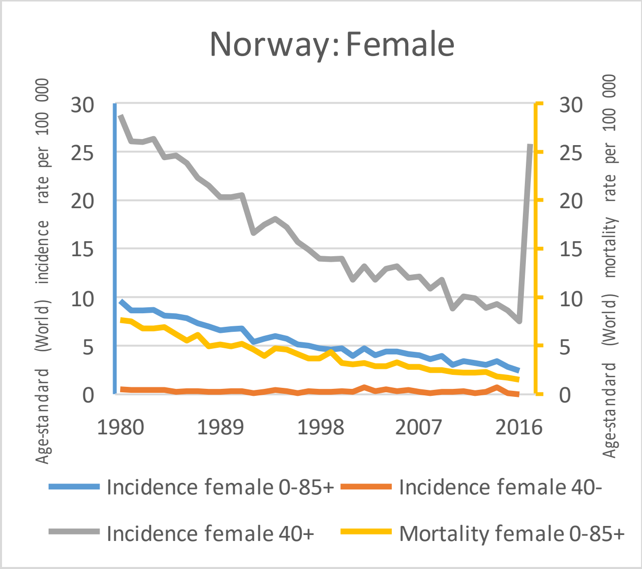

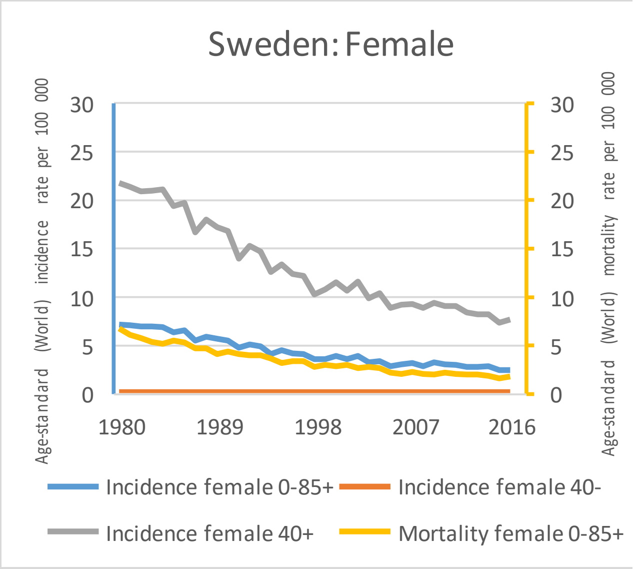

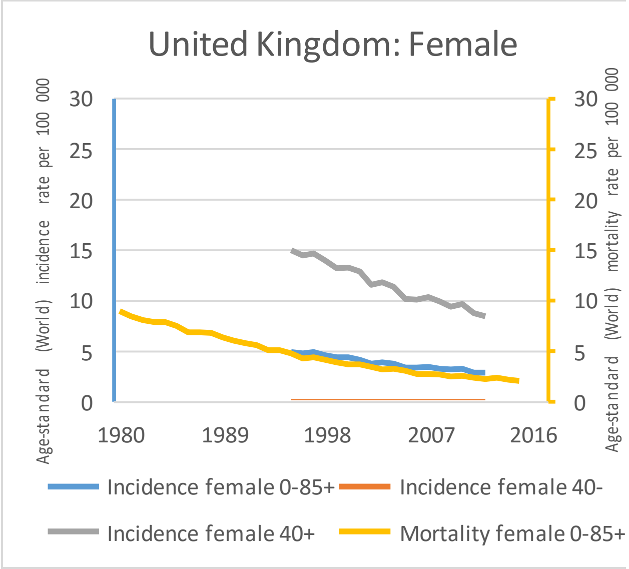

Western Europe

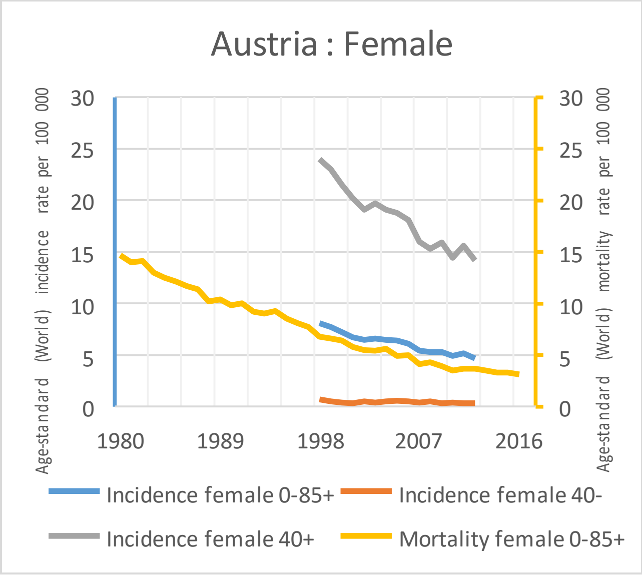

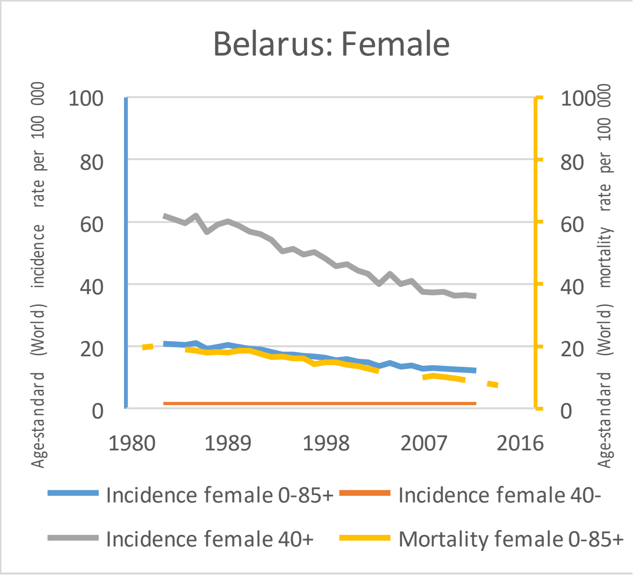

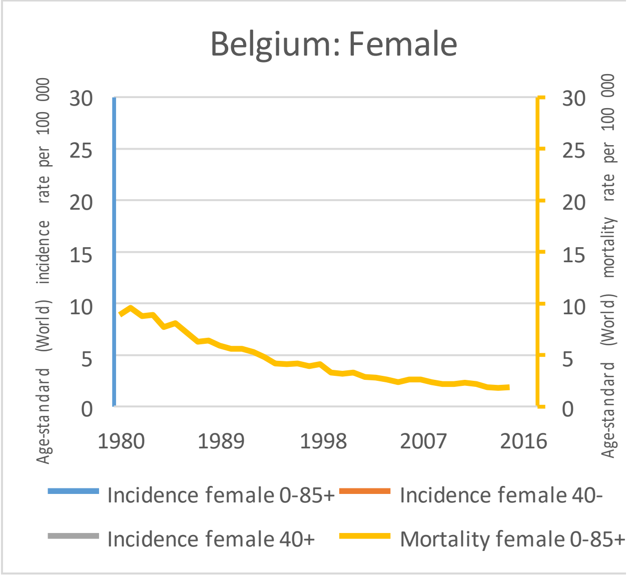

© 2021 Wong MCS et al. *JAMA Network Open.*

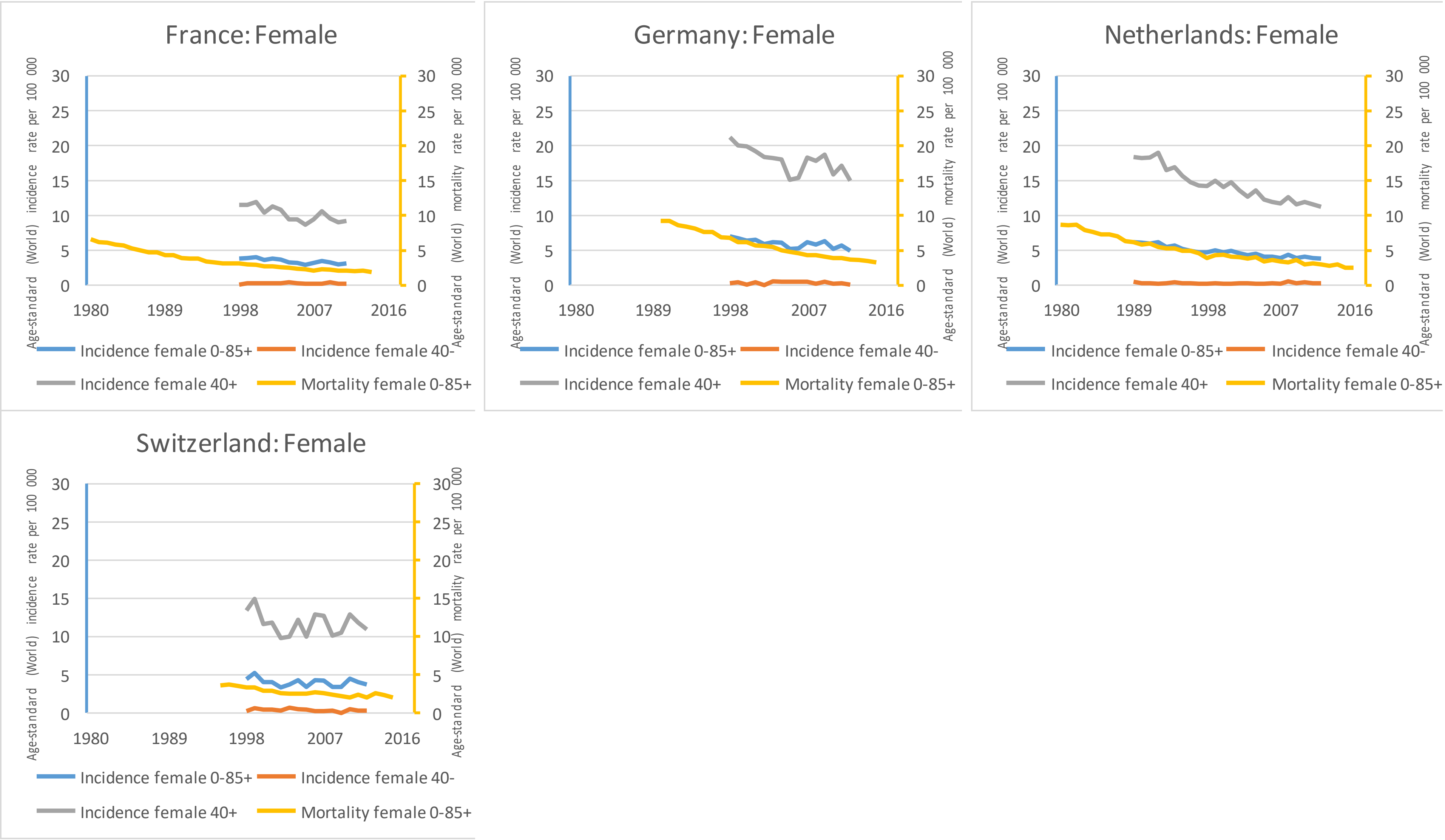

© 2021 Wong MCS et al. *JAMA Network Open.*

Southern Europe

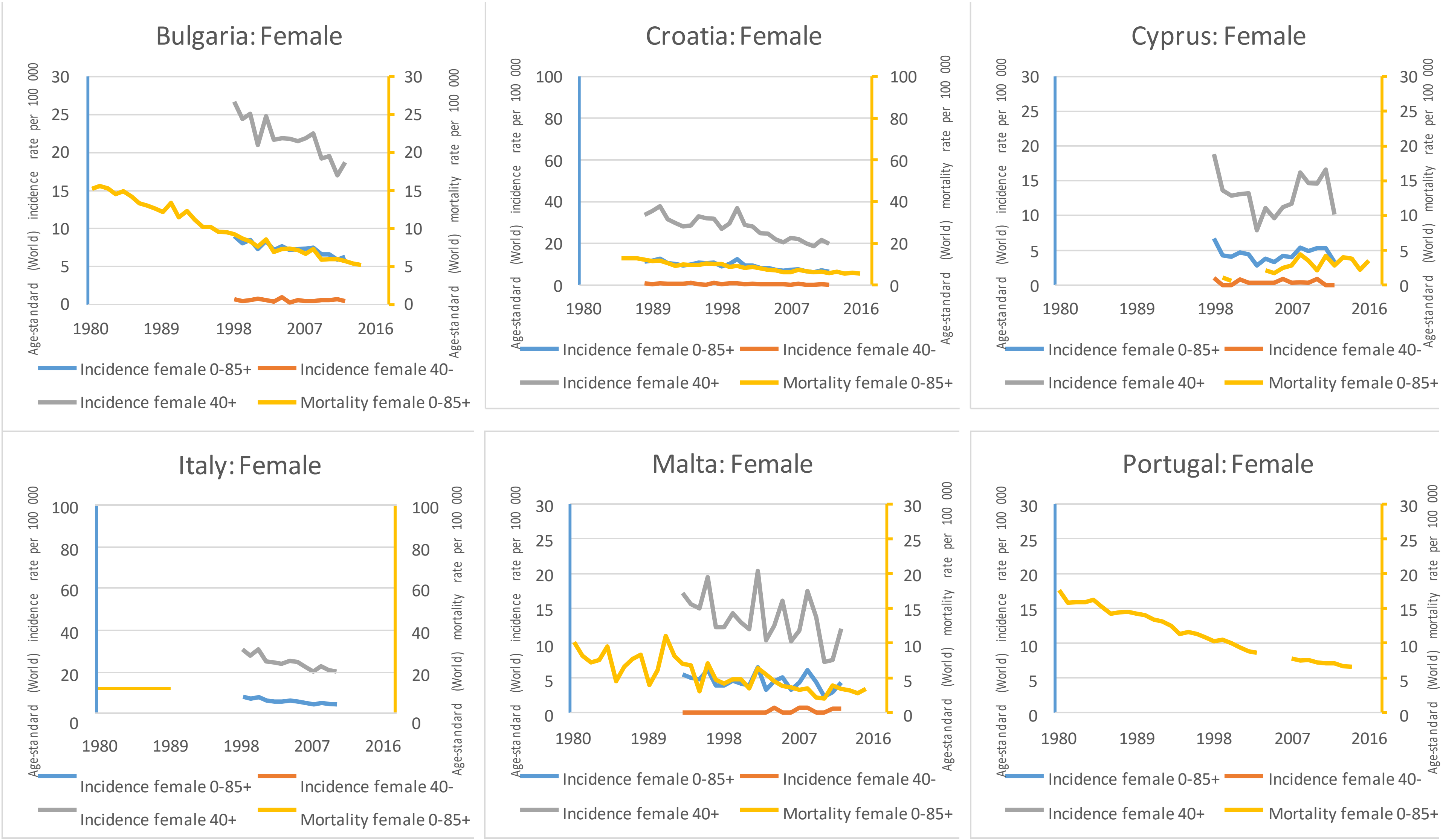

© 2021 Wong MCS et al. *JAMA Network Open.*

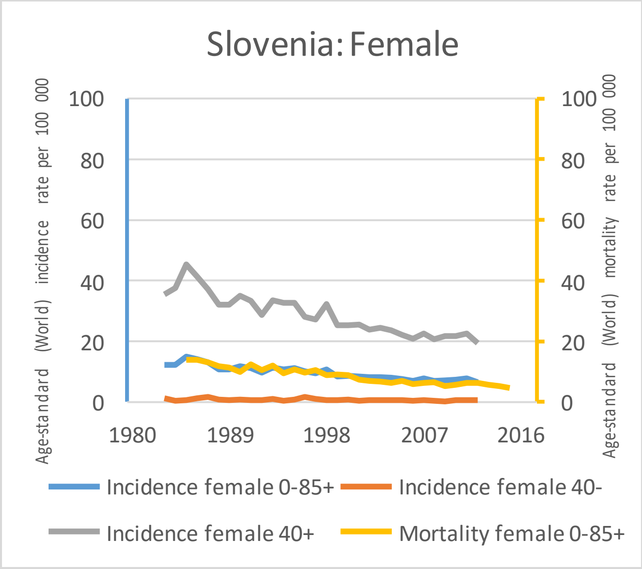

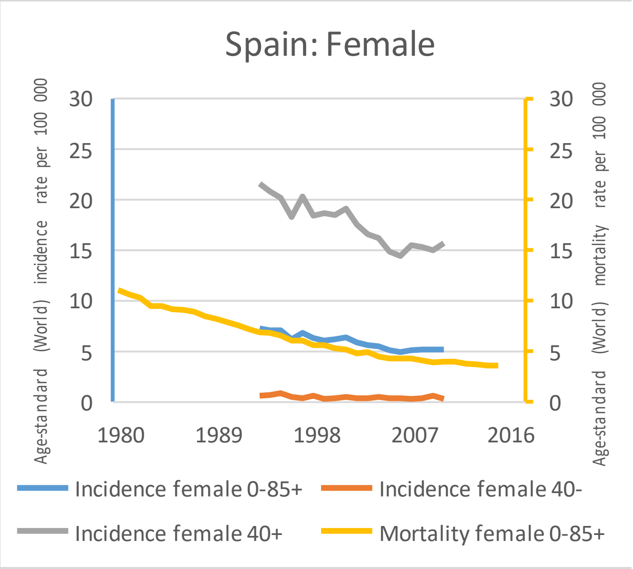

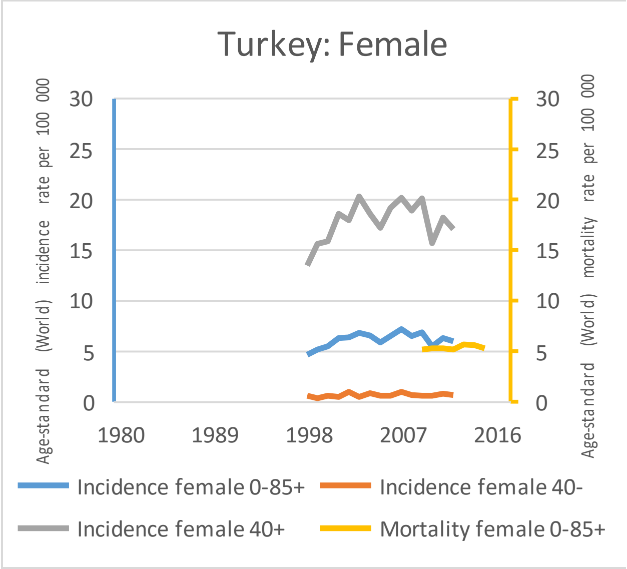

Eastern Europe

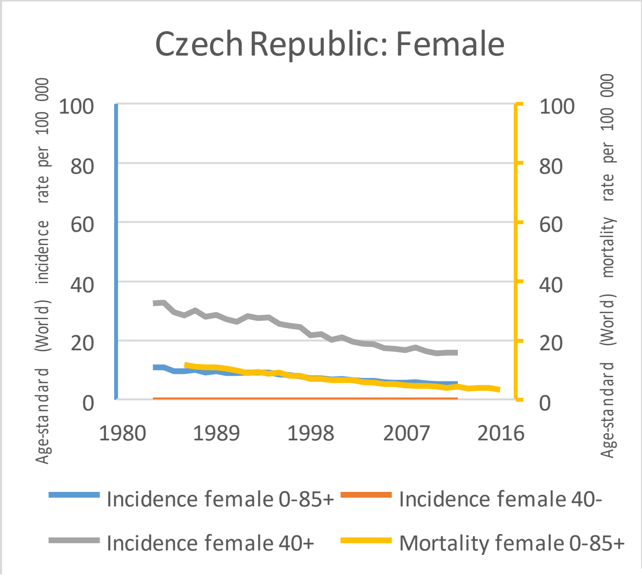

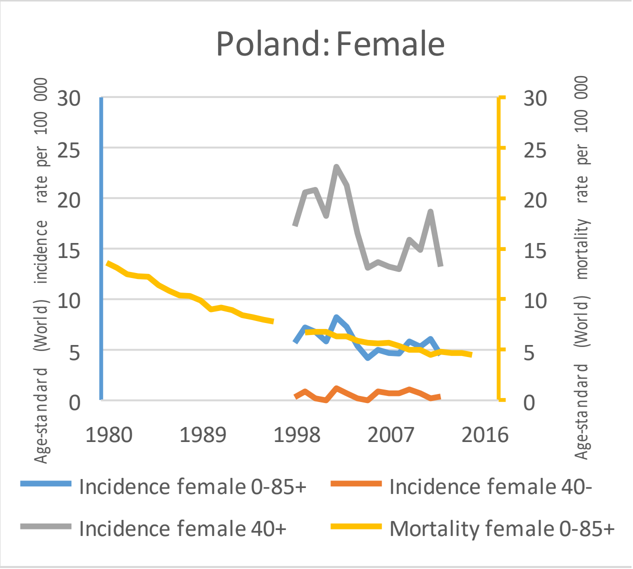

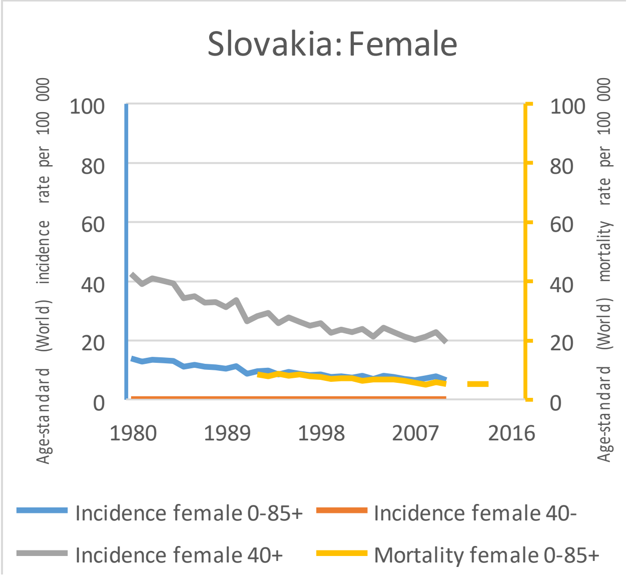

© 2021 Wong MCS et al. *JAMA Network Open.*

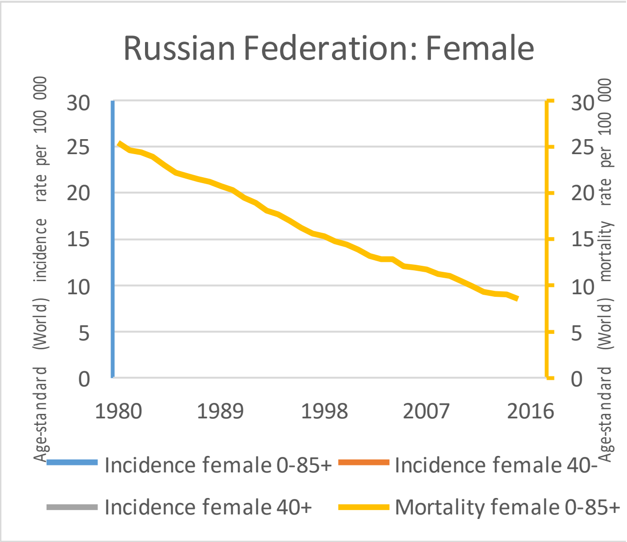

Africa

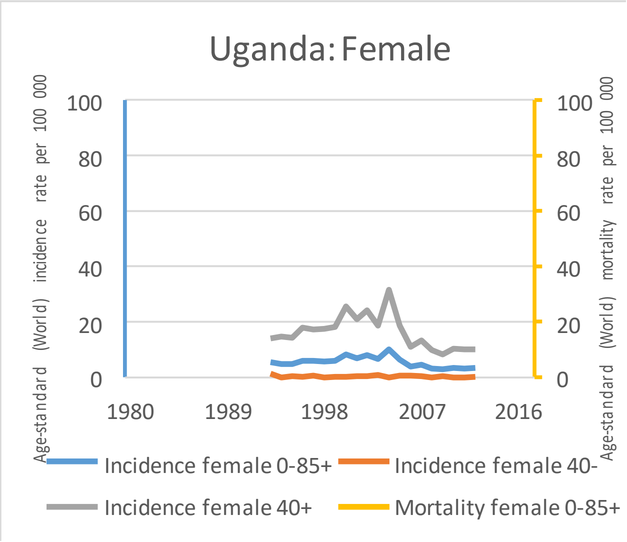

© 2021 Wong MCS et al. *JAMA Network Open.*

eFigure 2: The graphs of the joinpoint regression output a.) Incidence male all ages

Asia

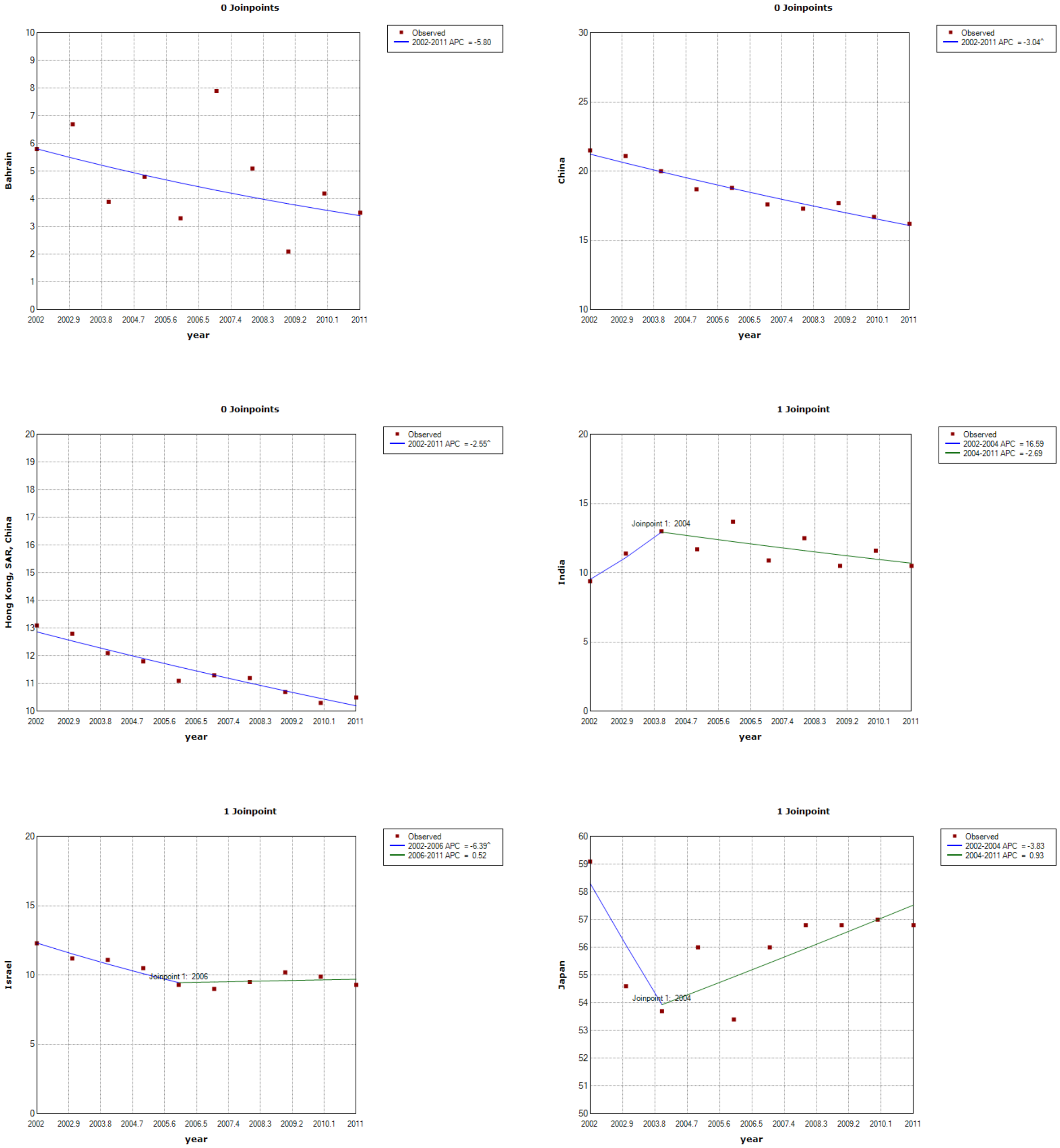

© 2021 Wong MCS et al. *JAMA Network Open.*

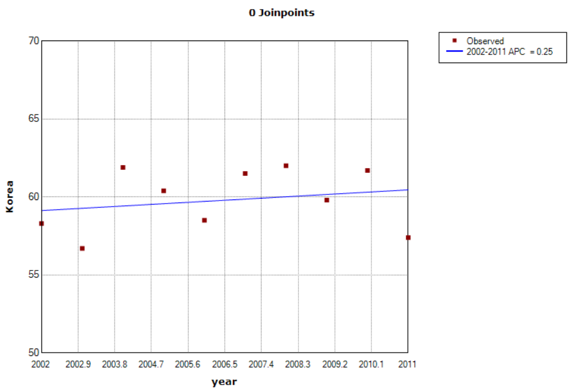

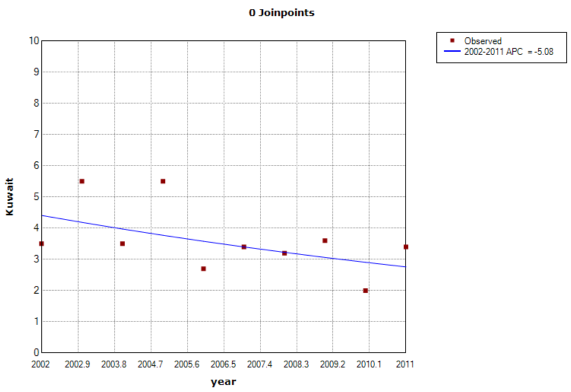

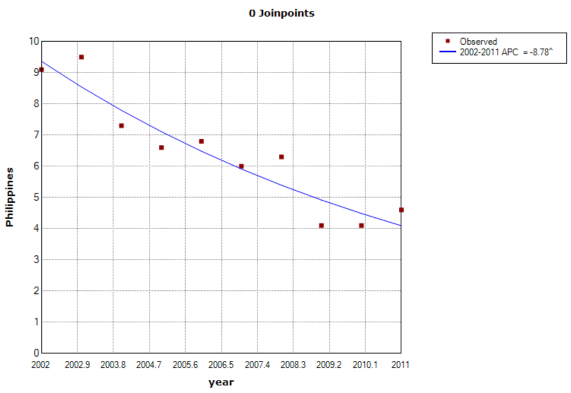

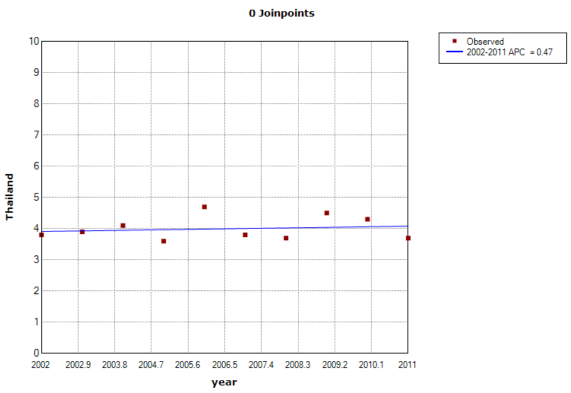

Oceania

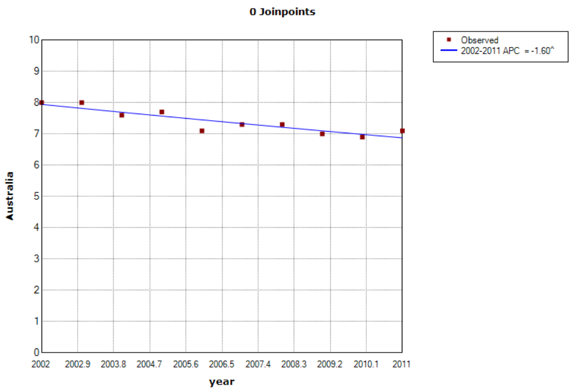

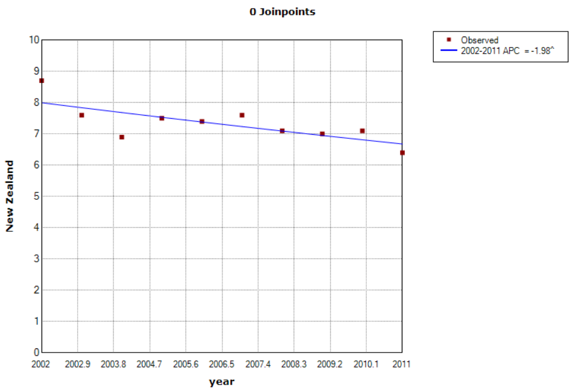

© 2021 Wong MCS et al. *JAMA Network Open.*

Northern America

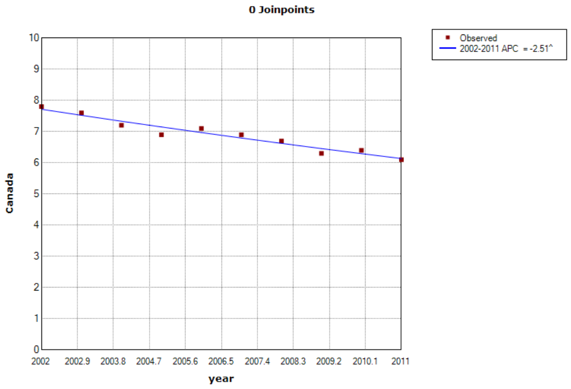

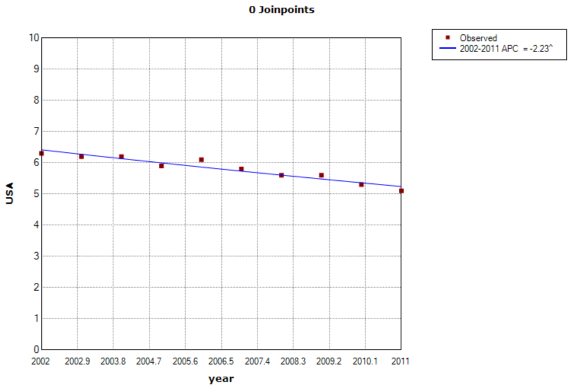

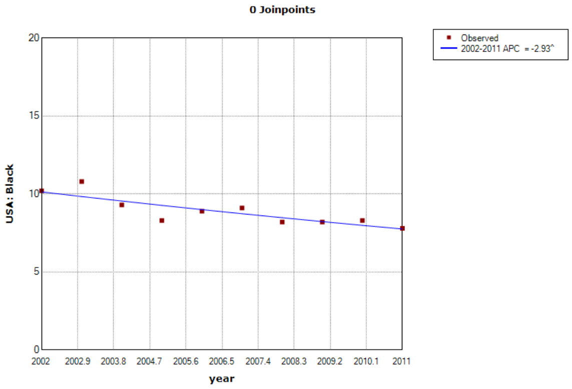

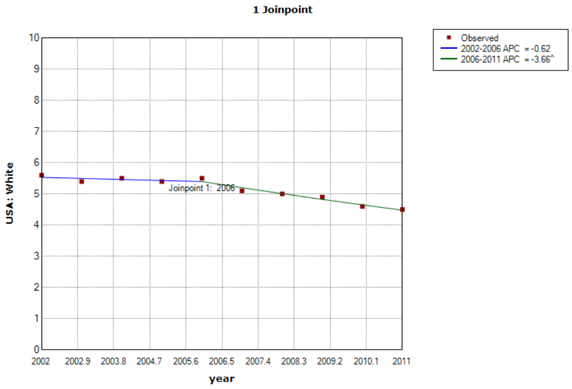

Southern America

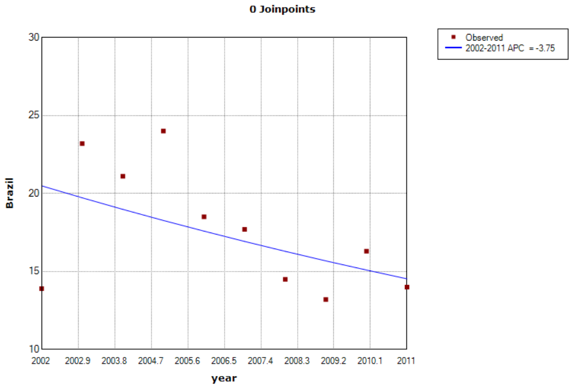

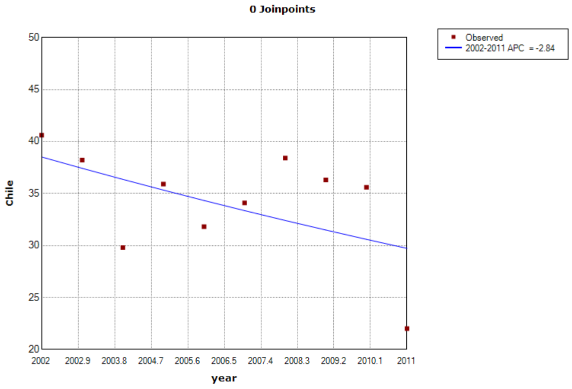

© 2021 Wong MCS et al. *JAMA Network Open.*

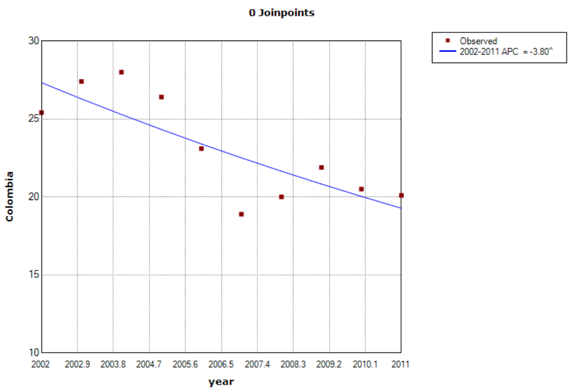

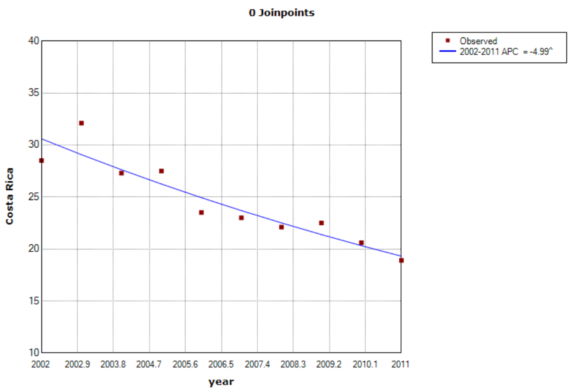

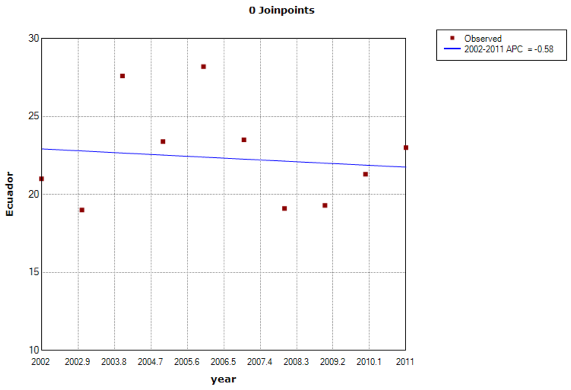

Northern Europe

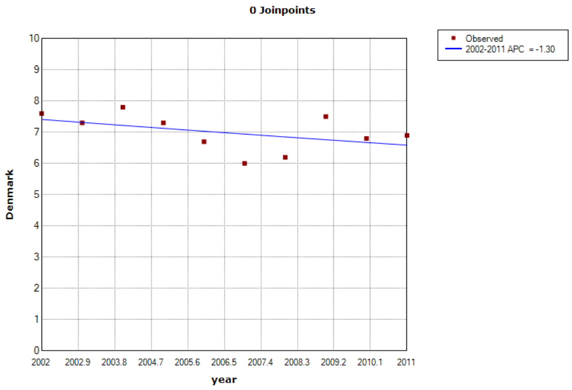

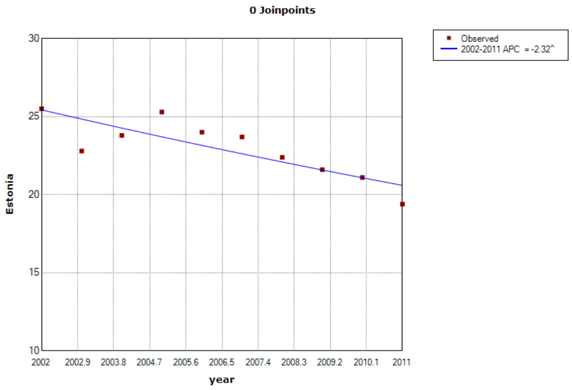

© 2021 Wong MCS et al. *JAMA Network Open.*

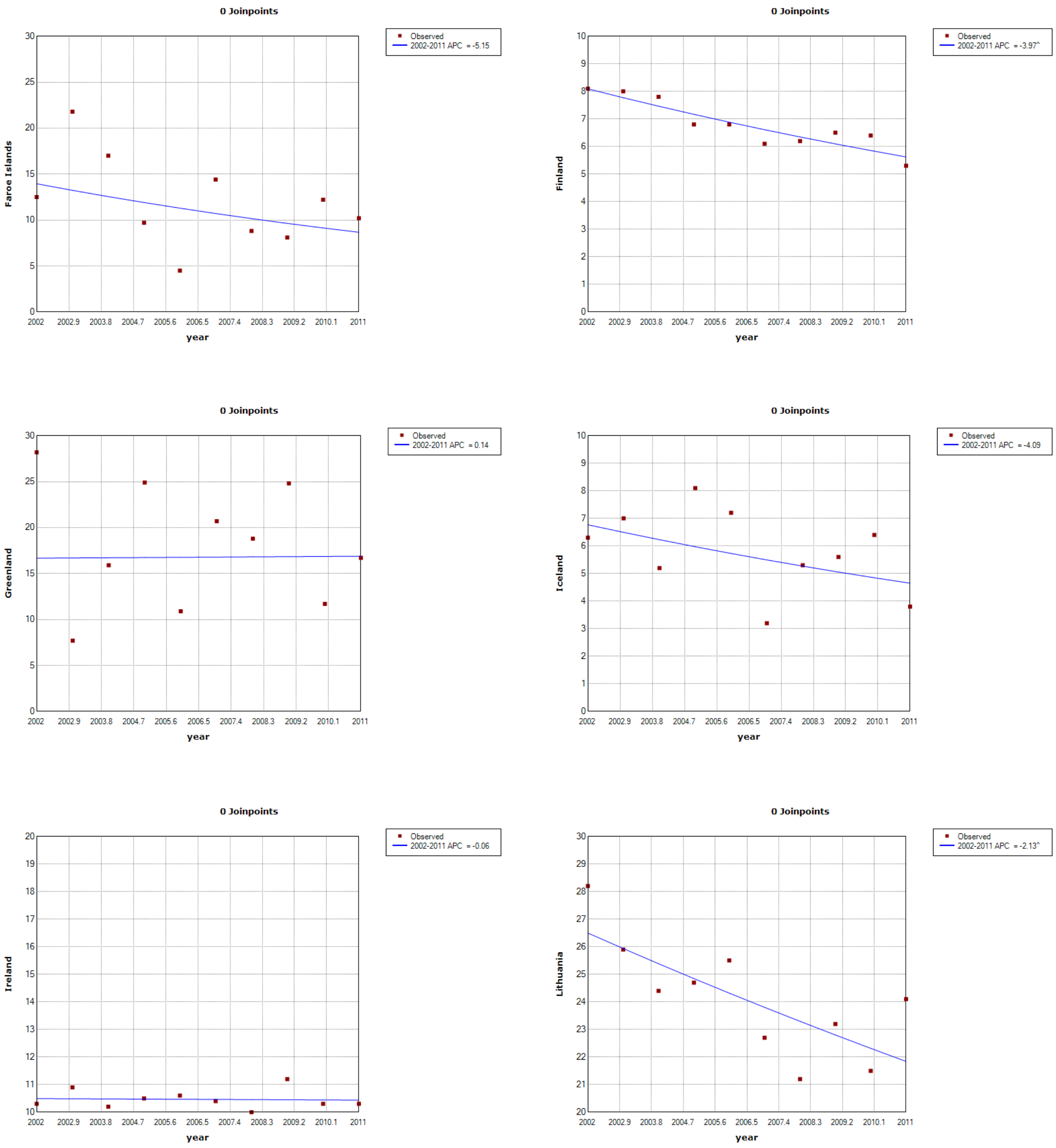

© 2021 Wong MCS et al. *JAMA Network Open.*

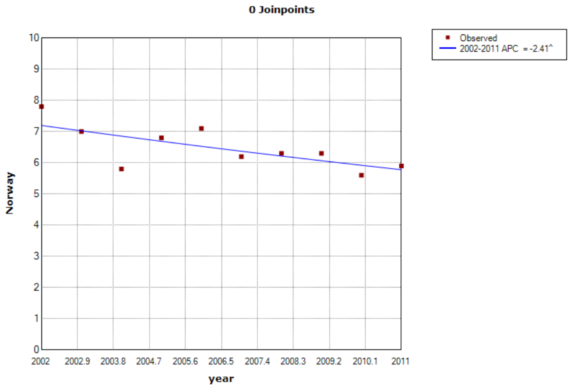

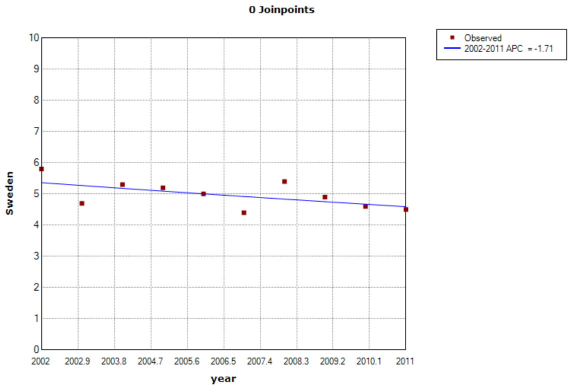

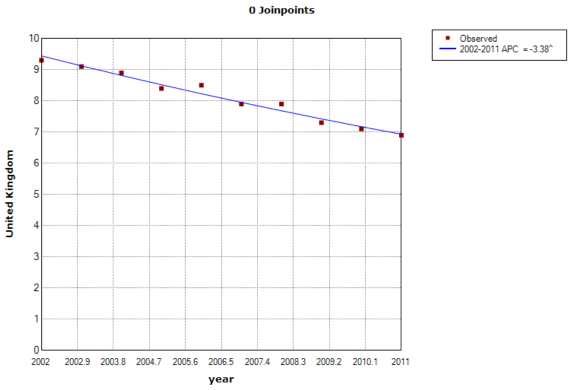

Western Europe

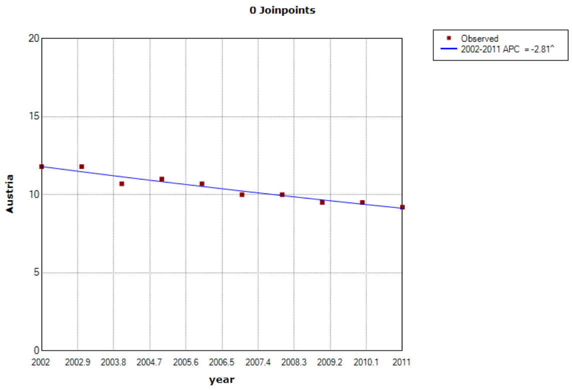

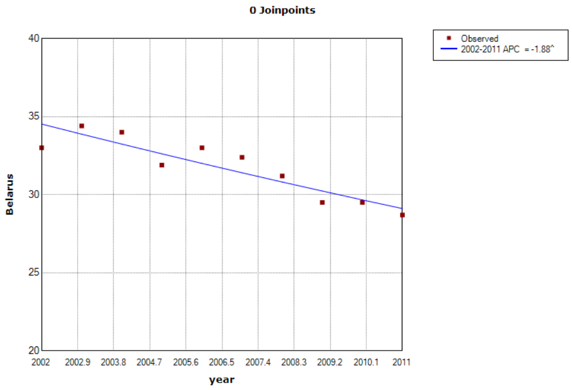

© 2021 Wong MCS et al. *JAMA Network Open.*

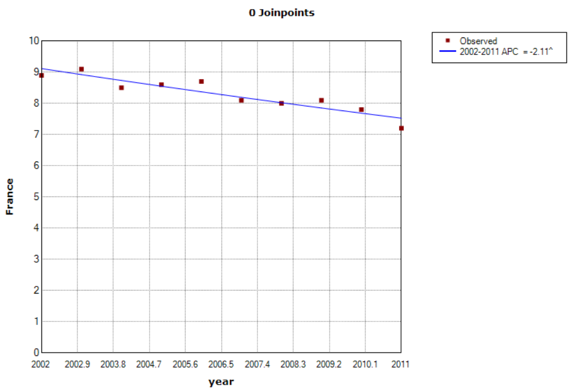

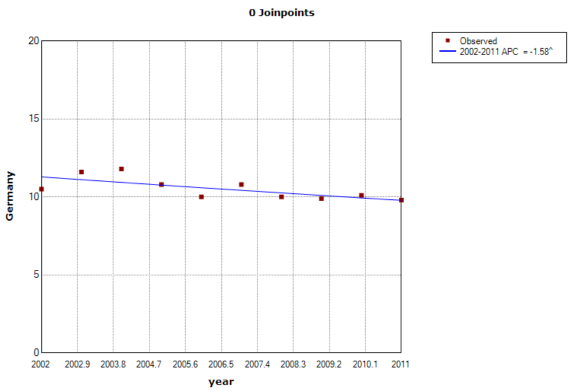

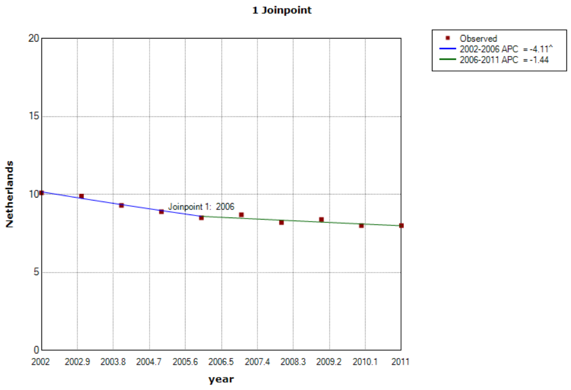

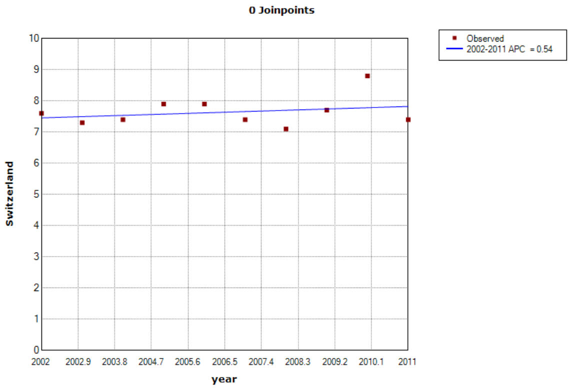

Southern Europe

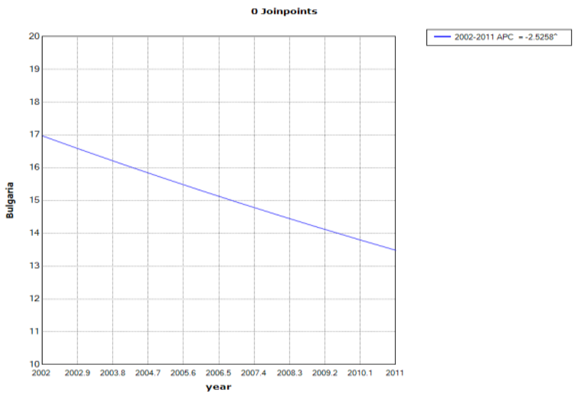

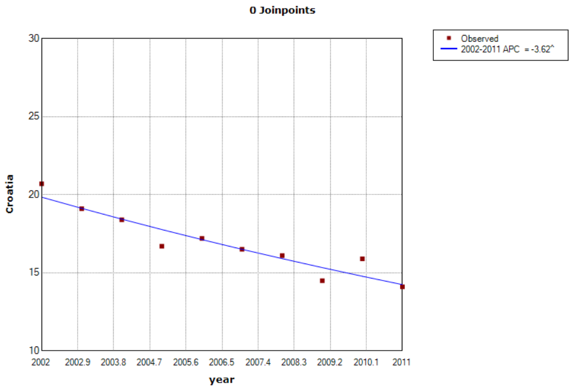

© 2021 Wong MCS et al. *JAMA Network Open.*

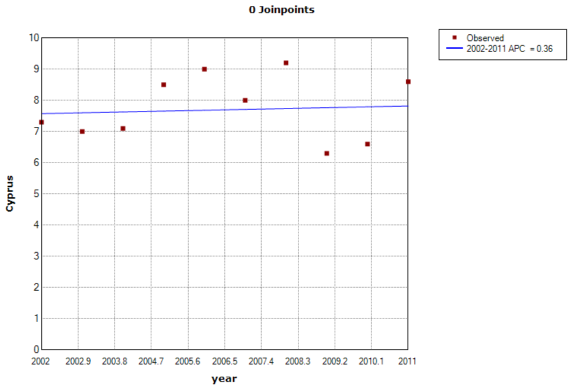

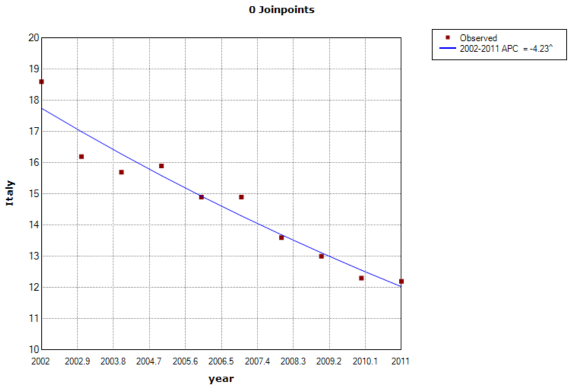

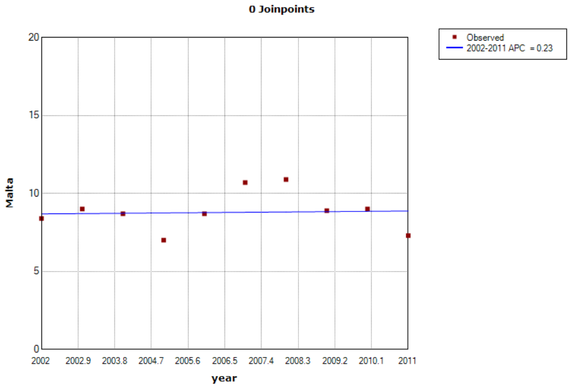

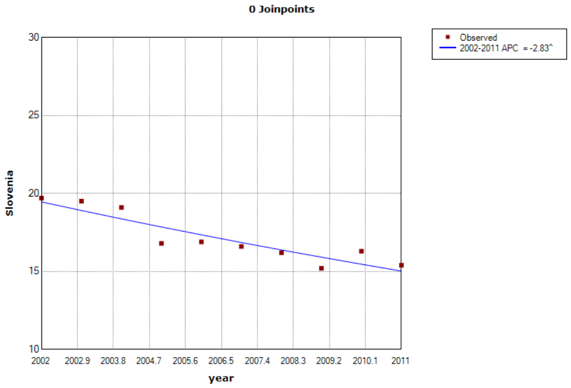

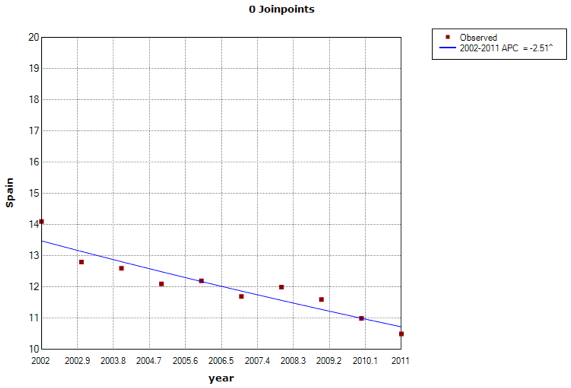

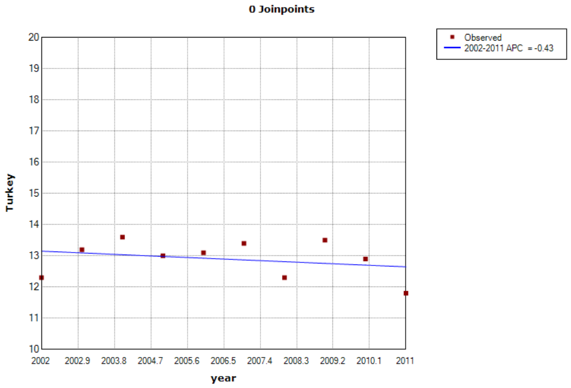

© 2021 Wong MCS et al. *JAMA Network Open.*

Eastern Europe

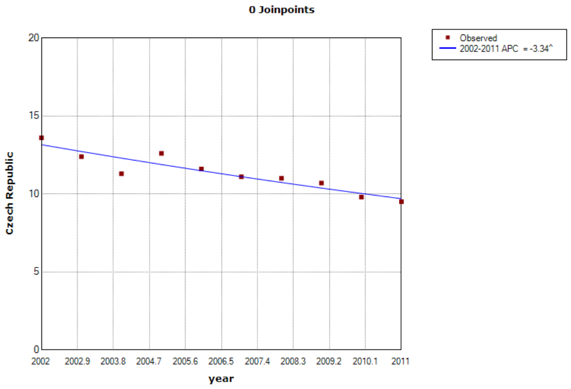

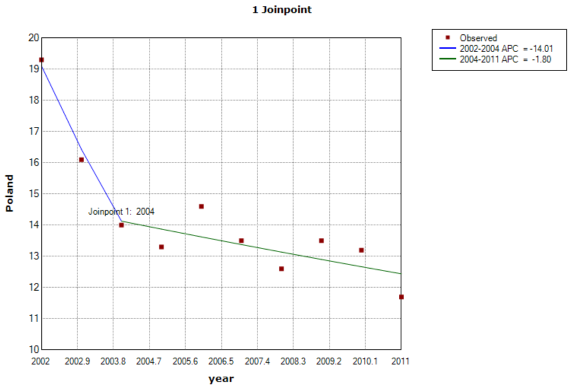

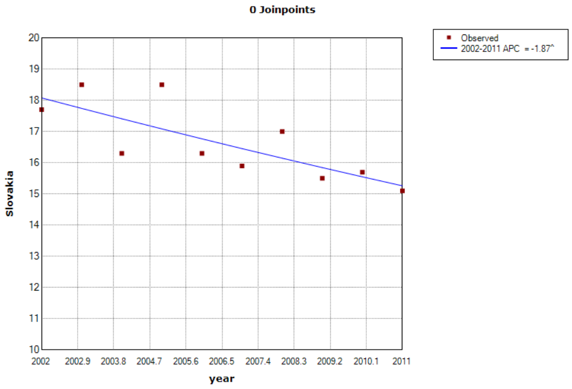

Africa

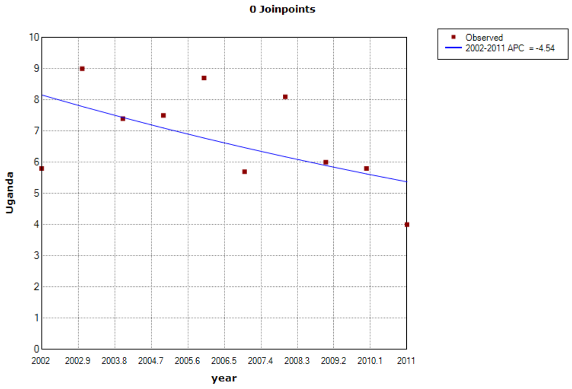

© 2021 Wong MCS et al. *JAMA Network Open.*

b.) Incidence female all ages

Asia

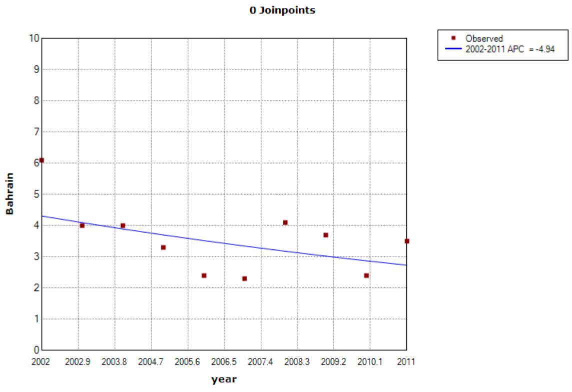

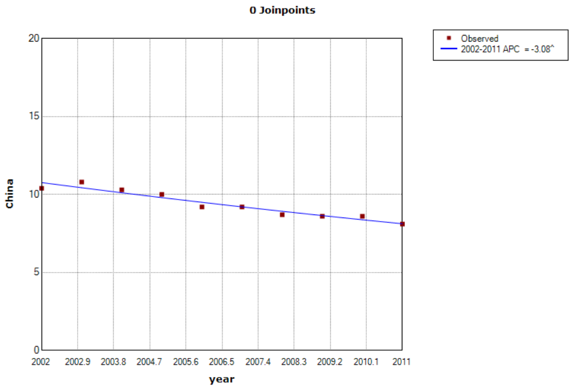

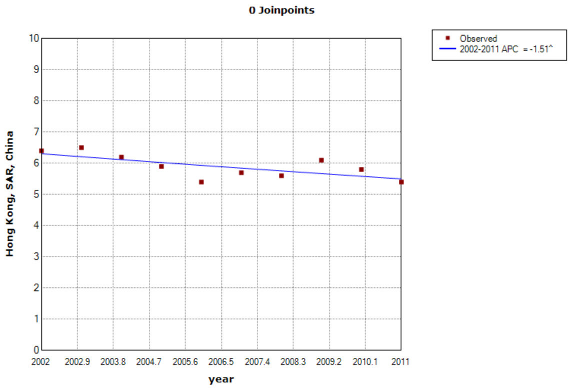

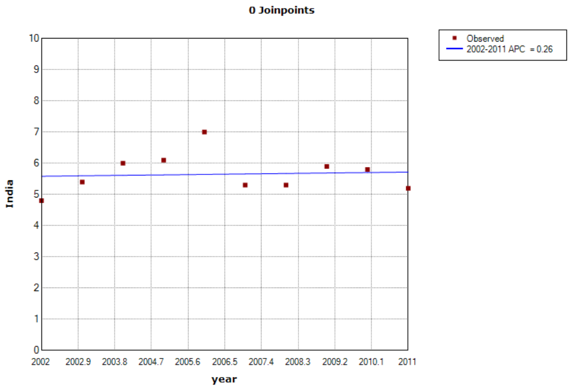

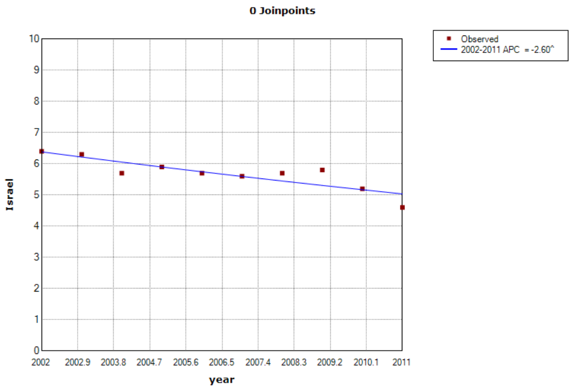

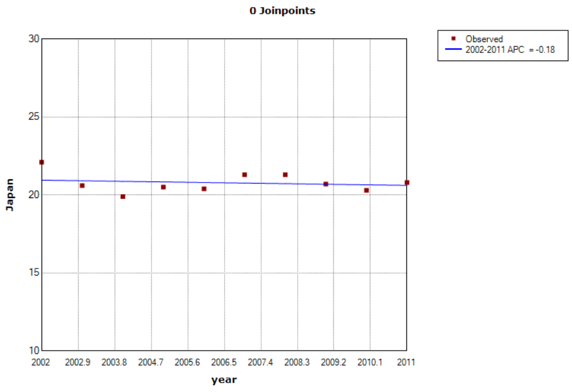

© 2021 Wong MCS et al. *JAMA Network Open.*

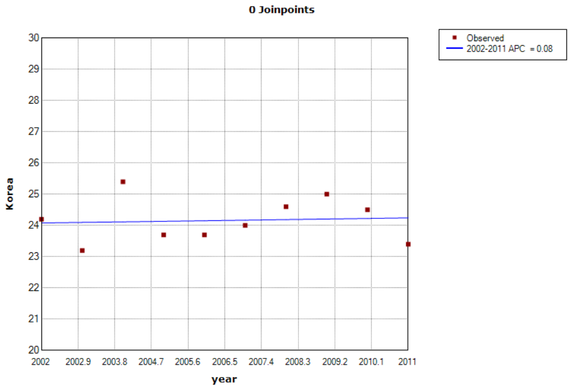

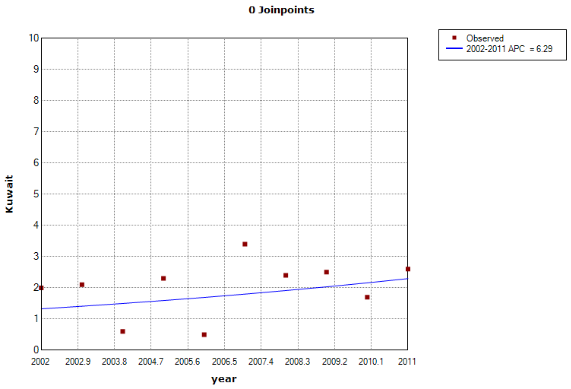

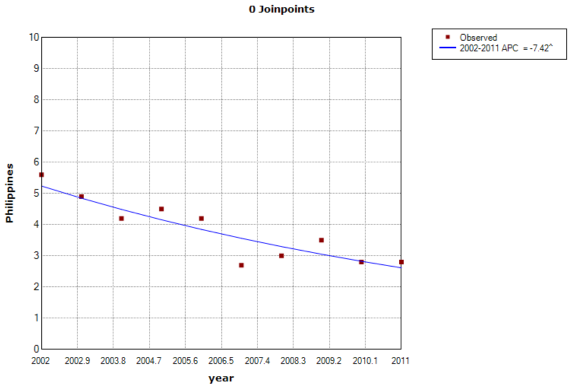

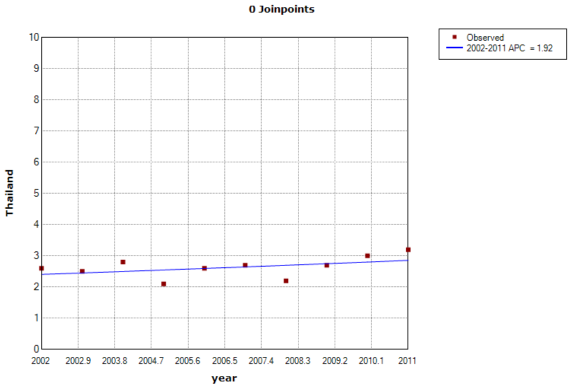

Oceania

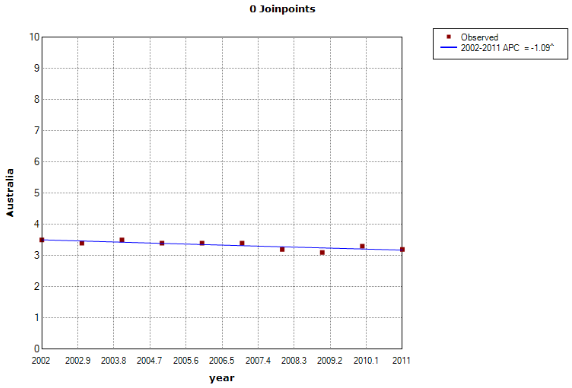

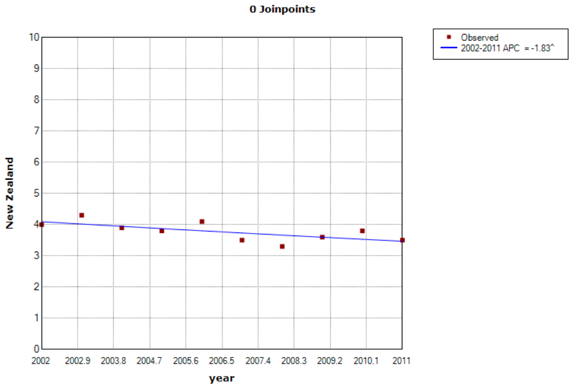

© 2021 Wong MCS et al. *JAMA Network Open.*

Northern America

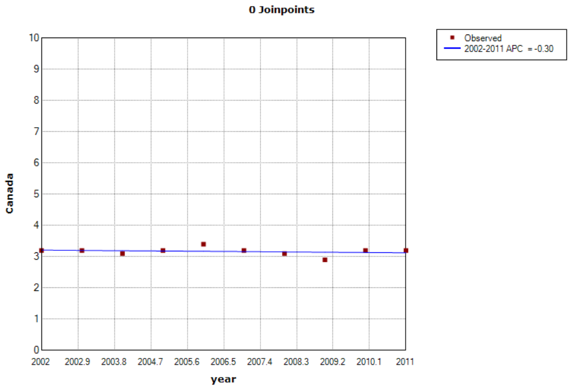

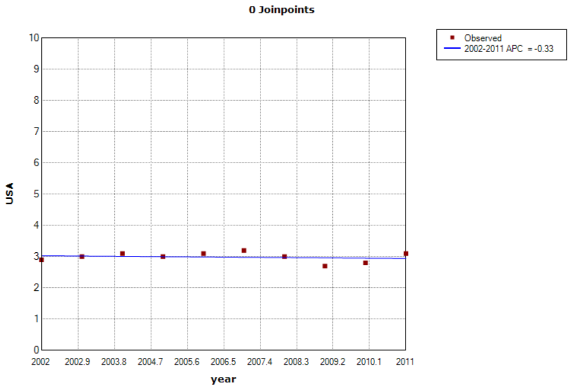

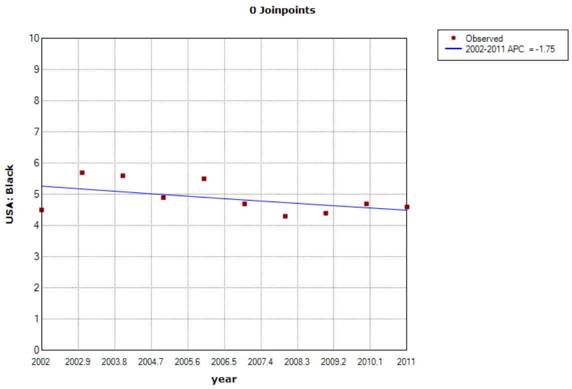

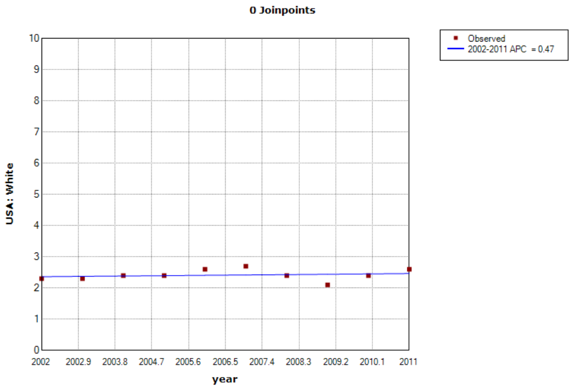

Southern America

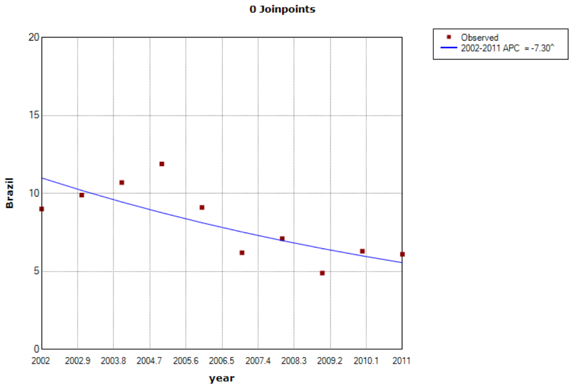

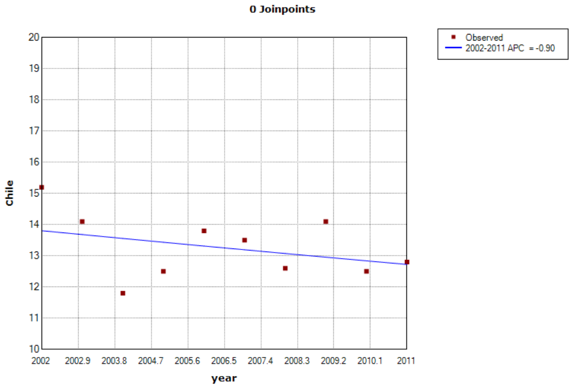

© 2021 Wong MCS et al. *JAMA Network Open.*

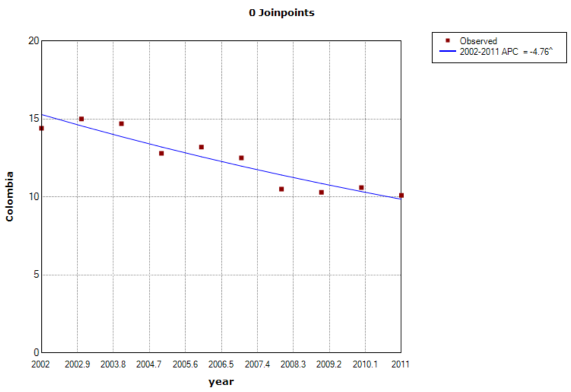

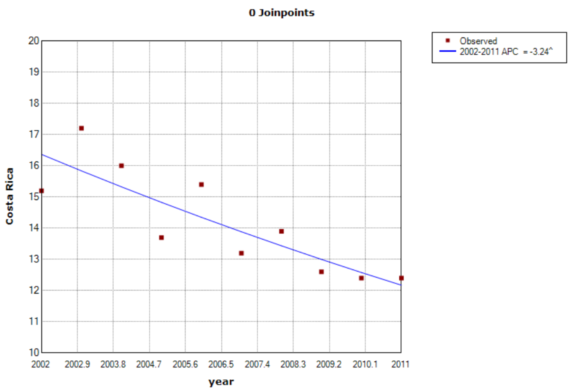

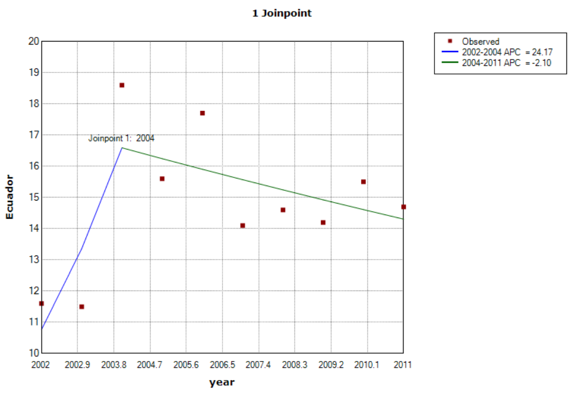

Northern Europe

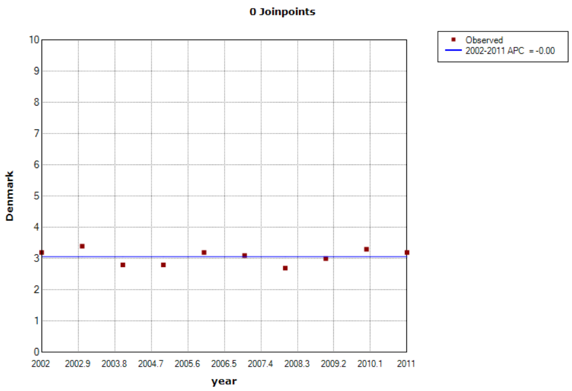

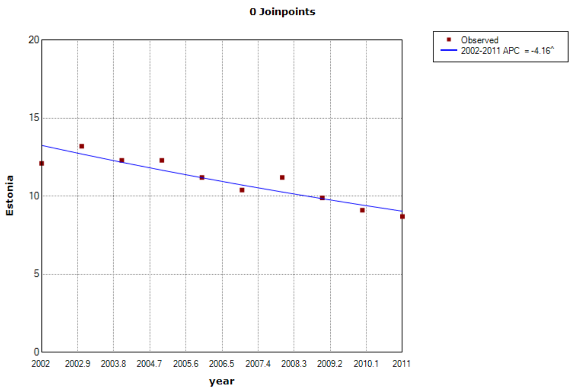

© 2021 Wong MCS et al. *JAMA Network Open.*

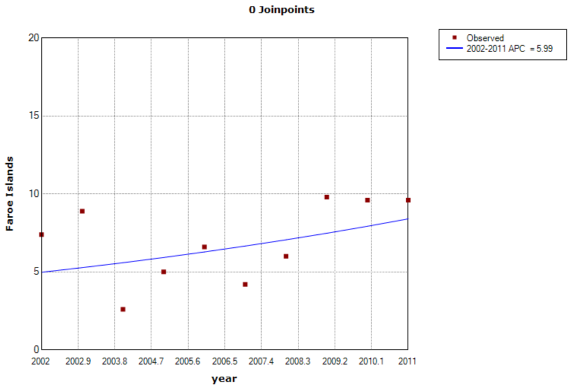

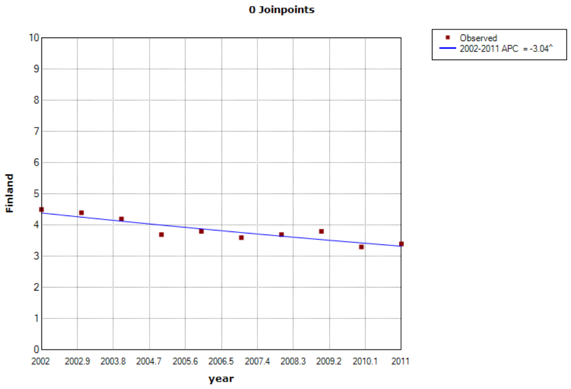

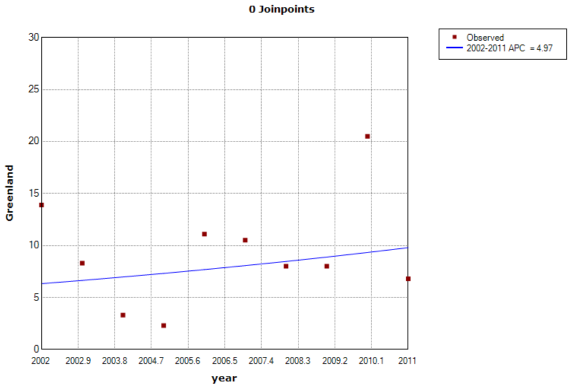

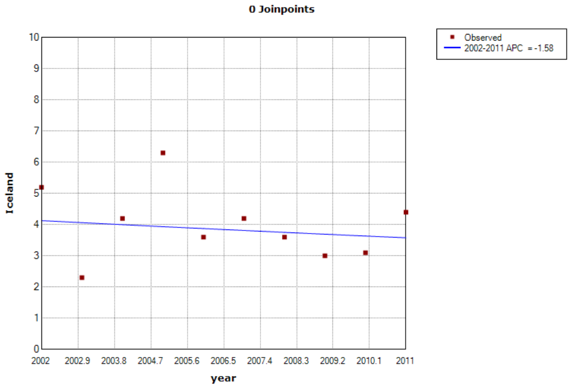

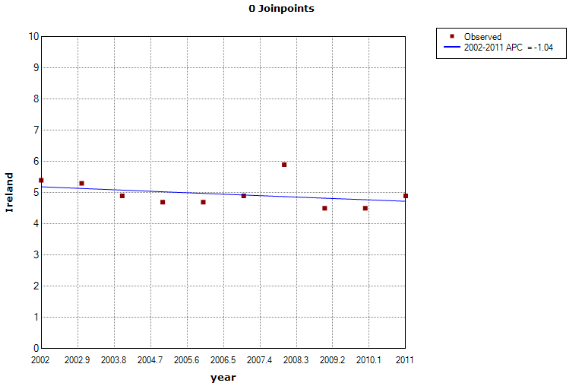

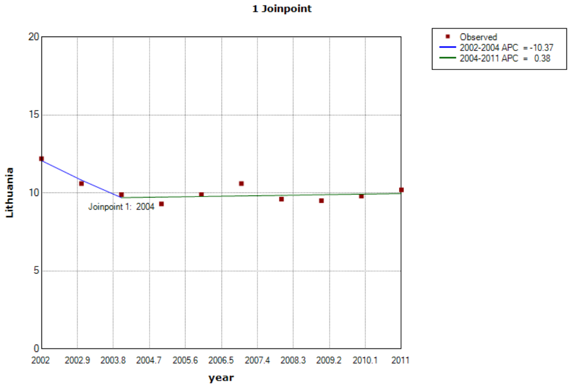

© 2021 Wong MCS et al. *JAMA Network Open.*

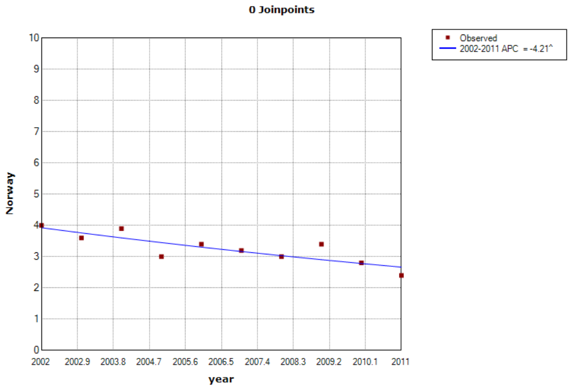

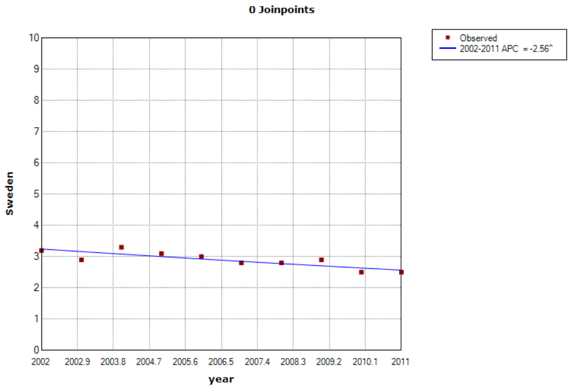

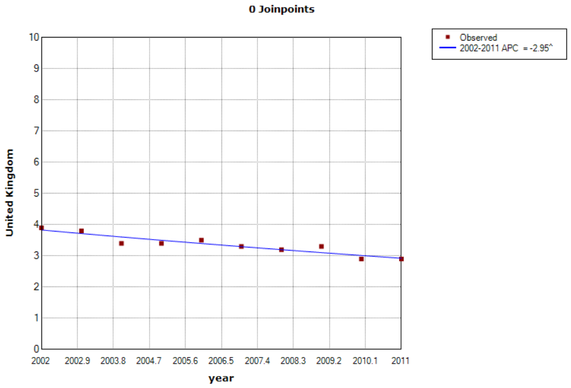

Western Europe

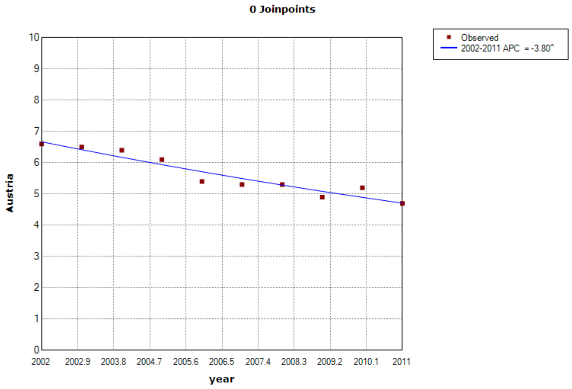

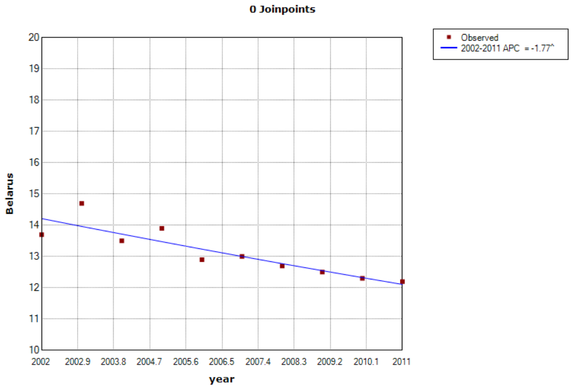

© 2021 Wong MCS et al. *JAMA Network Open.*

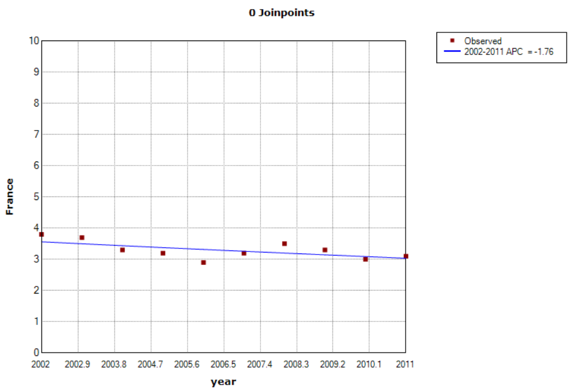

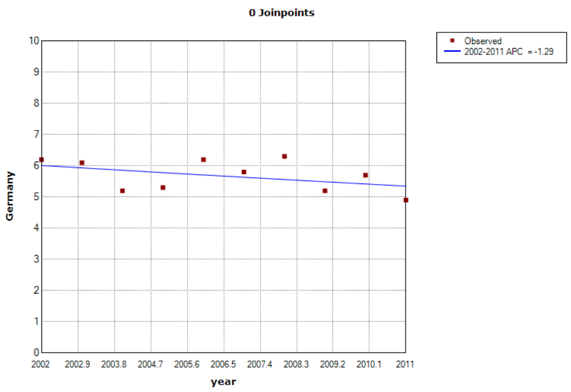

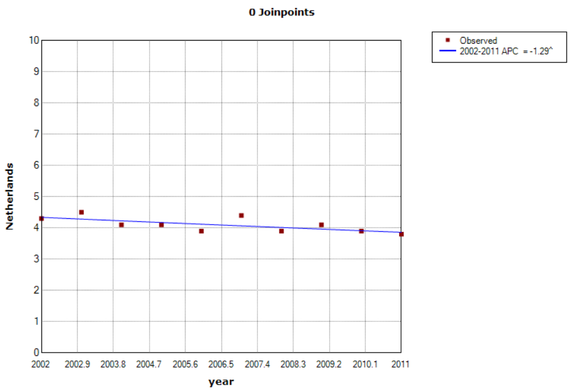

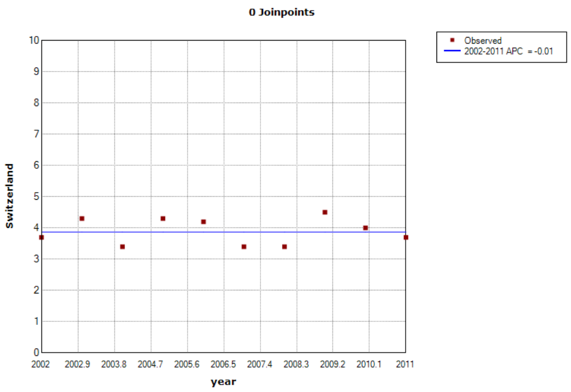

Southern Europe

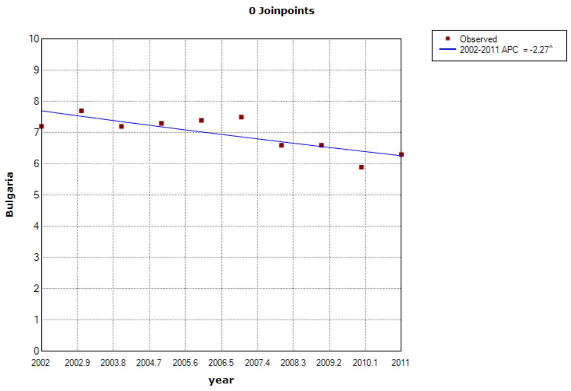

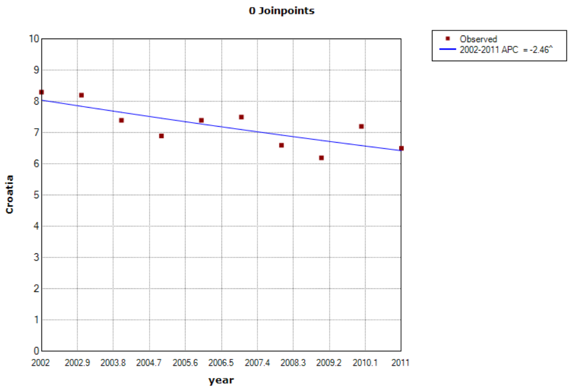

© 2021 Wong MCS et al. *JAMA Network Open.*

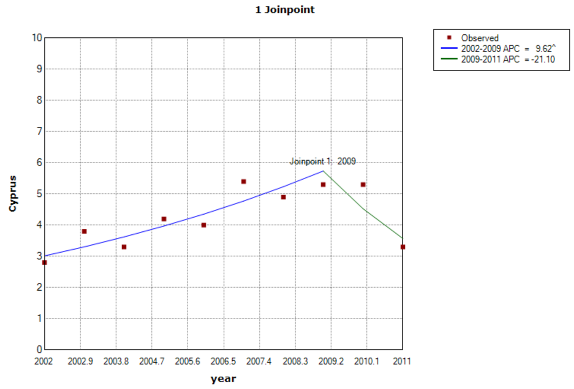

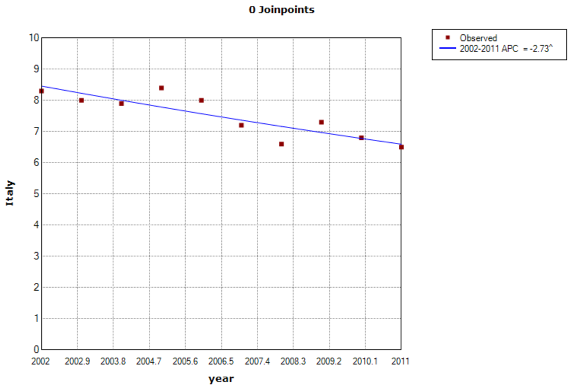

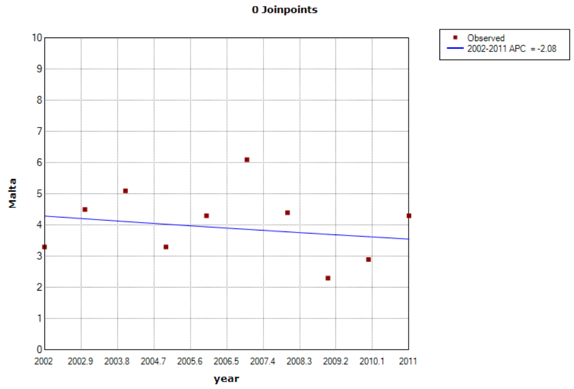

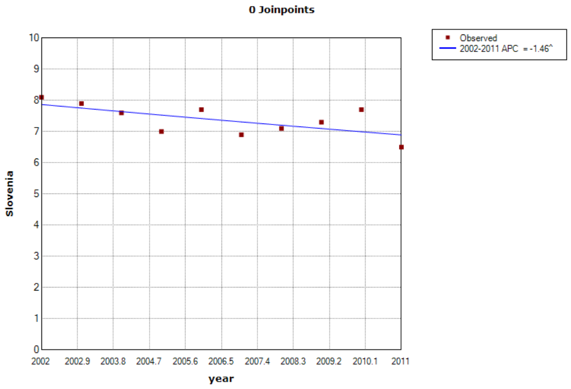

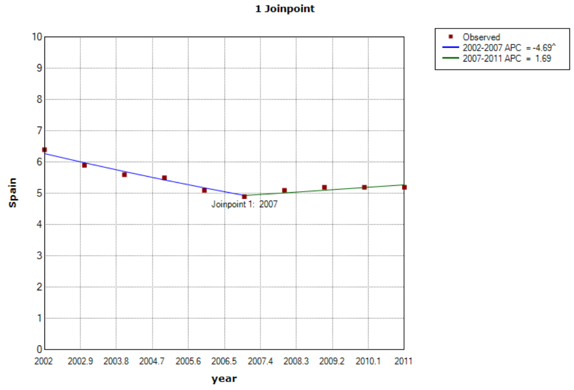

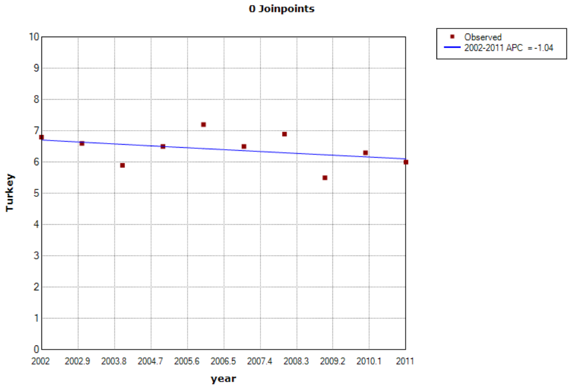

© 2021 Wong MCS et al. *JAMA Network Open.*

Eastern Europe

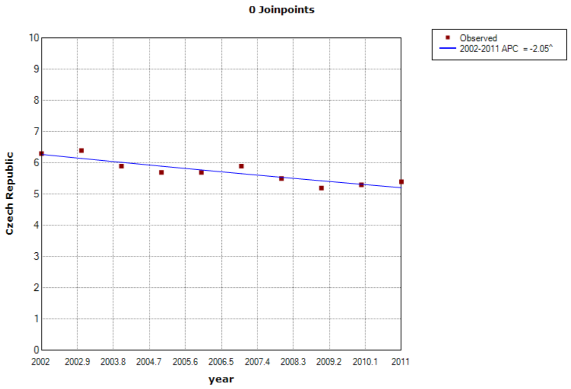

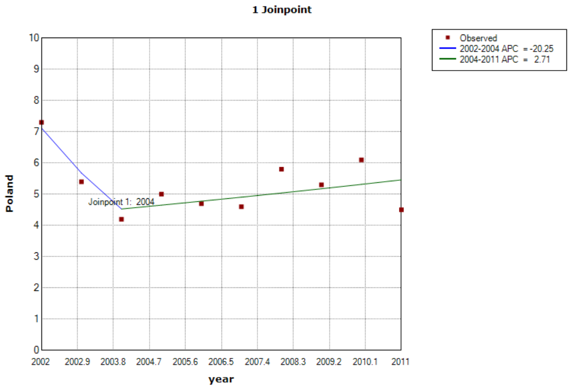

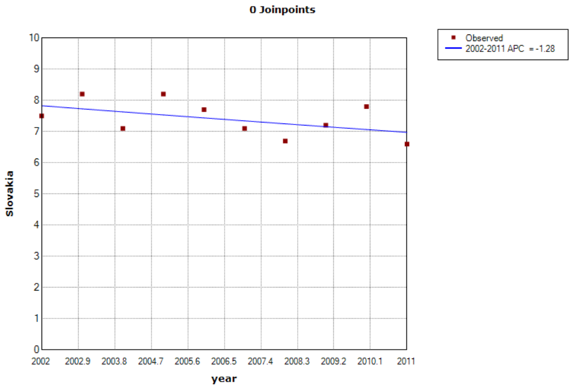

Africa

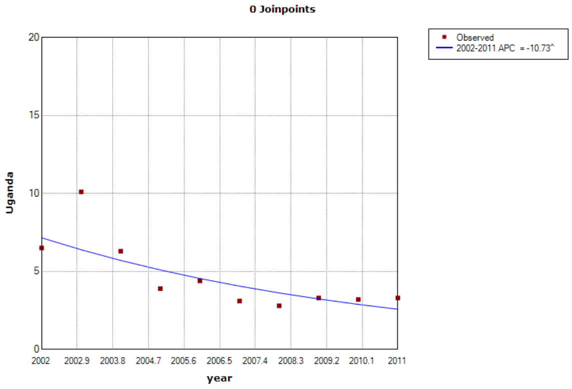

© 2021 Wong MCS et al. *JAMA Network Open.*

c.) Incidence male below 40 years old

Asia

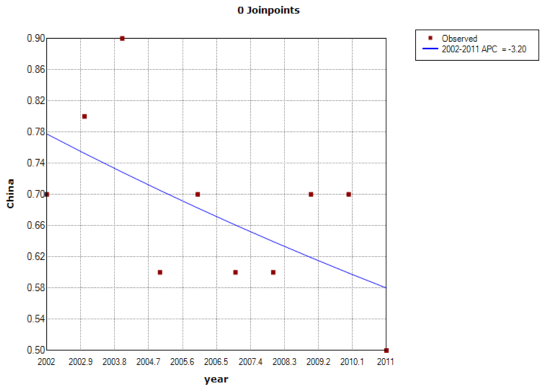

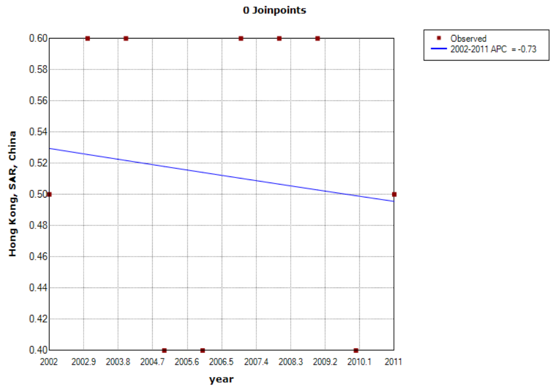

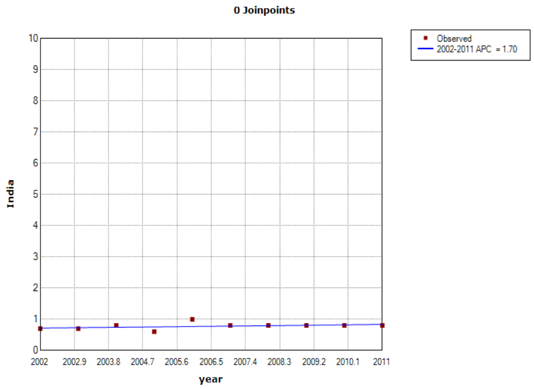

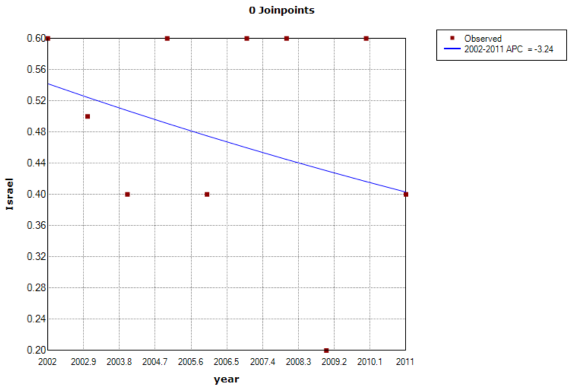

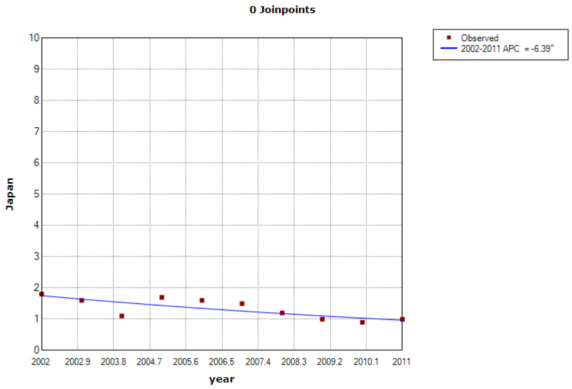

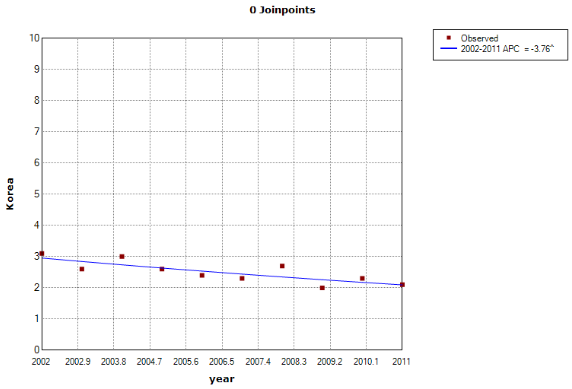

© 2021 Wong MCS et al. *JAMA Network Open.*

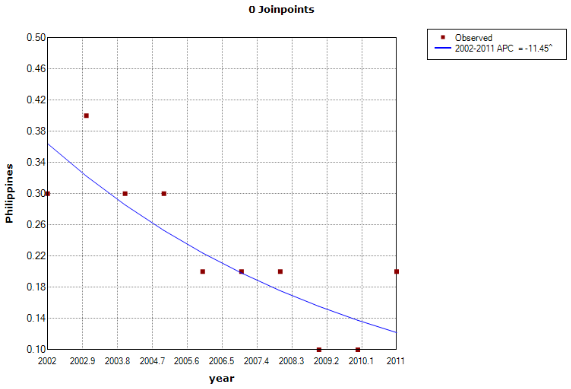

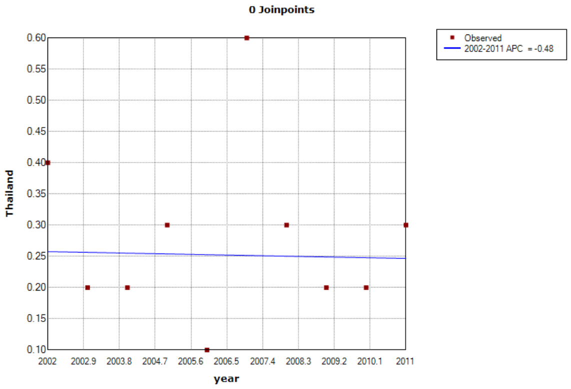

Oceania

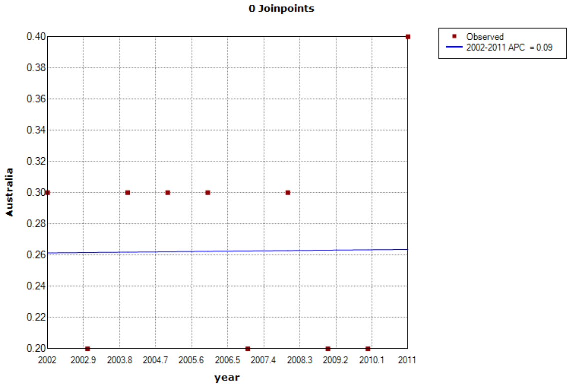

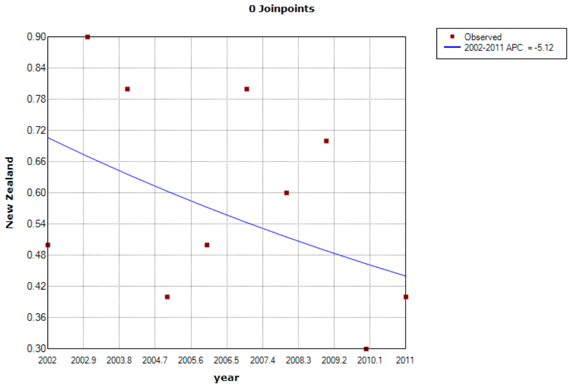

Northern America

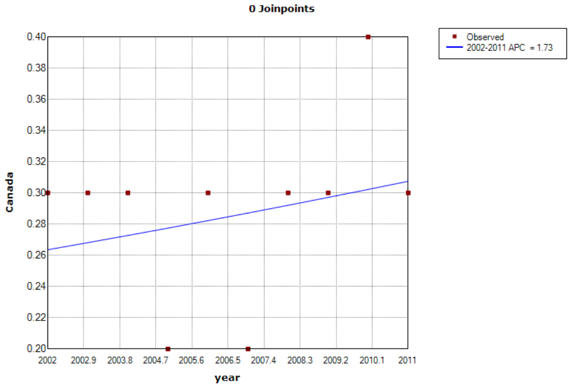

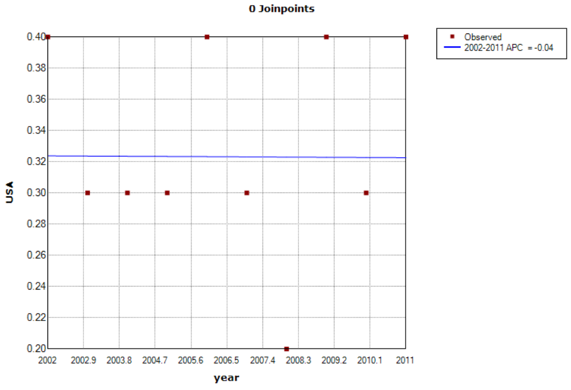

© 2021 Wong MCS et al. *JAMA Network Open.*

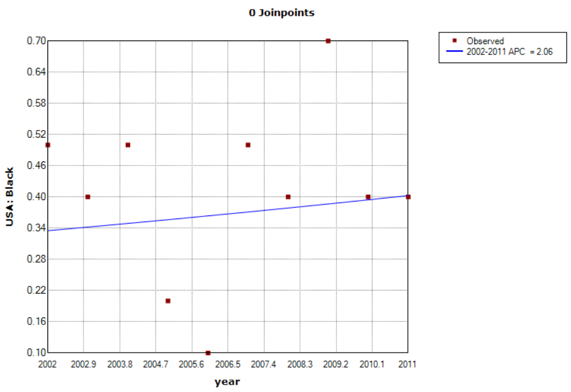

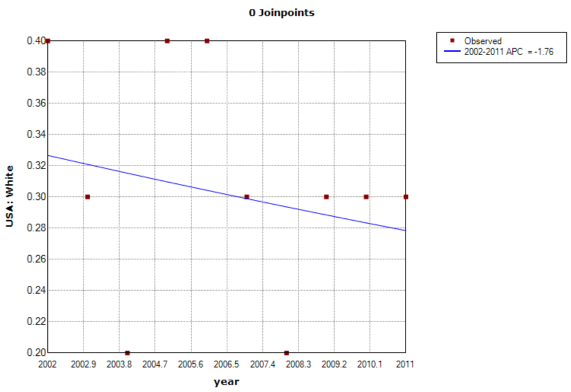

Southern America

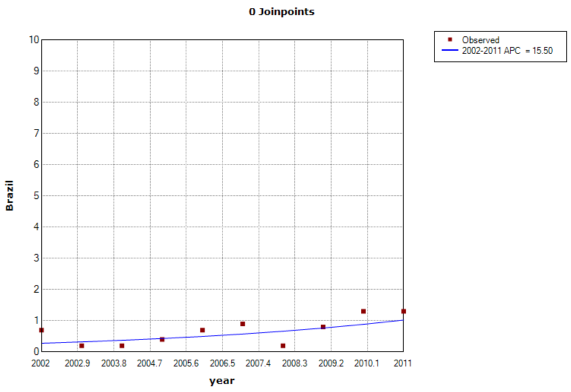

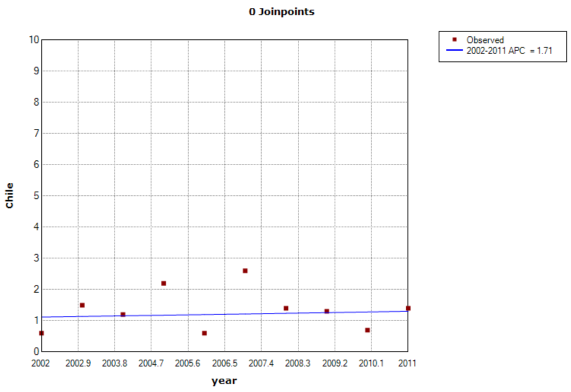

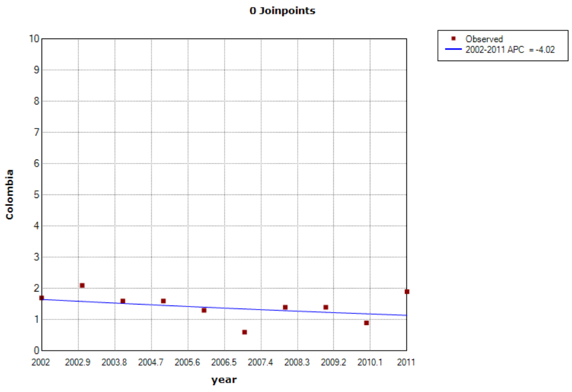

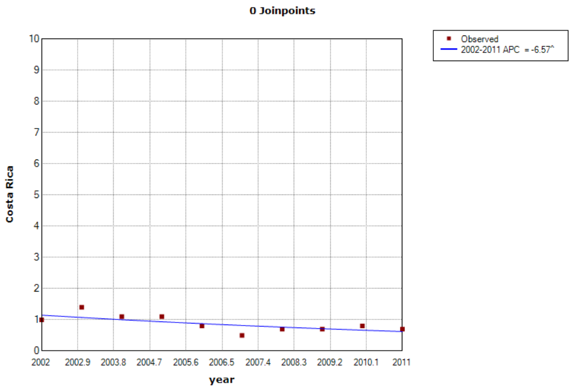

© 2021 Wong MCS et al. *JAMA Network Open.*

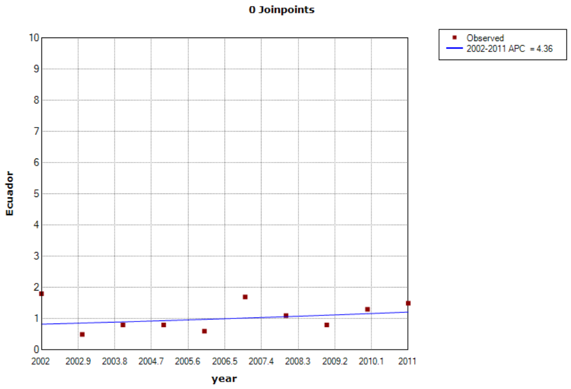

Northern Europe

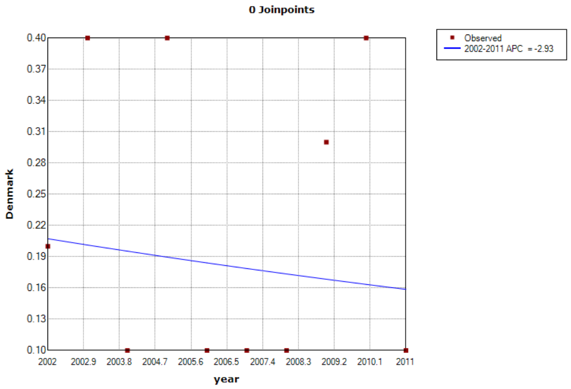

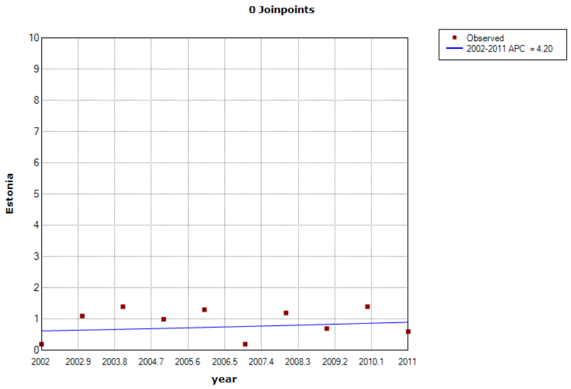

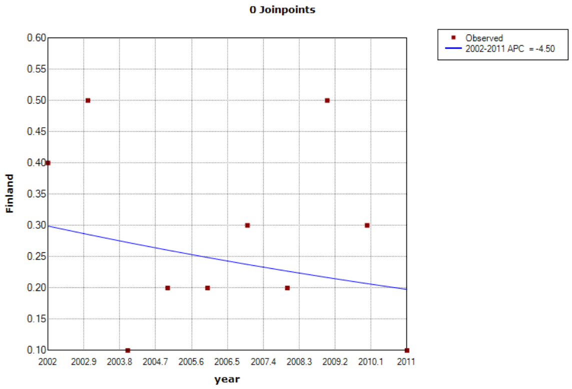

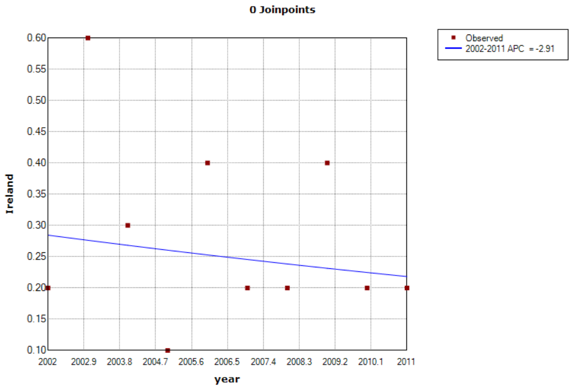

© 2021 Wong MCS et al. *JAMA Network Open.*

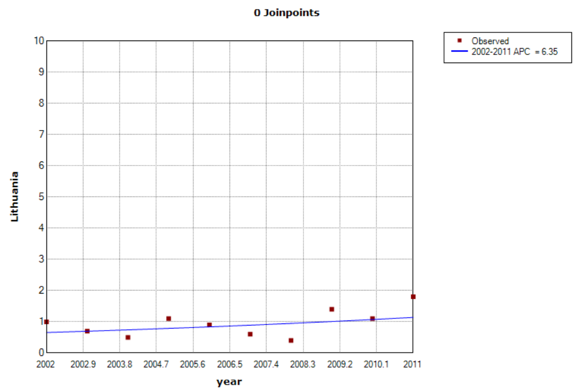

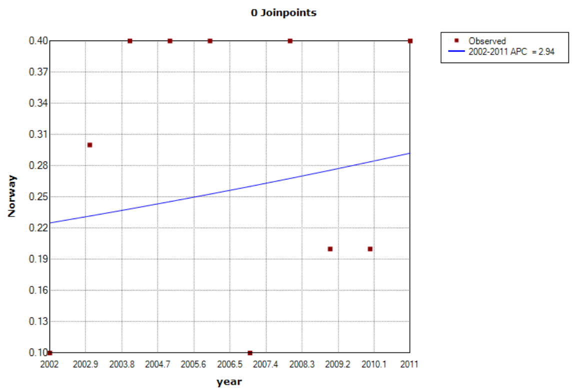

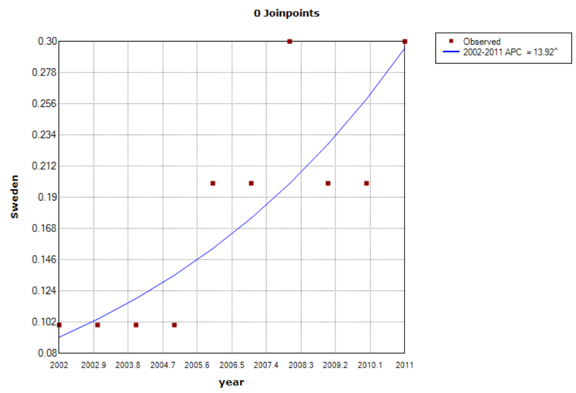

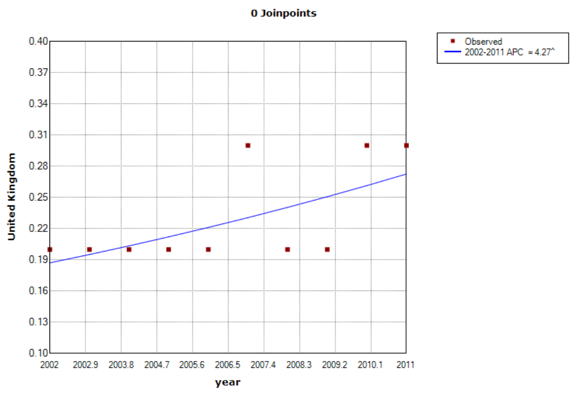

Western Europe

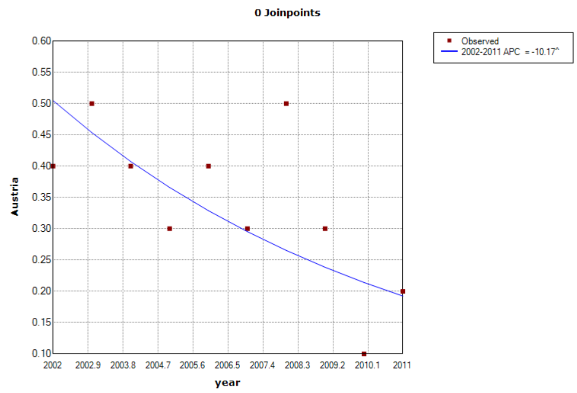

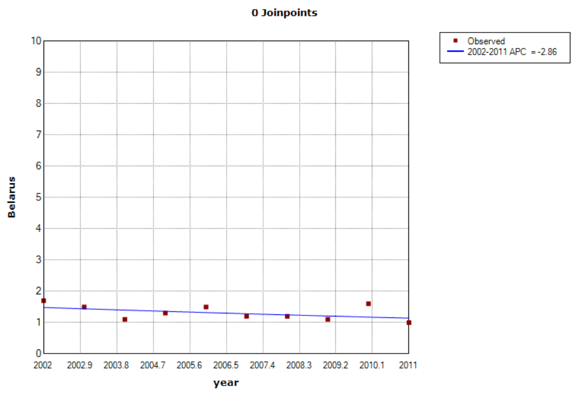

© 2021 Wong MCS et al. *JAMA Network Open.*

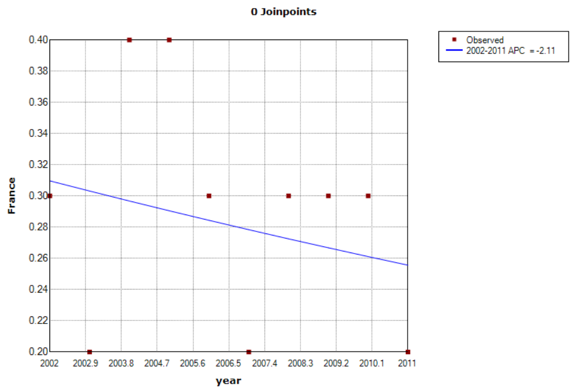

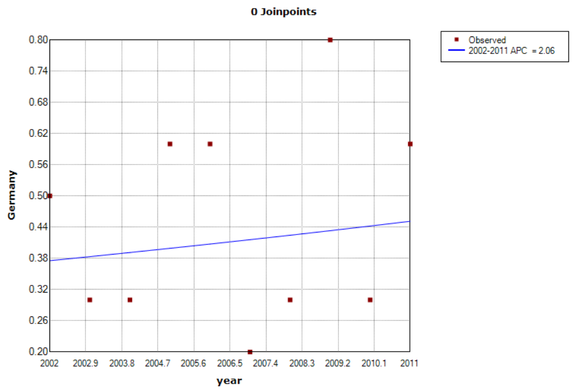

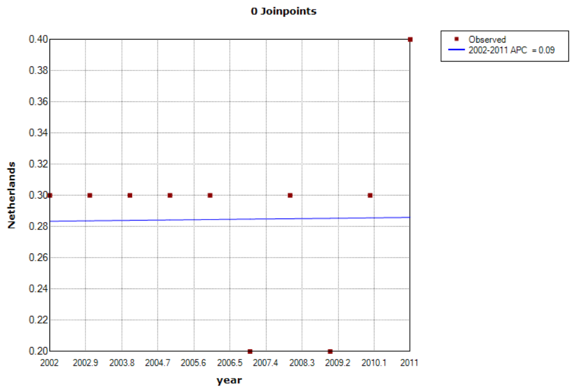

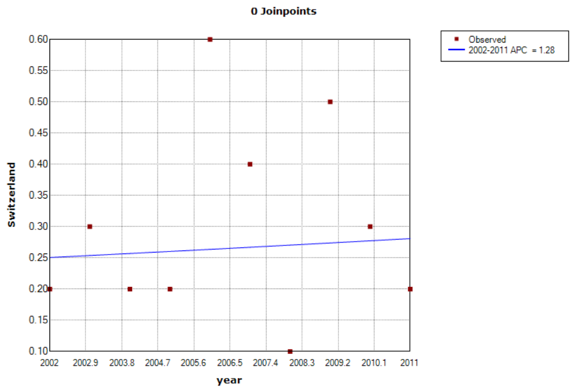

Southern Europe

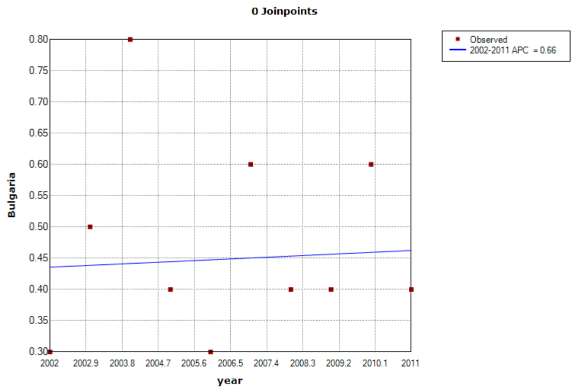

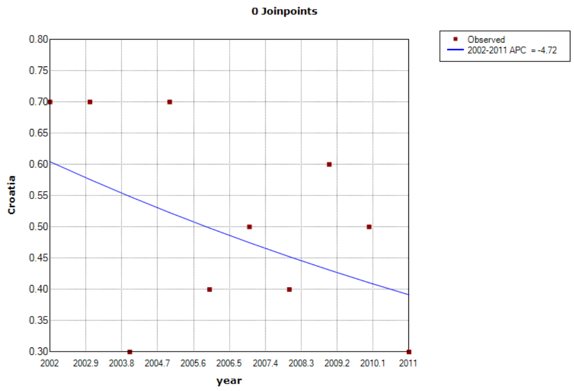

© 2021 Wong MCS et al. *JAMA Network Open.*

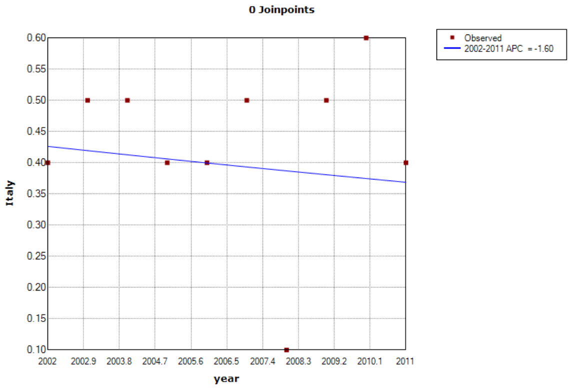

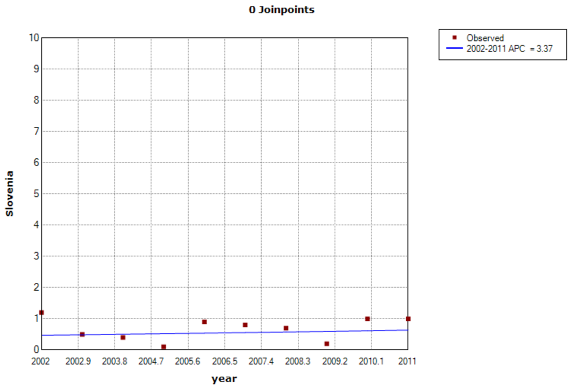

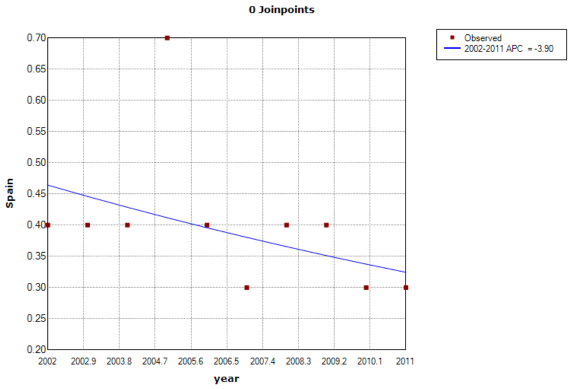

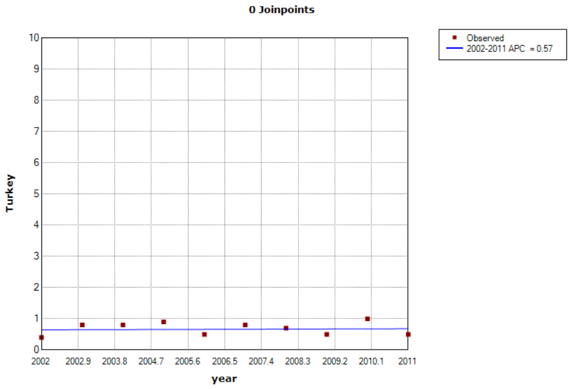

Eastern Europe

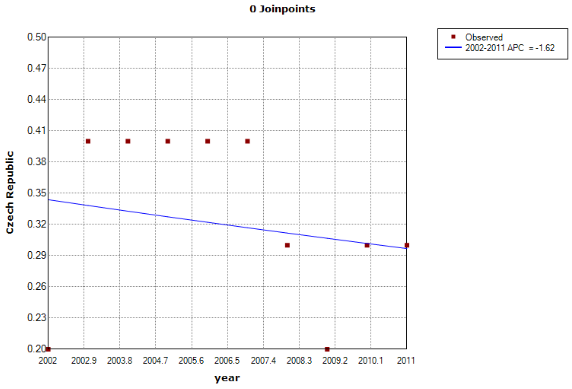

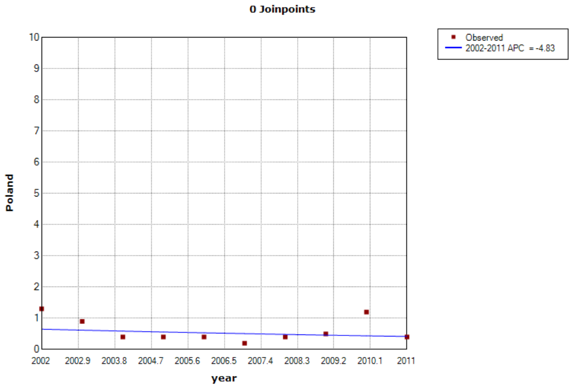

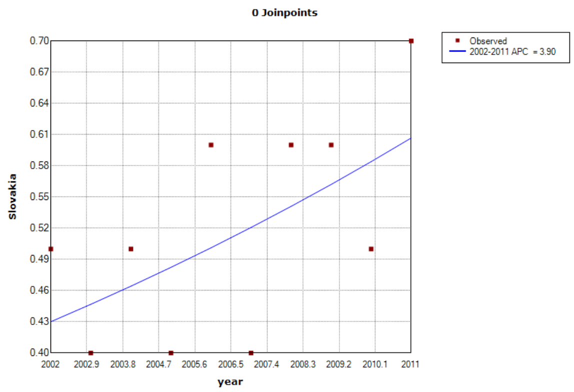

Africa

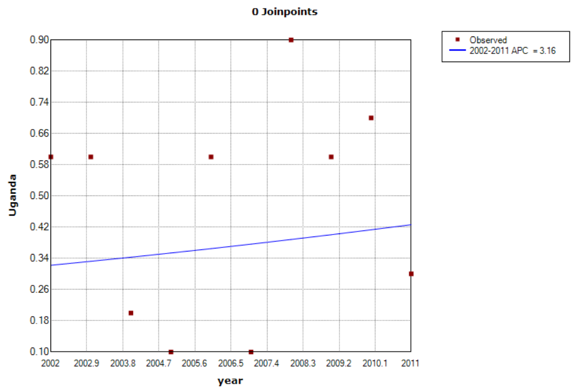

© 2021 Wong MCS et al. *JAMA Network Open.*

d.) Incidence female below 40 years old

Asia

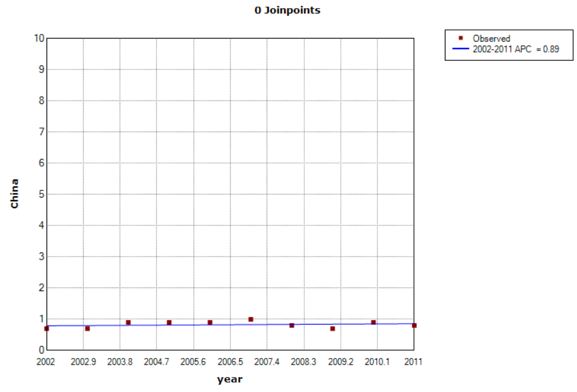

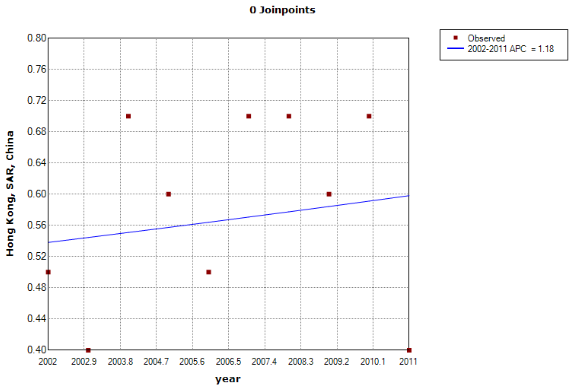

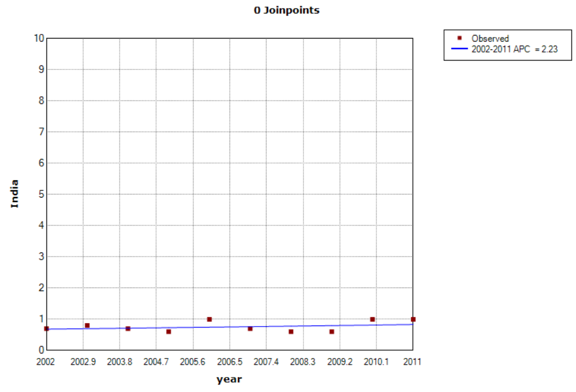

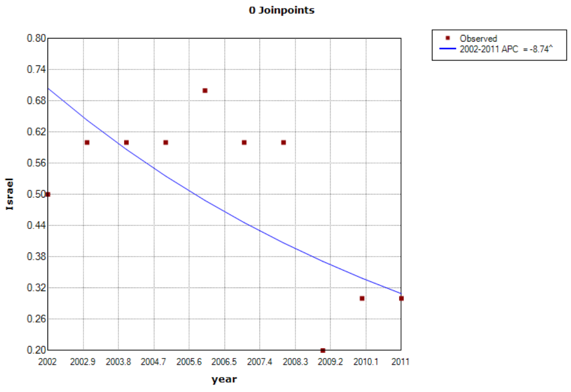

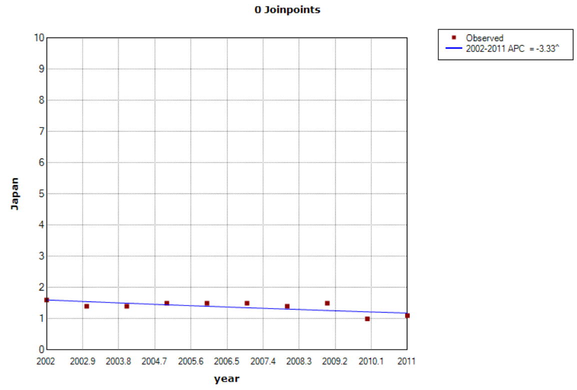

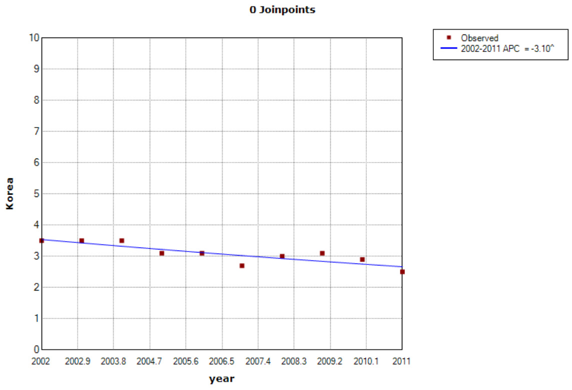

© 2021 Wong MCS et al. *JAMA Network Open.*

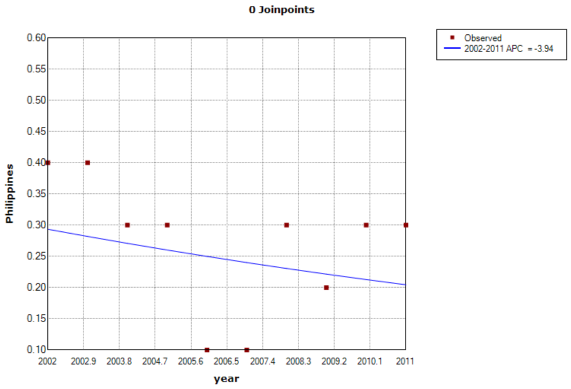

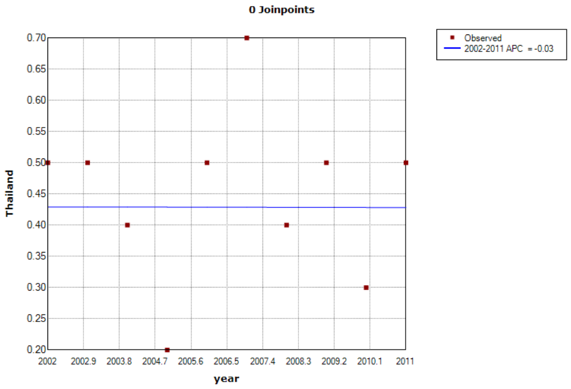

Oceania

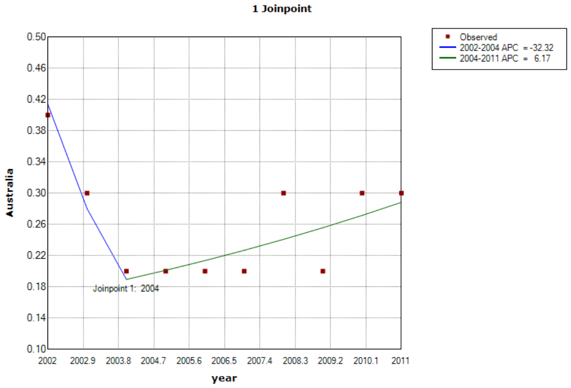

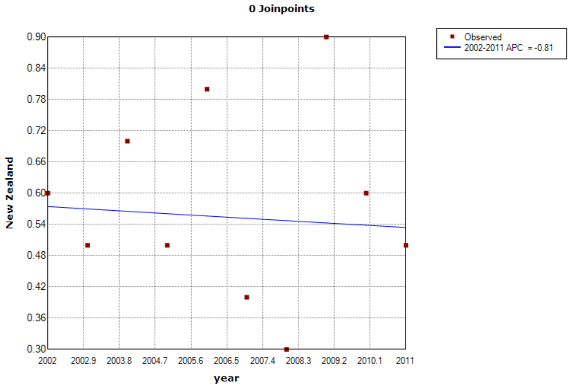

Northern America

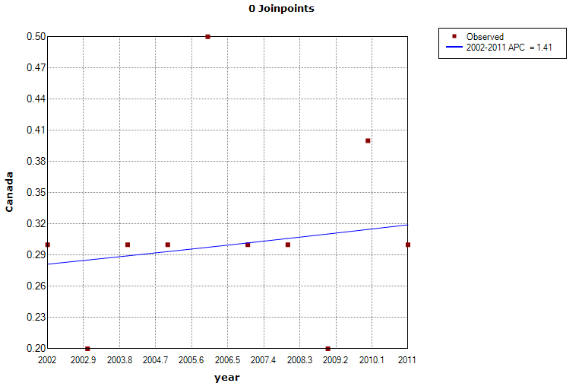

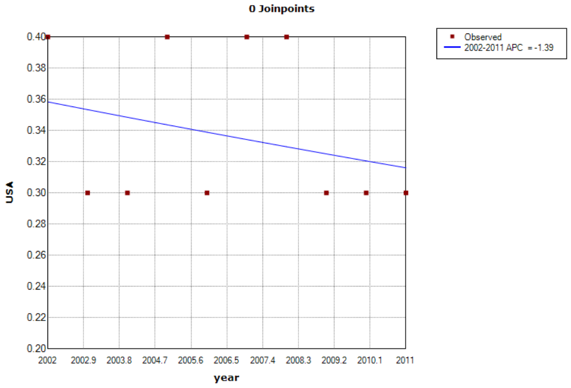

© 2021 Wong MCS et al. *JAMA Network Open.*

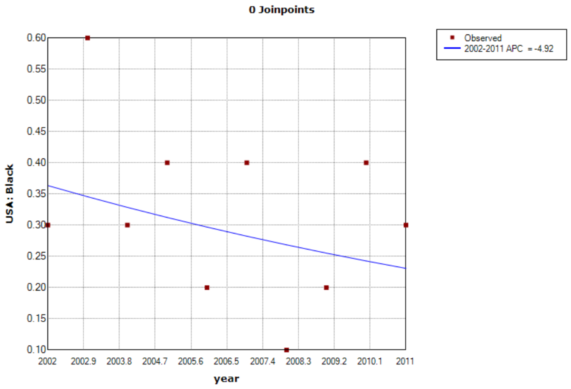

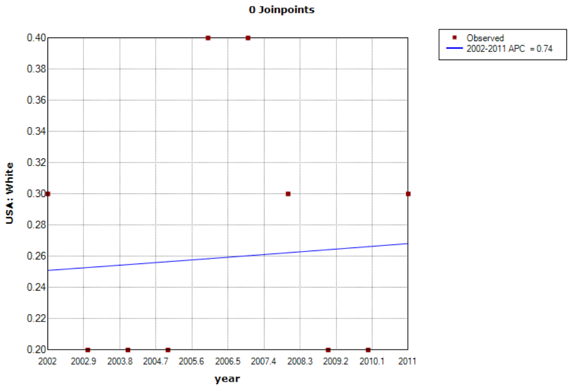

Southern America

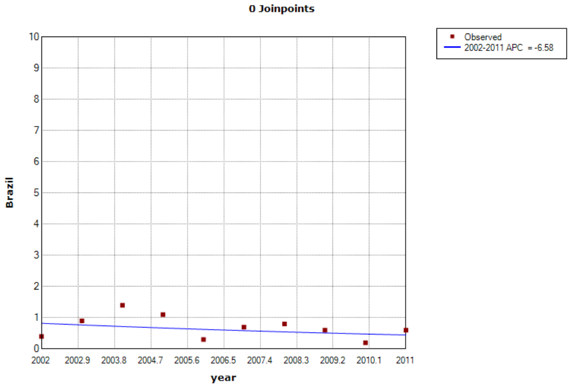

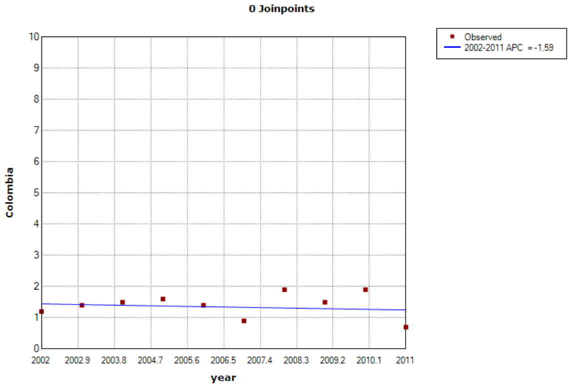

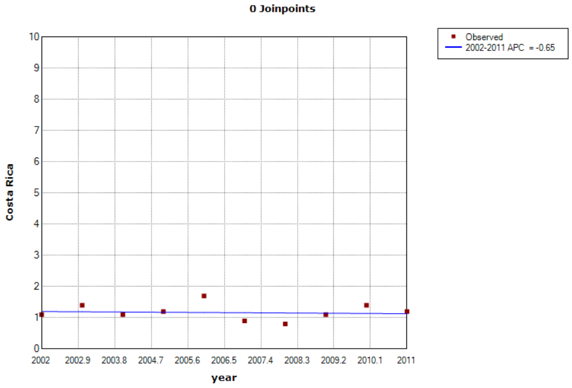

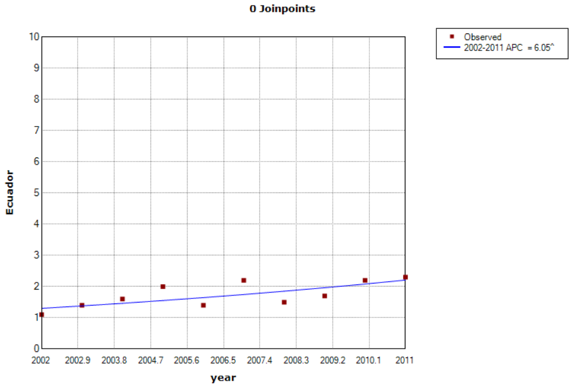

© 2021 Wong MCS et al. *JAMA Network Open.*

Northern Europe

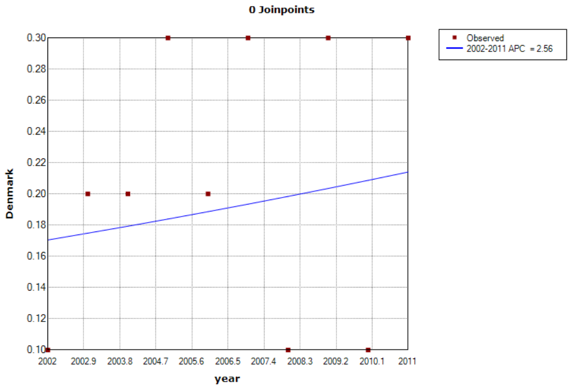

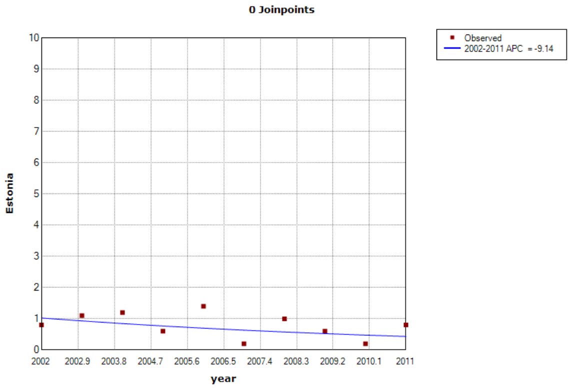

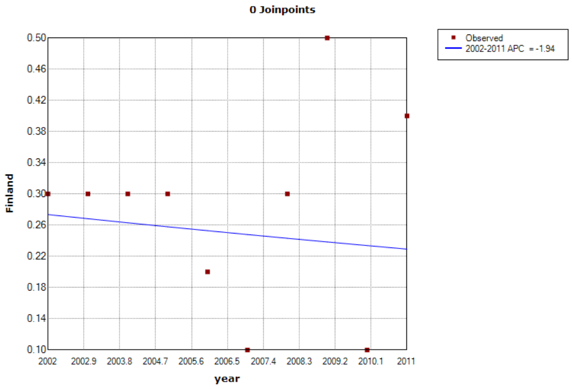

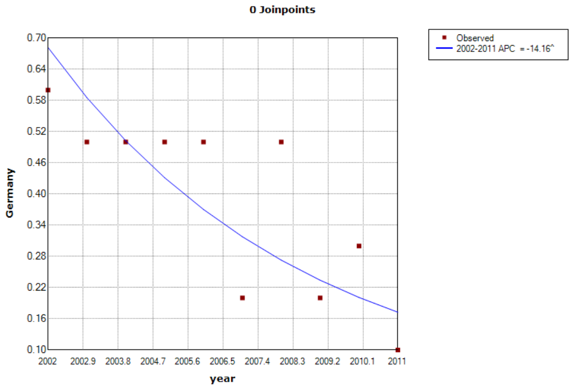

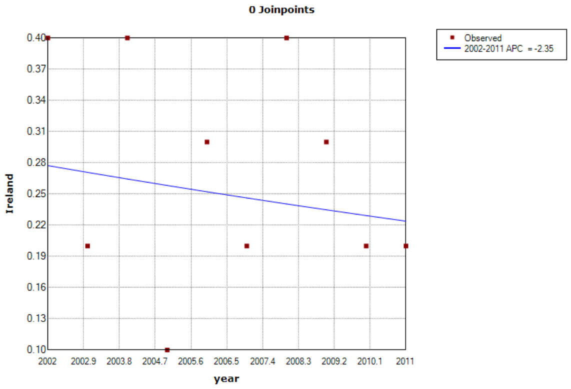

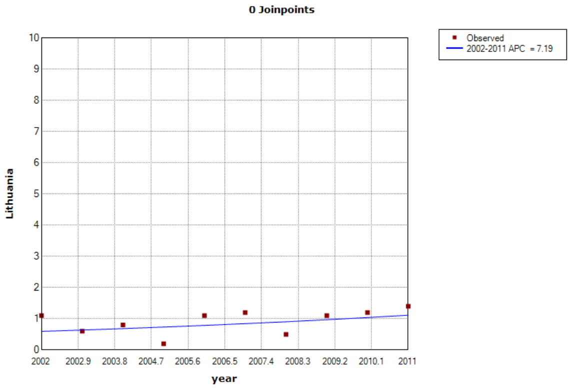

© 2021 Wong MCS et al. *JAMA Network Open.*

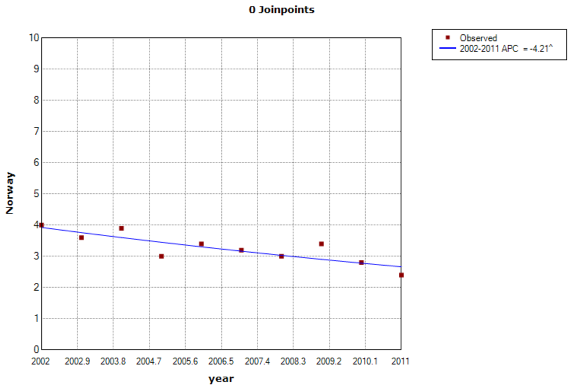

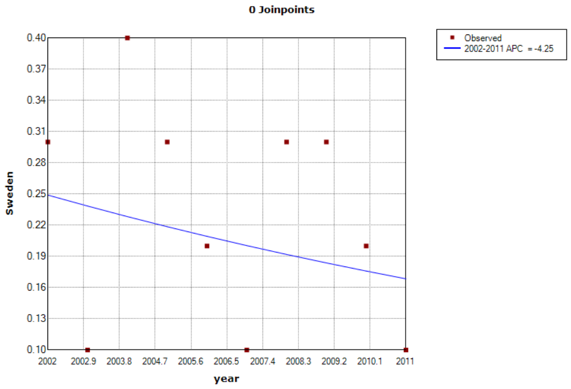

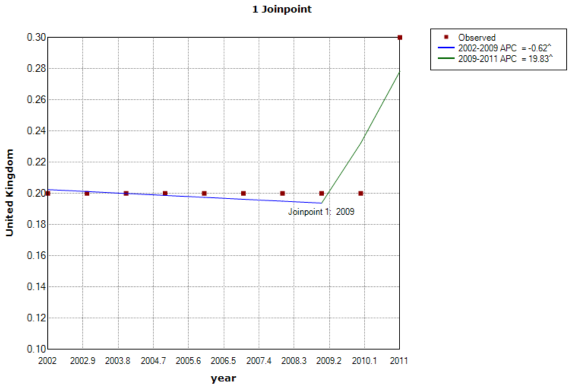

Western Europe

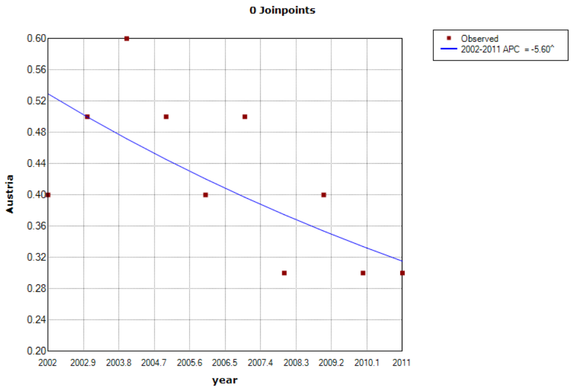

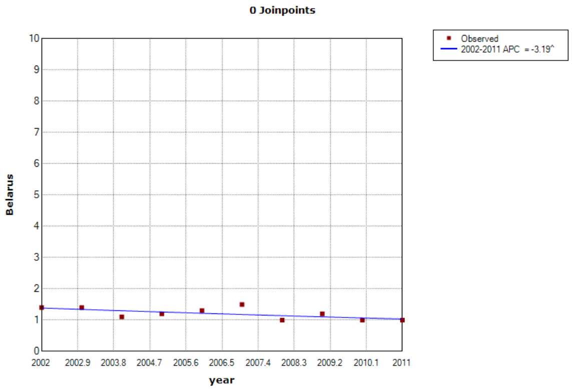

© 2021 Wong MCS et al. *JAMA Network Open.*

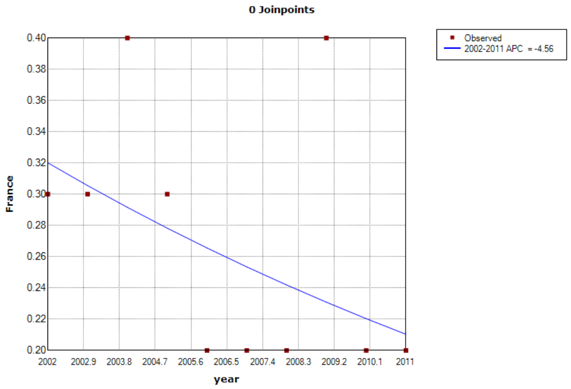

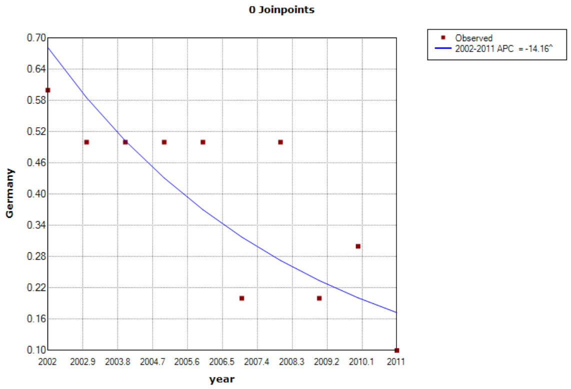

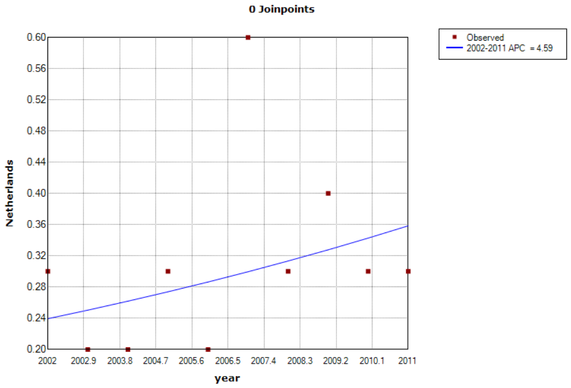

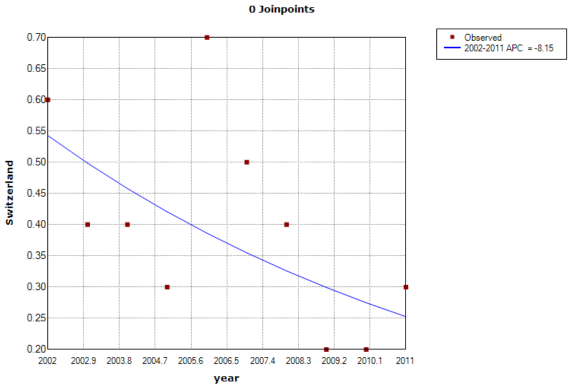

Southern Europe

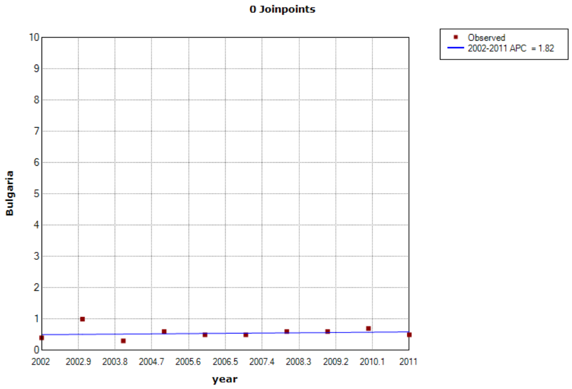

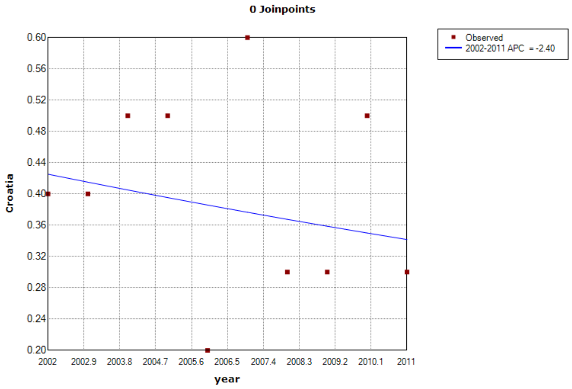

© 2021 Wong MCS et al. *JAMA Network Open.*

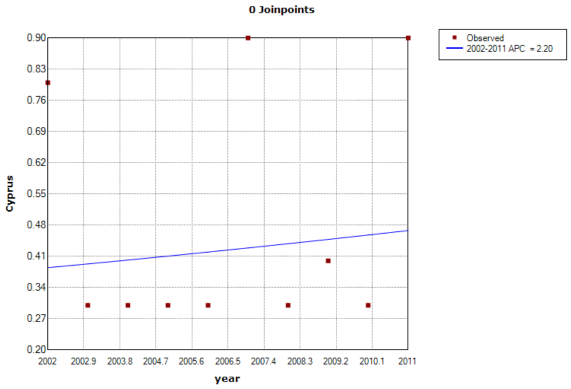

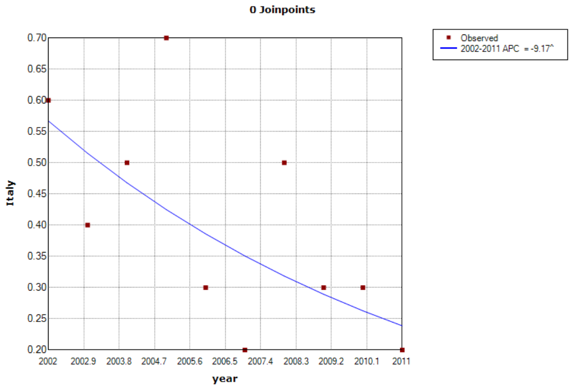

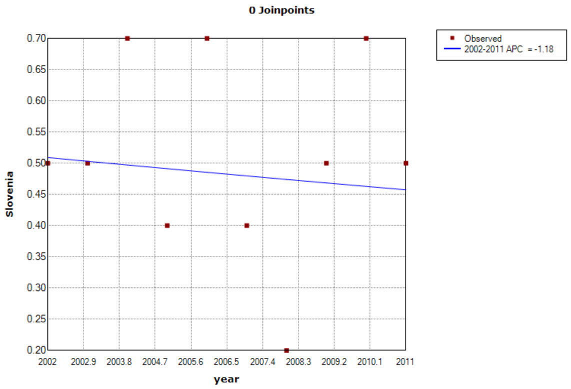

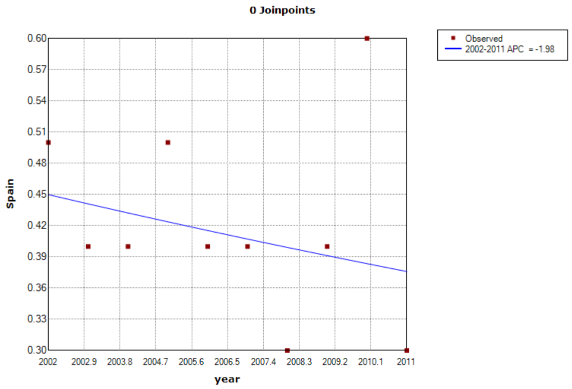

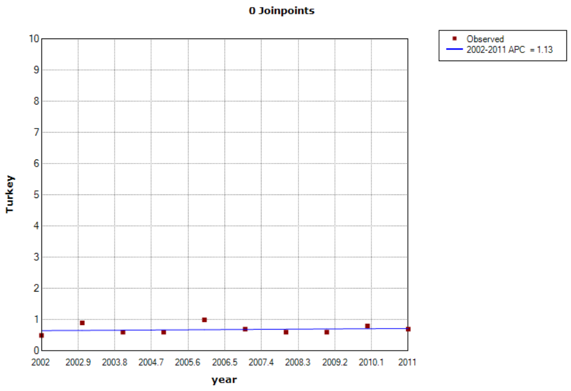

© 2021 Wong MCS et al. *JAMA Network Open.*

Eastern Europe

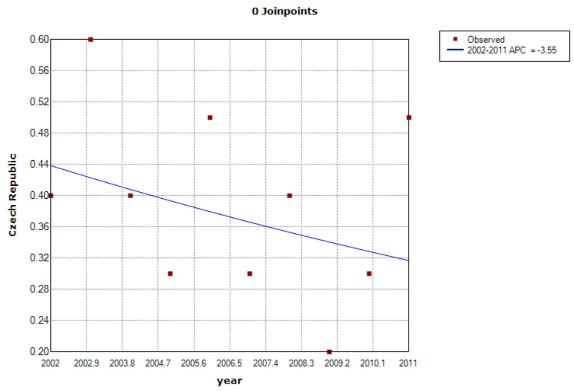

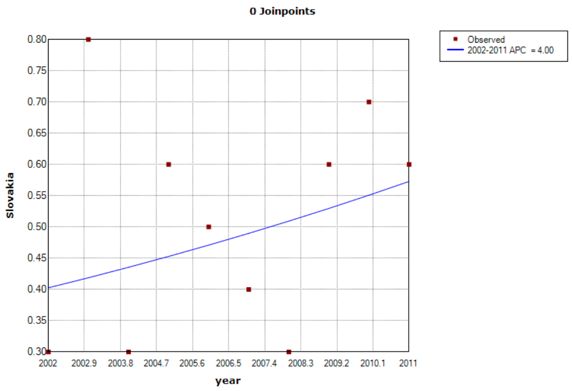

© 2021 Wong MCS et al. *JAMA Network Open.*

e.) Incidence male above 40 years old

Asia

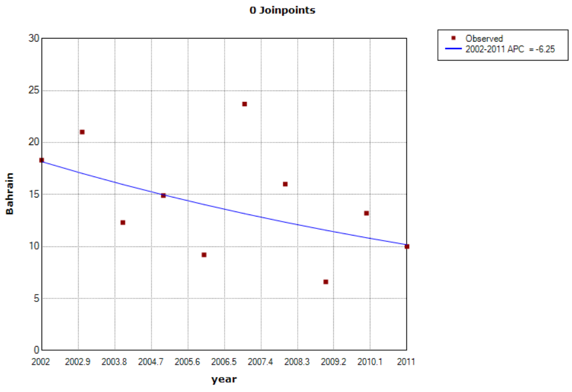

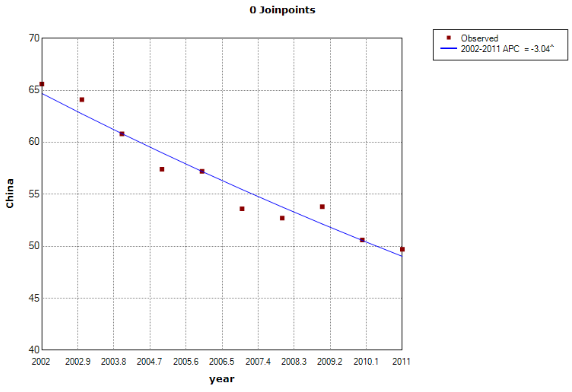

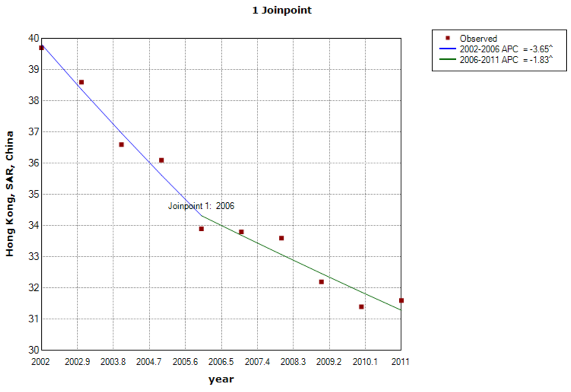

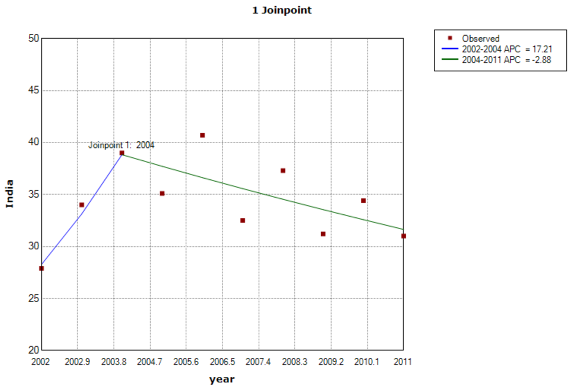

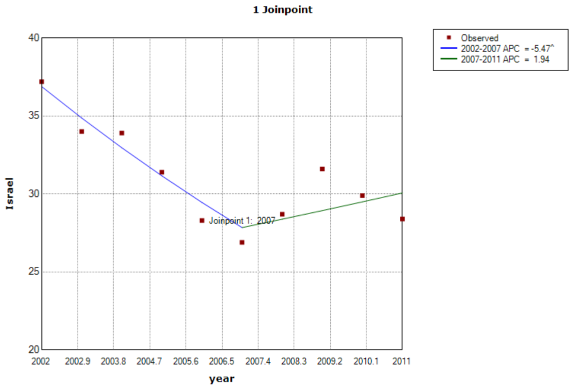

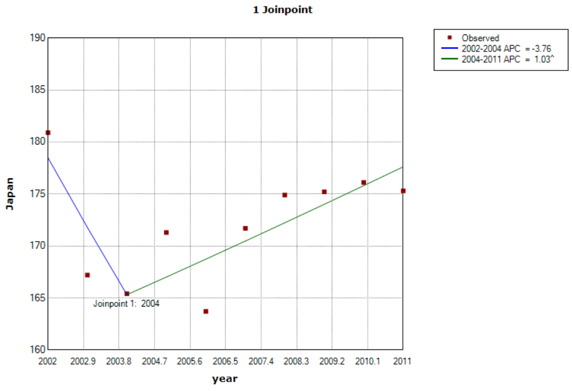

© 2021 Wong MCS et al. *JAMA Network Open.*

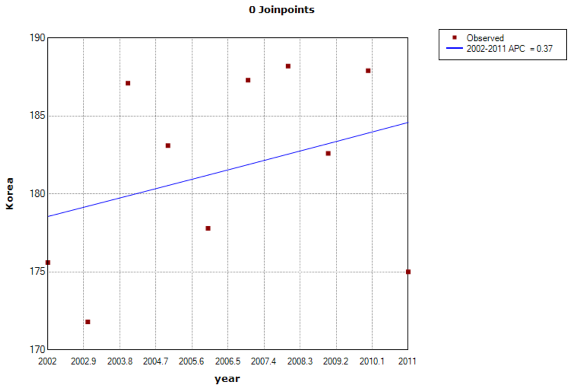

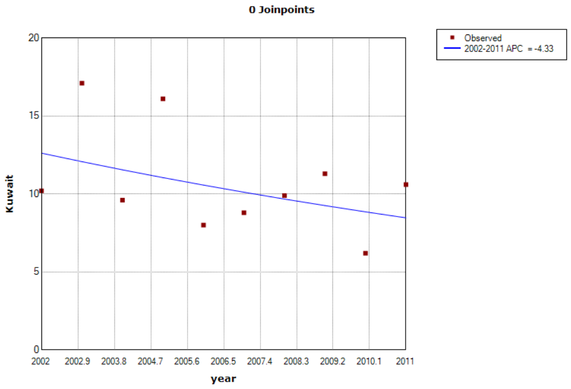

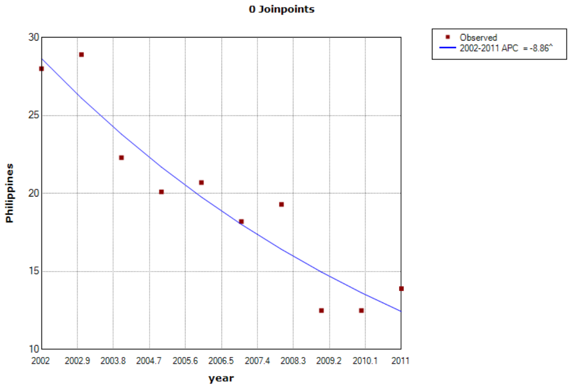

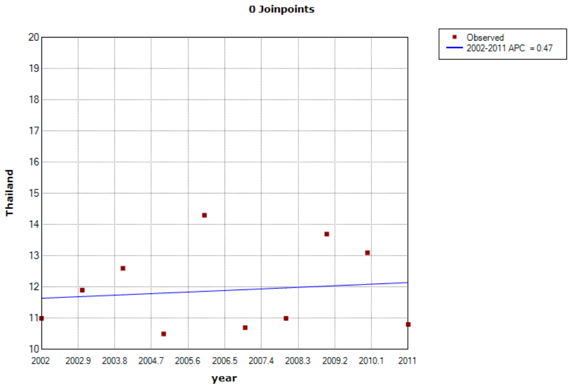

Oceania

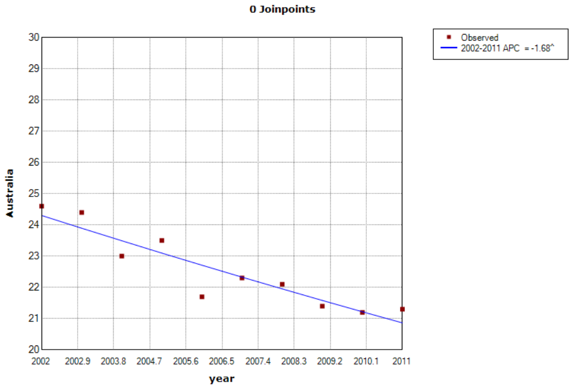

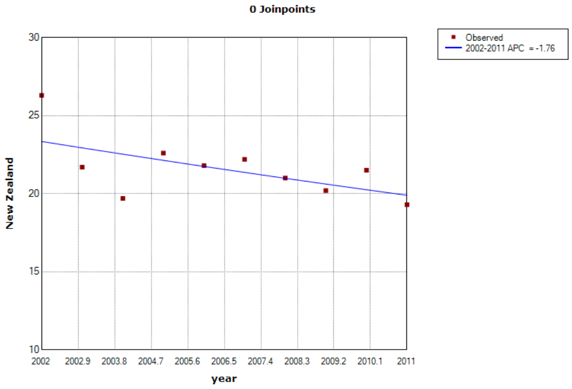

© 2021 Wong MCS et al. *JAMA Network Open.*

Northern America

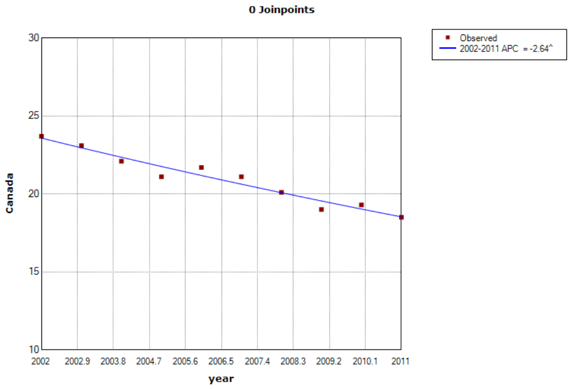

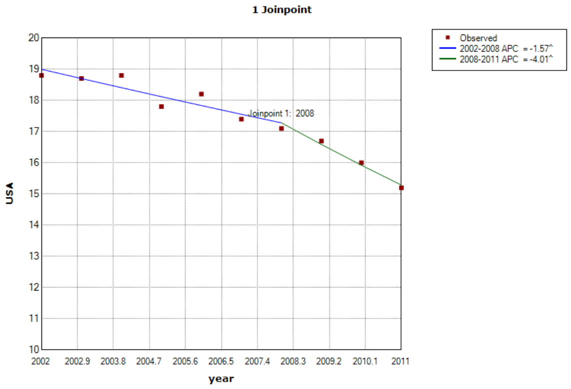

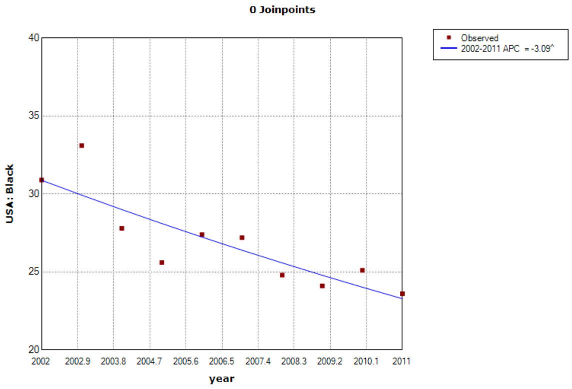

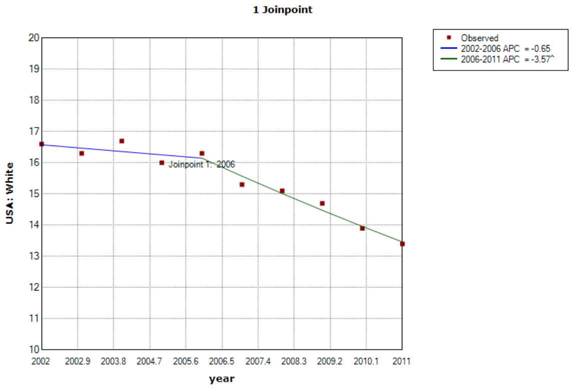

Southern America

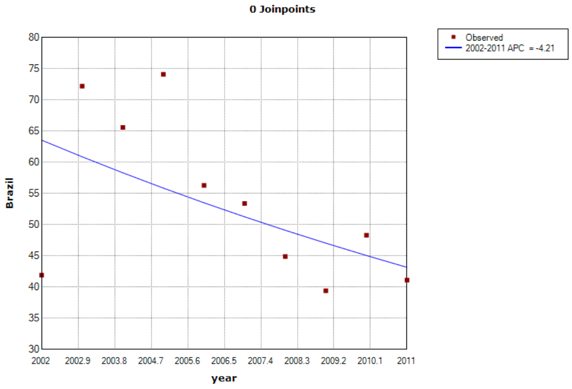

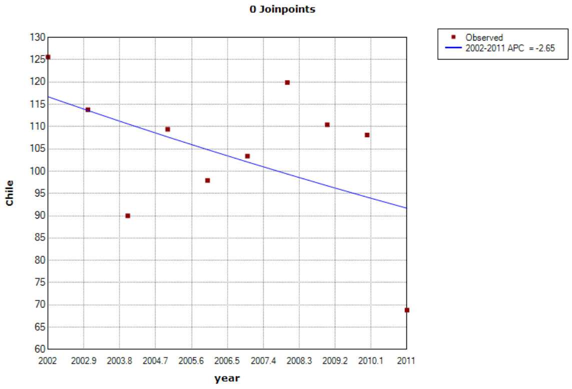

© 2021 Wong MCS et al. *JAMA Network Open.*

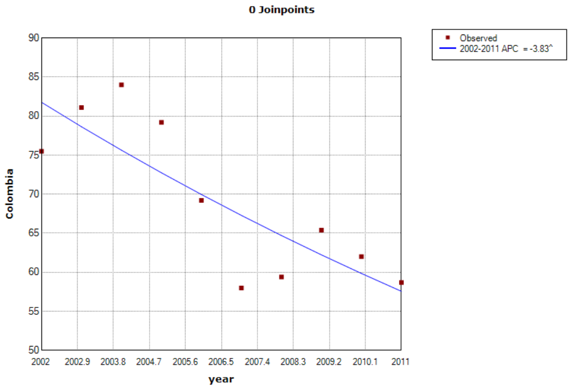

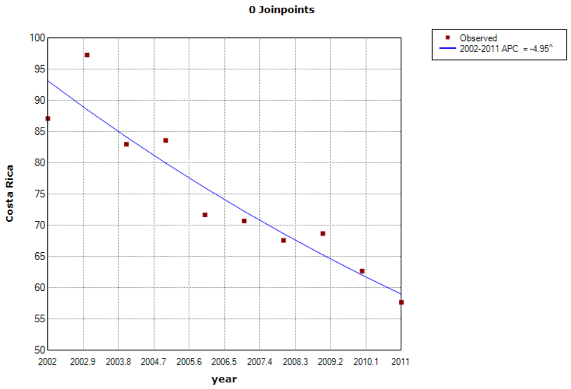

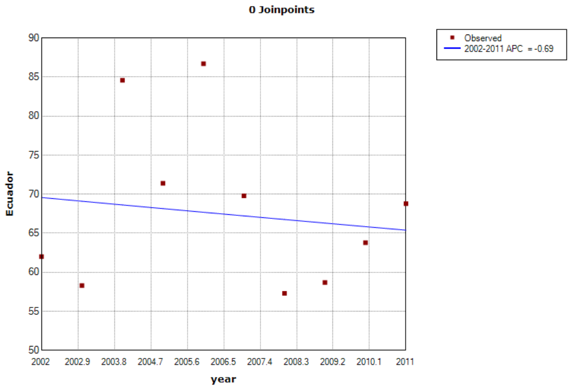

Northern Europe

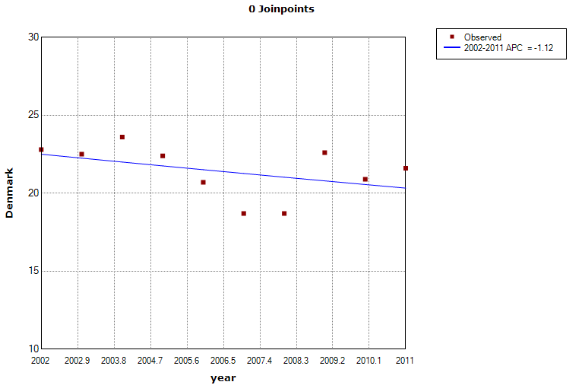

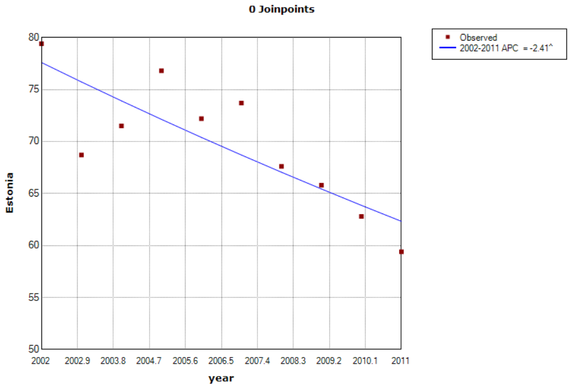

© 2021 Wong MCS et al. *JAMA Network Open.*

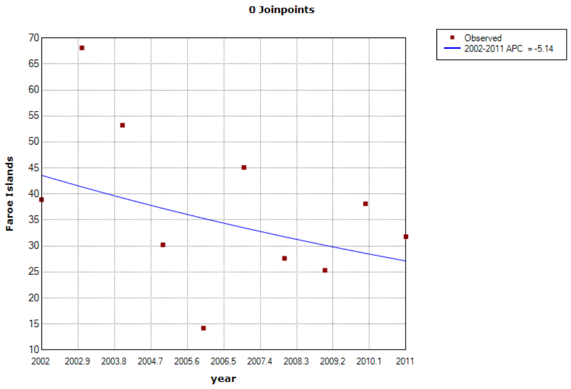

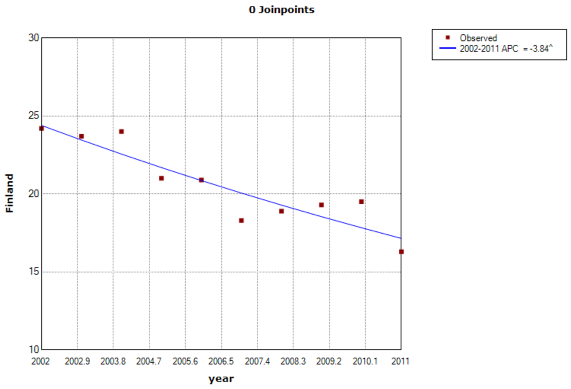

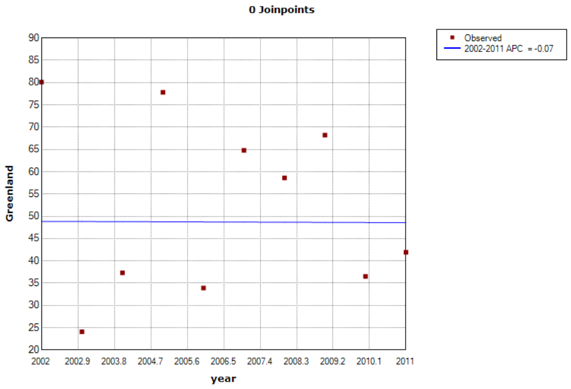

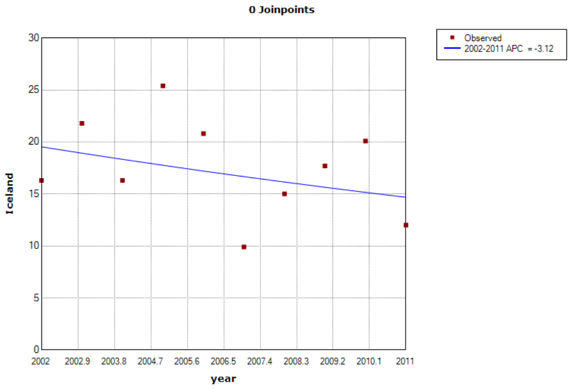

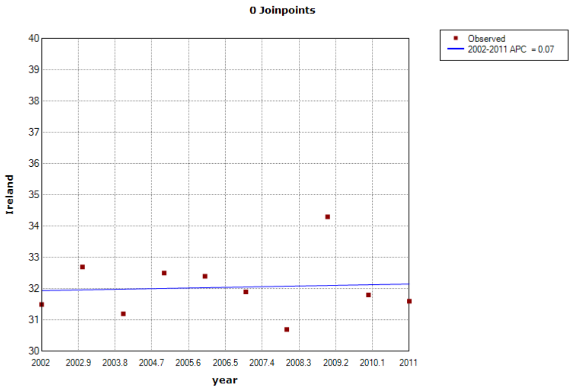

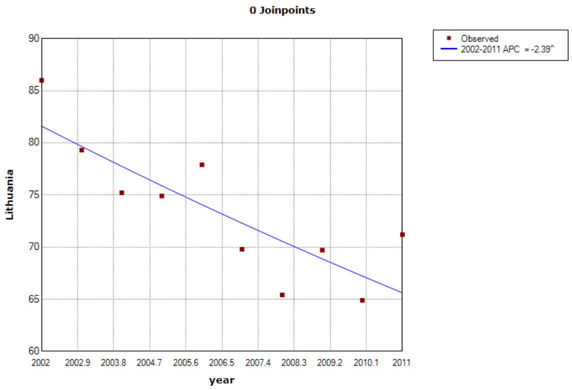

© 2021 Wong MCS et al. *JAMA Network Open.*

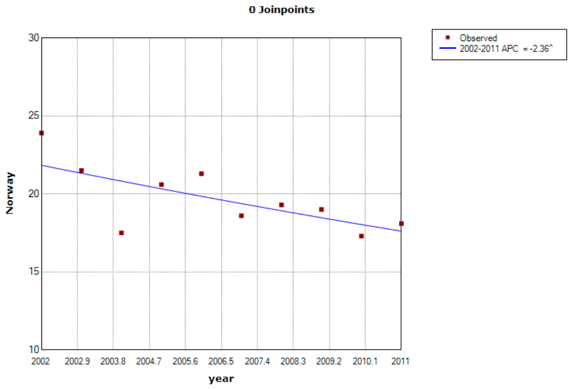

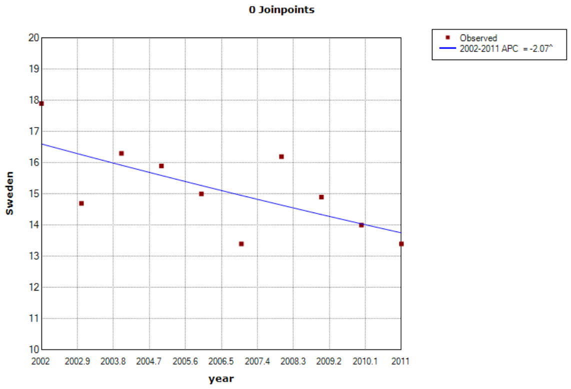

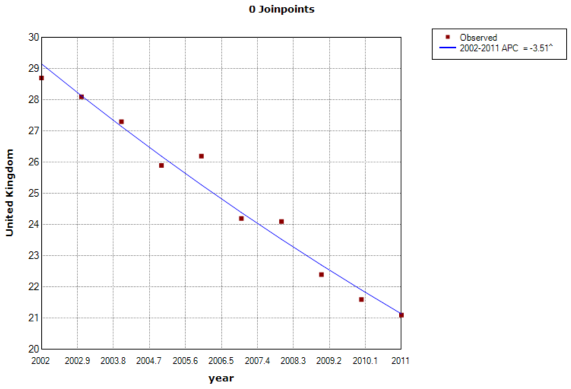

Western Europe

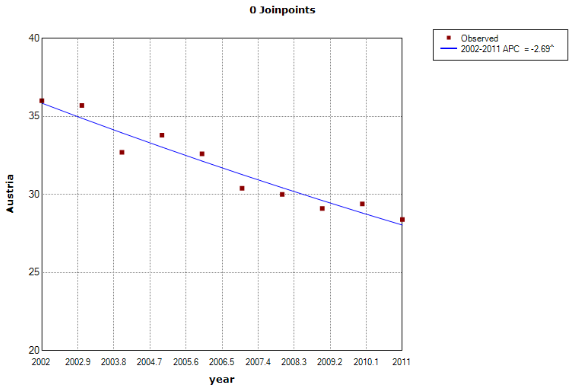

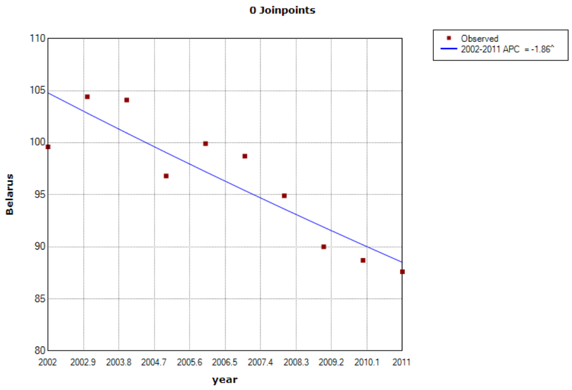

© 2021 Wong MCS et al. *JAMA Network Open.*

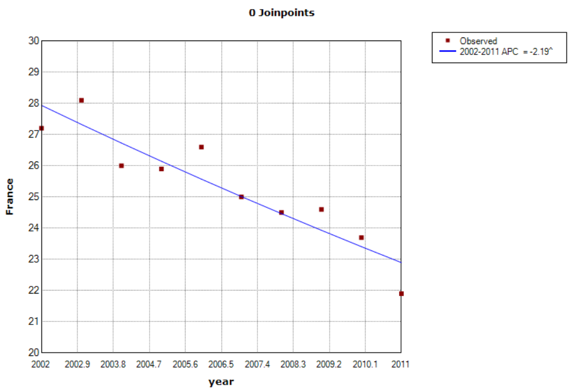

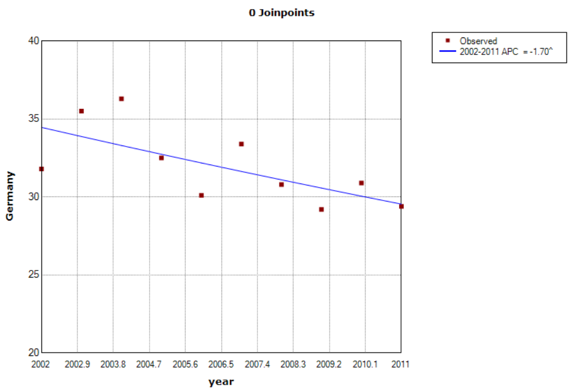

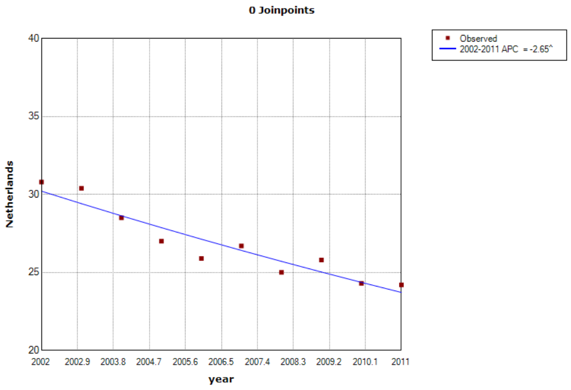

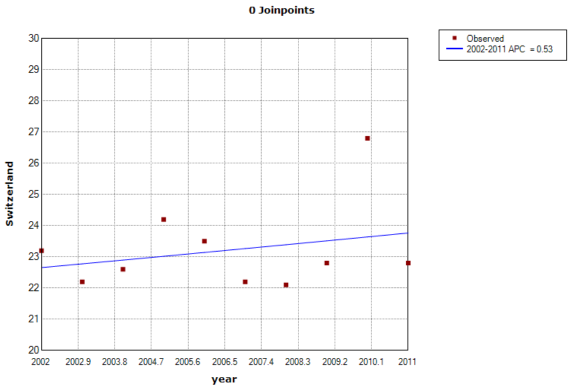

Southern Europe

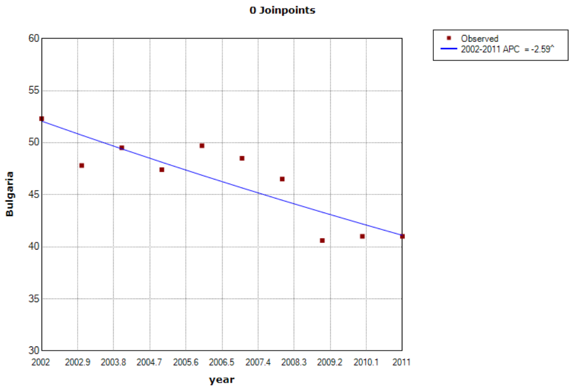

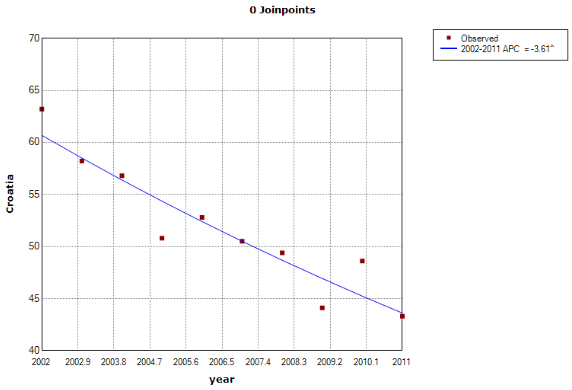

© 2021 Wong MCS et al. *JAMA Network Open.*

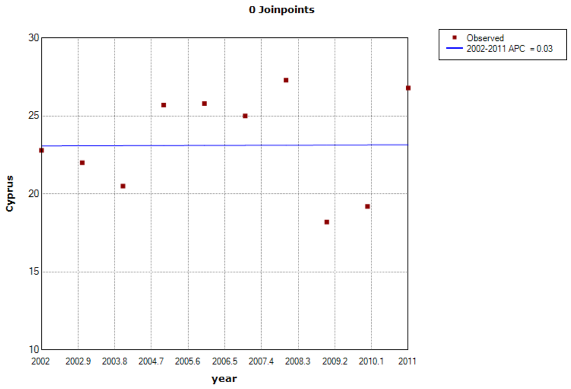

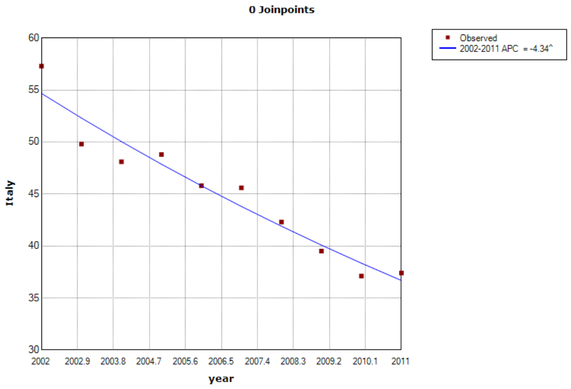

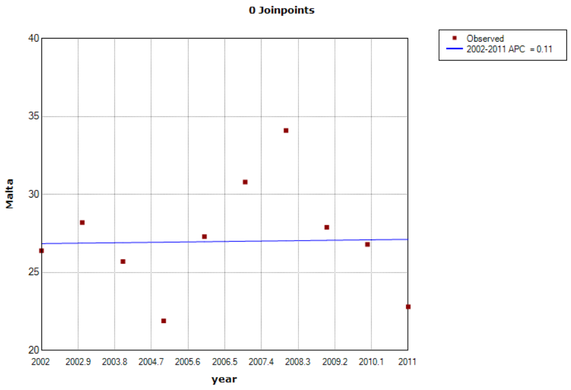

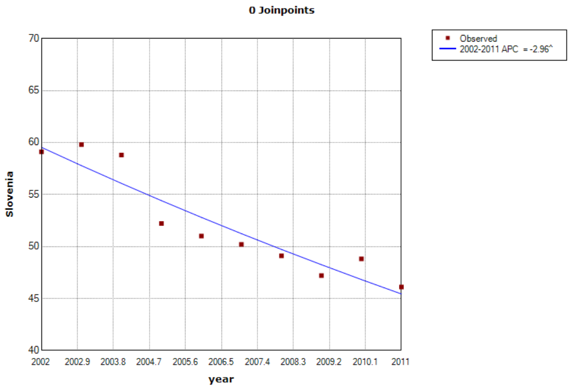

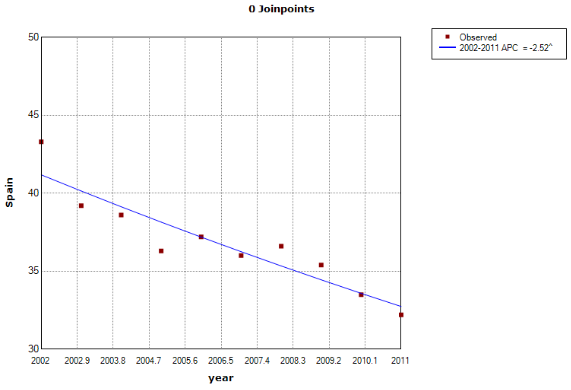

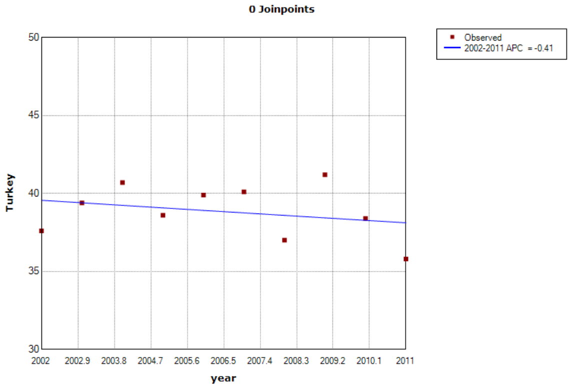

© 2021 Wong MCS et al. *JAMA Network Open.*

Eastern Europe

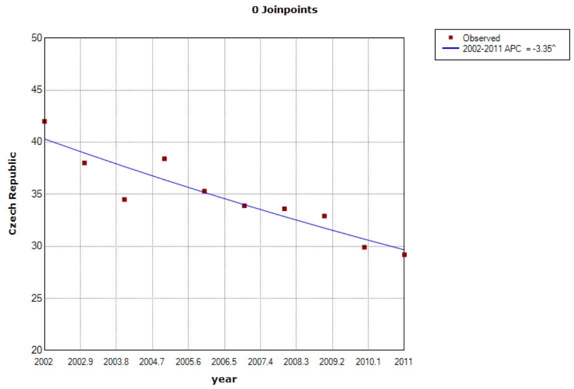

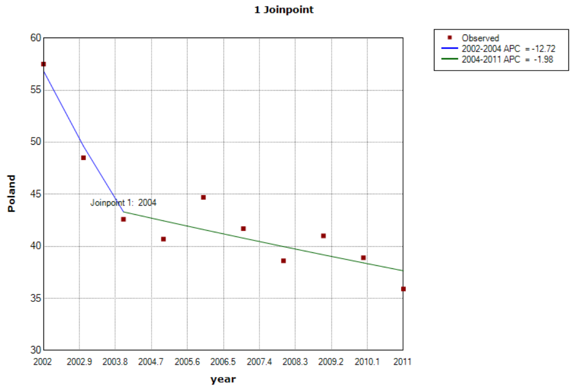

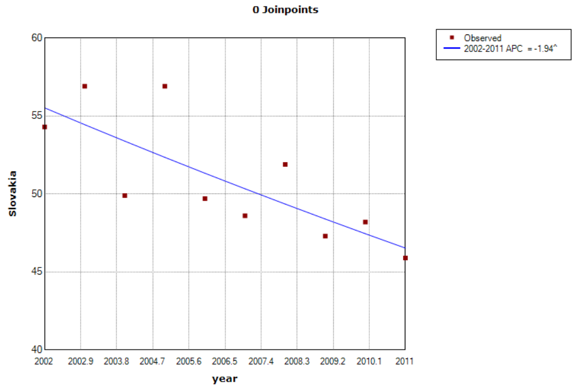

Africa

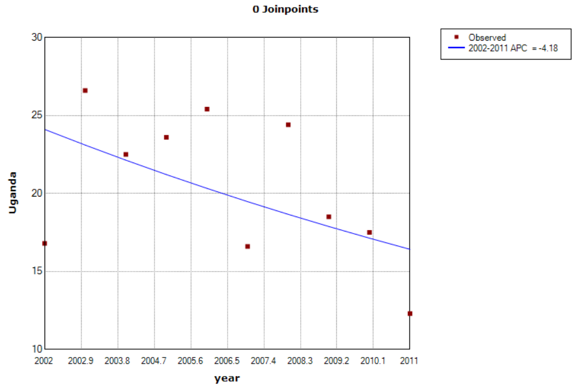

© 2021 Wong MCS et al. *JAMA Network Open.*

f.) Incidence female above 40 years old

Asia

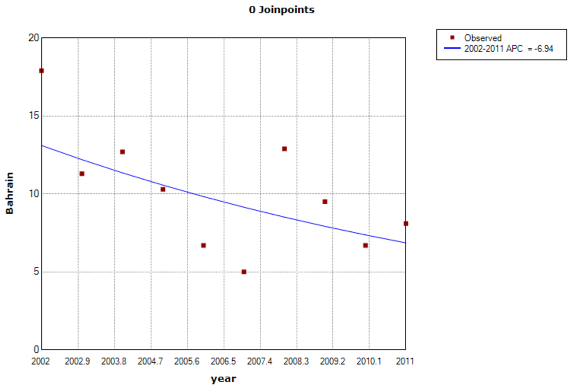

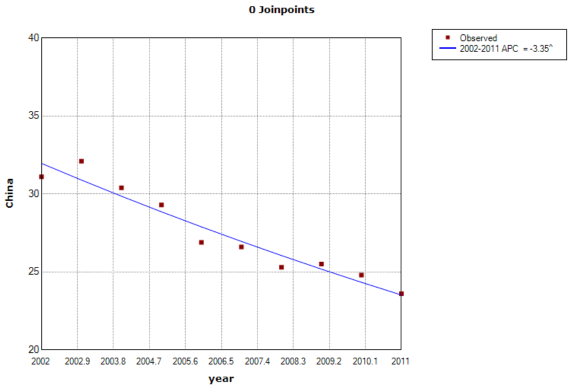

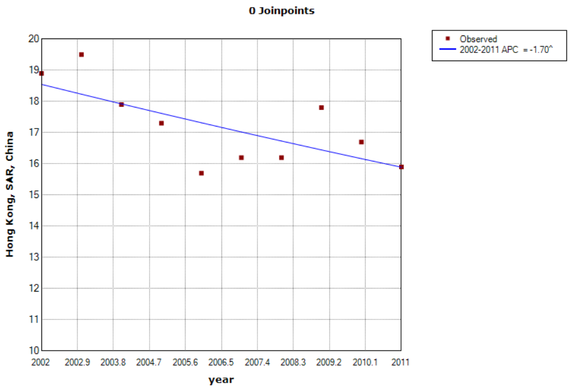

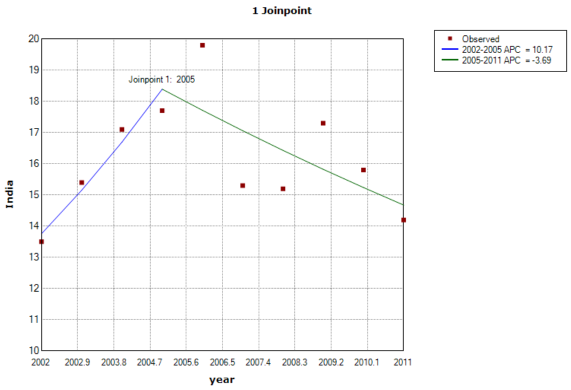

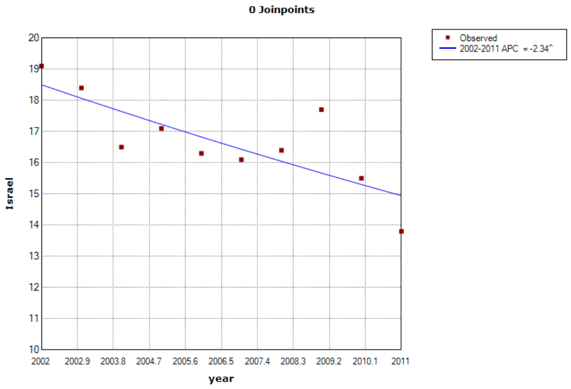

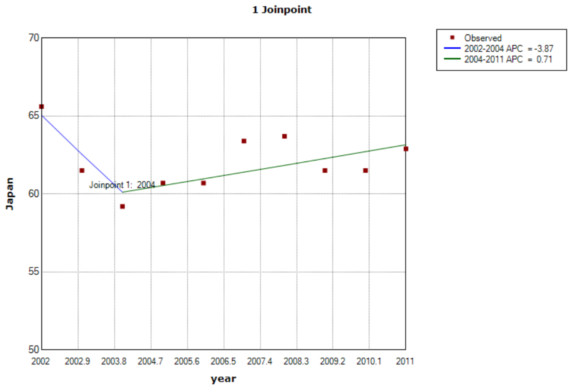

© 2021 Wong MCS et al. *JAMA Network Open.*

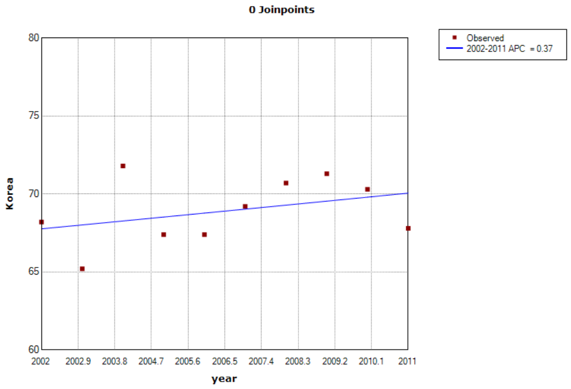

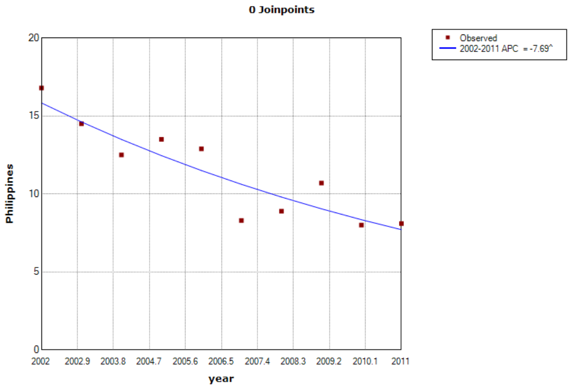

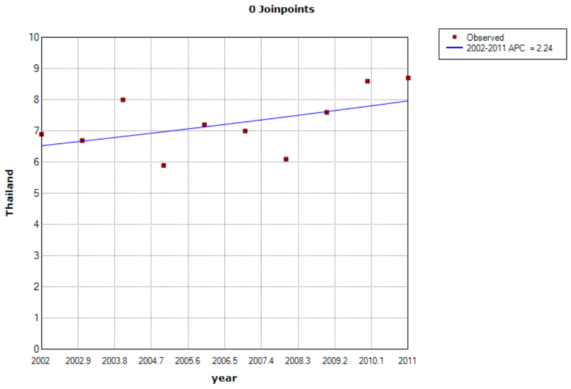

Oceania

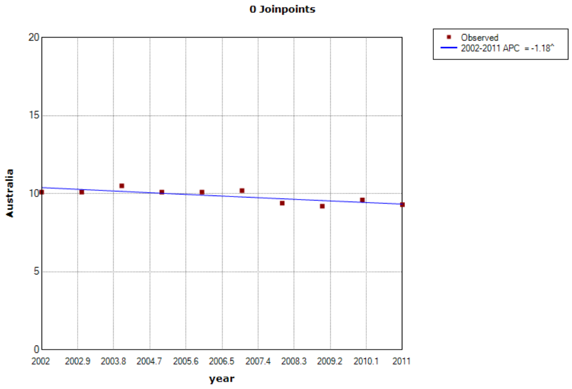

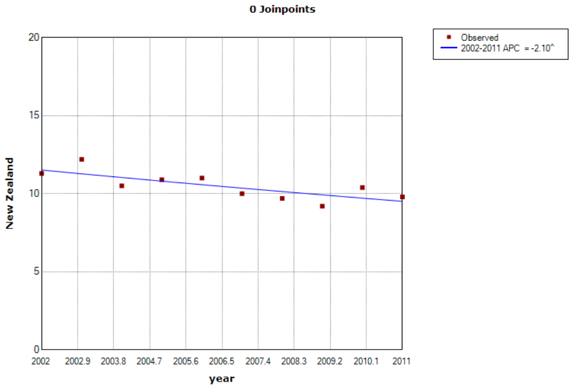

© 2021 Wong MCS et al. *JAMA Network Open.*

Northern America

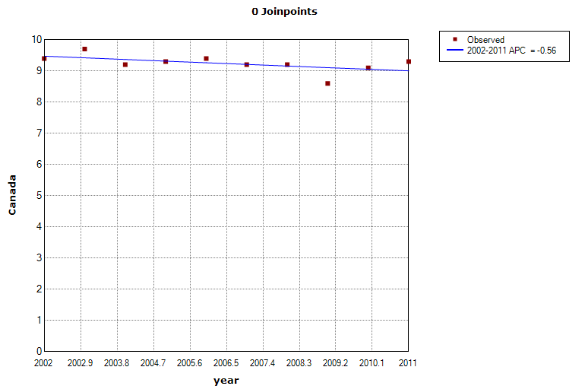

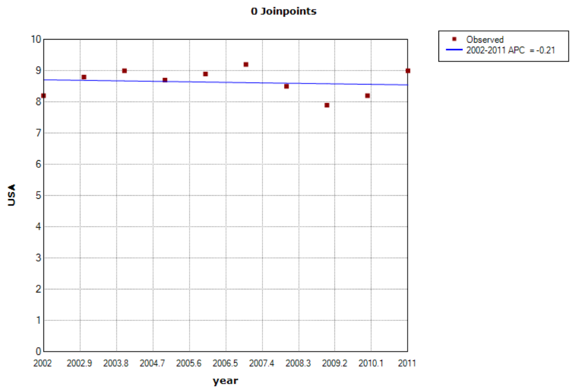

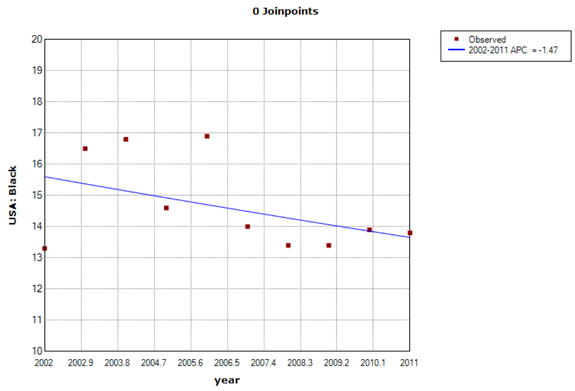

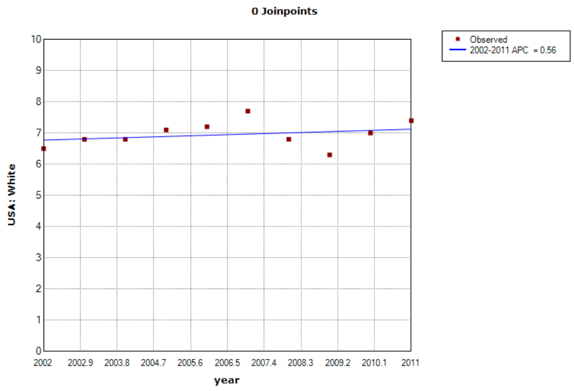

Southern America

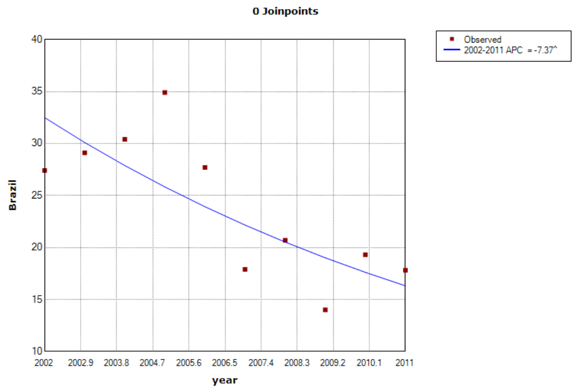

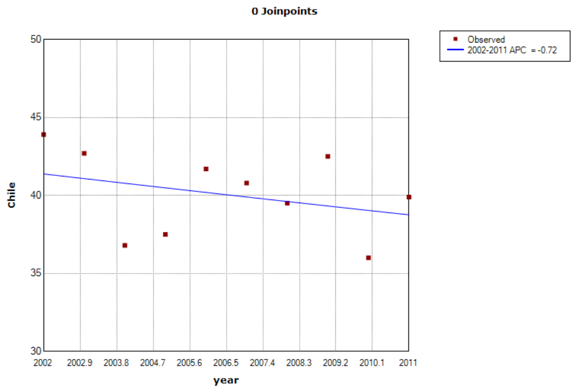

© 2021 Wong MCS et al. *JAMA Network Open.*

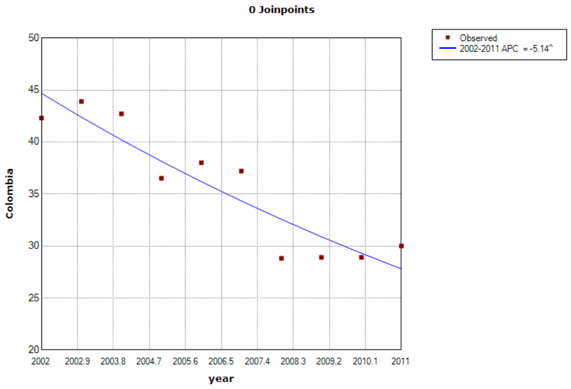

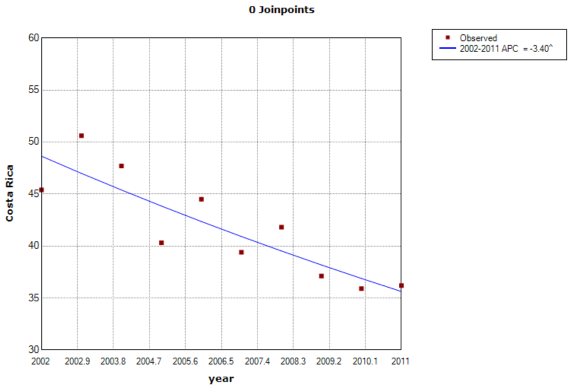

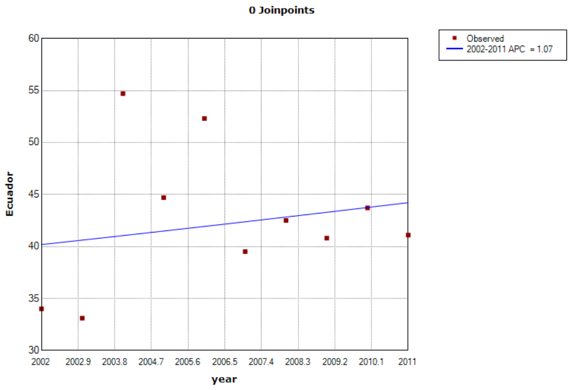

Northern Europe

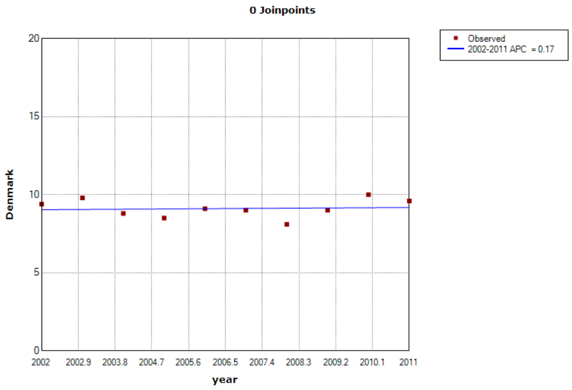

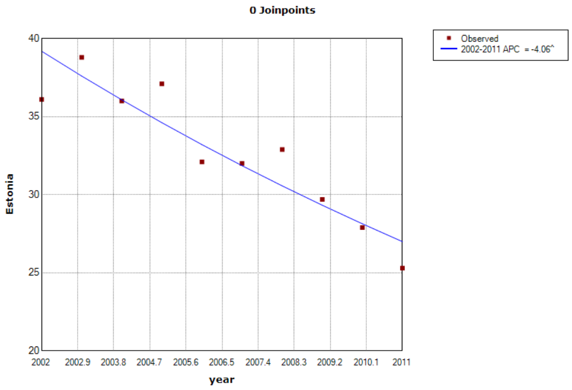

© 2021 Wong MCS et al. *JAMA Network Open.*

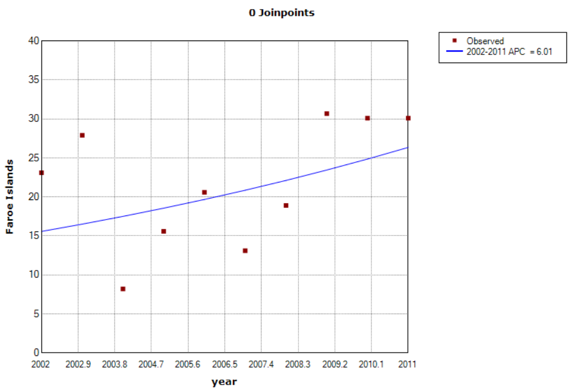

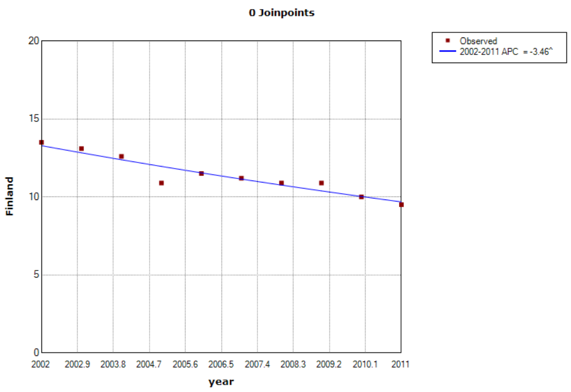

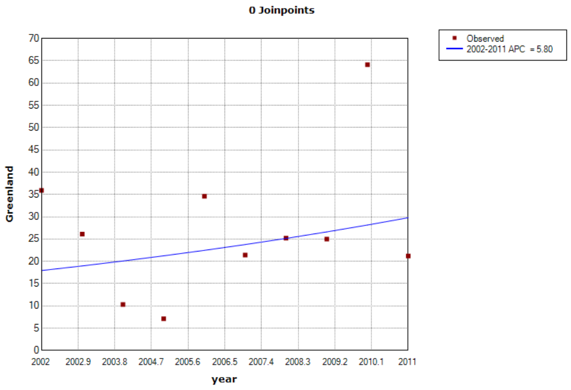

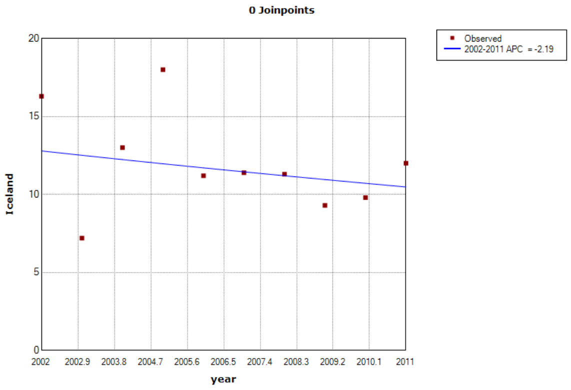

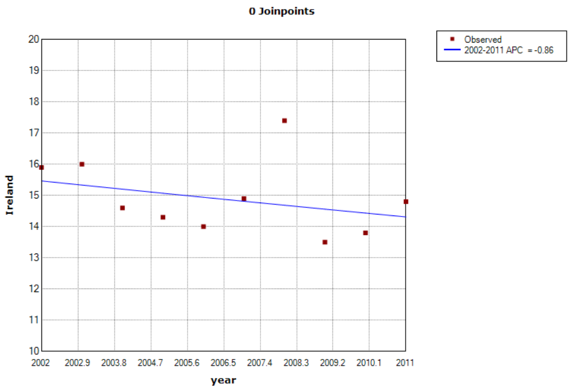

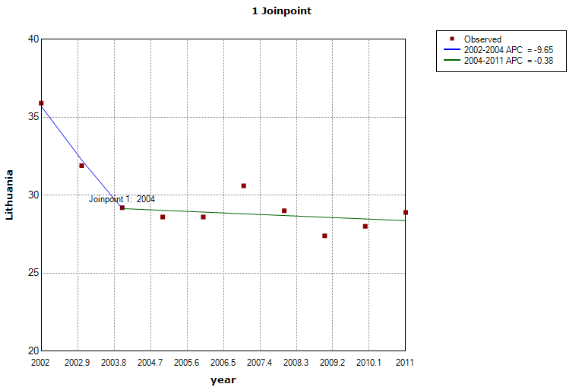

© 2021 Wong MCS et al. *JAMA Network Open.*

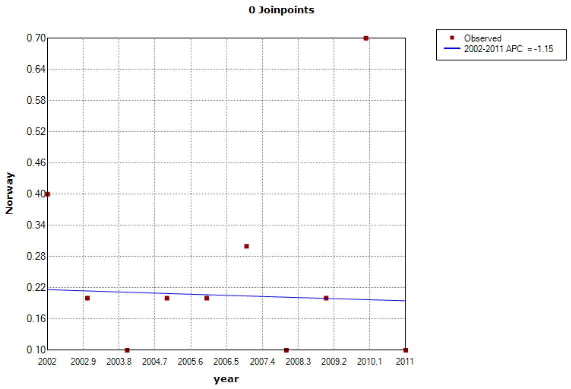

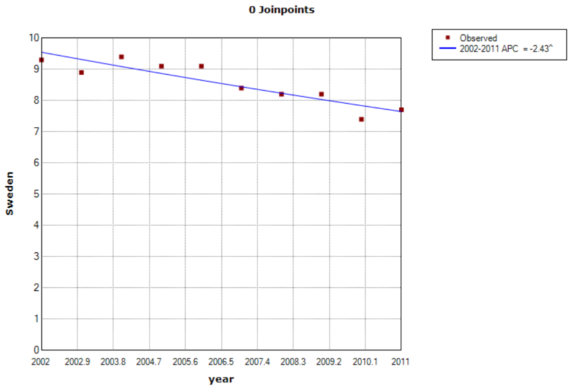

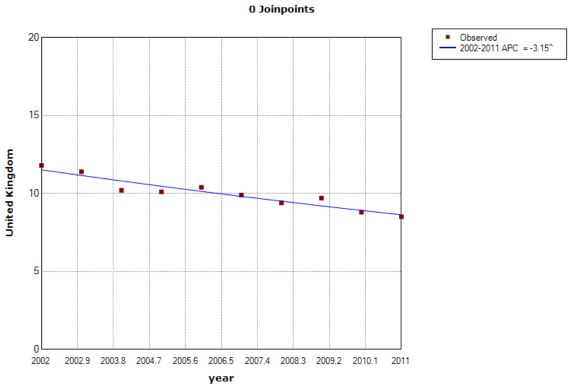

Western Europe

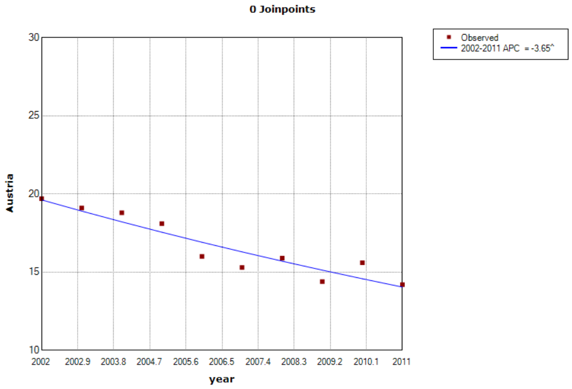

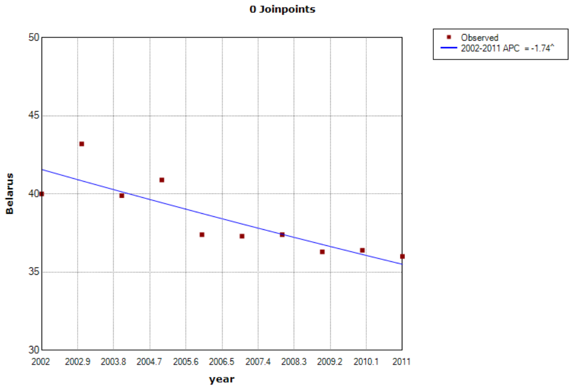

© 2021 Wong MCS et al. *JAMA Network Open.*

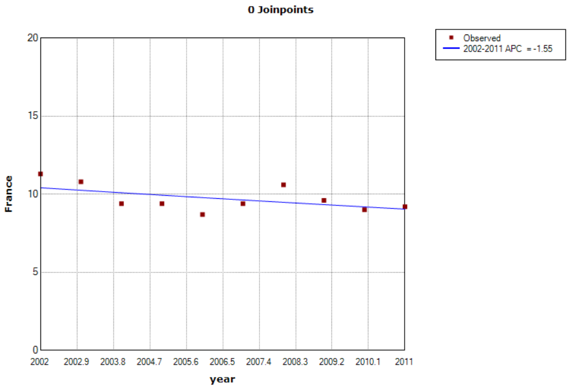

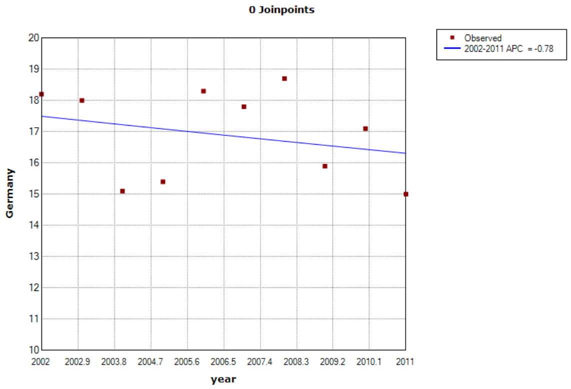

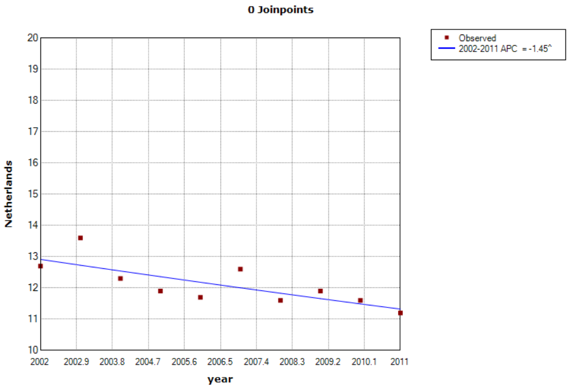

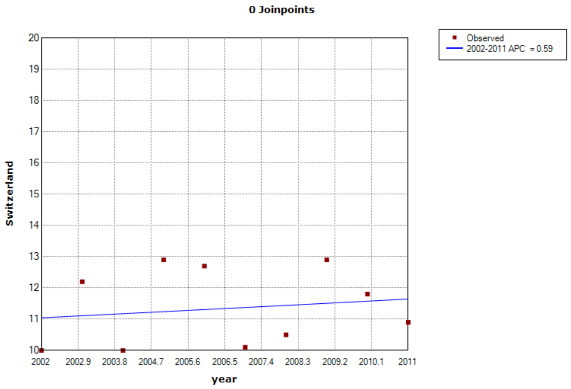

Southern Europe

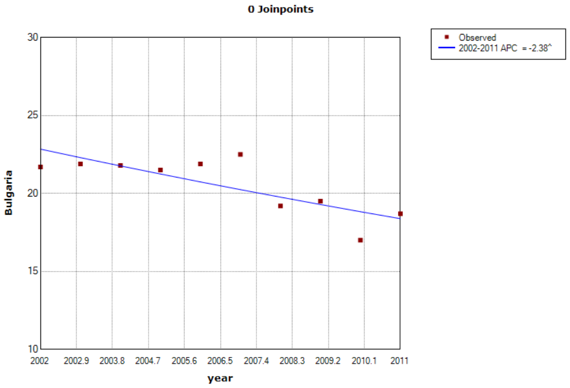

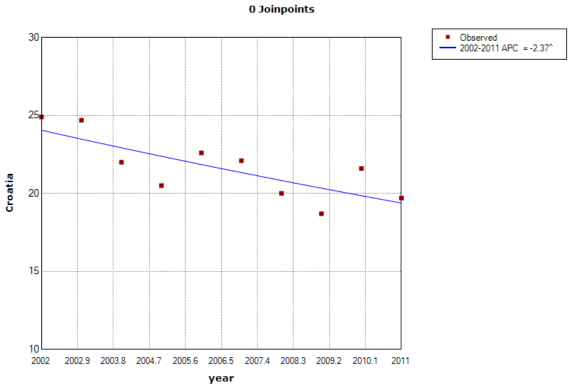

© 2021 Wong MCS et al. *JAMA Network Open.*

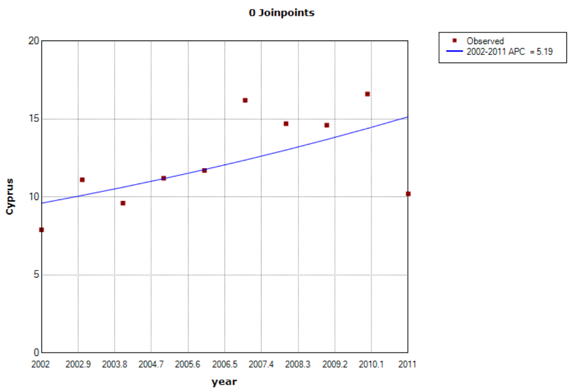

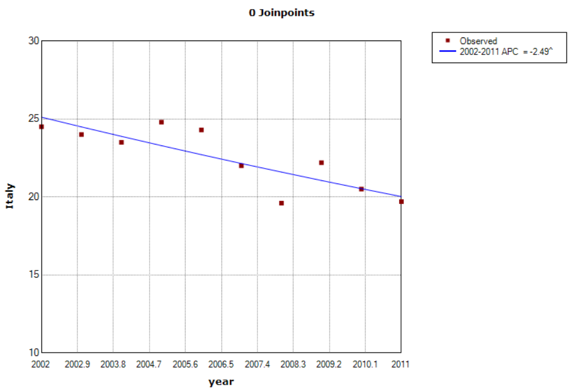

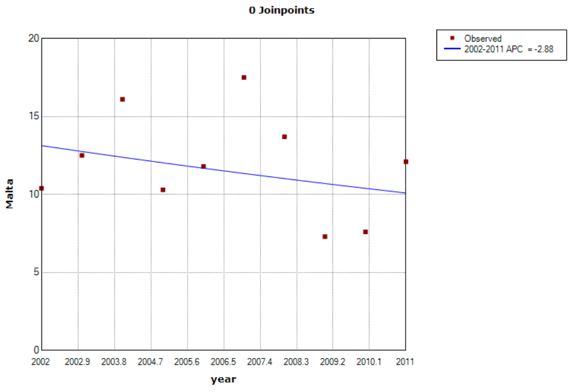

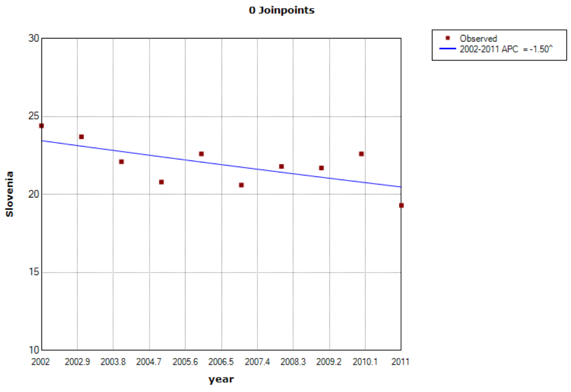

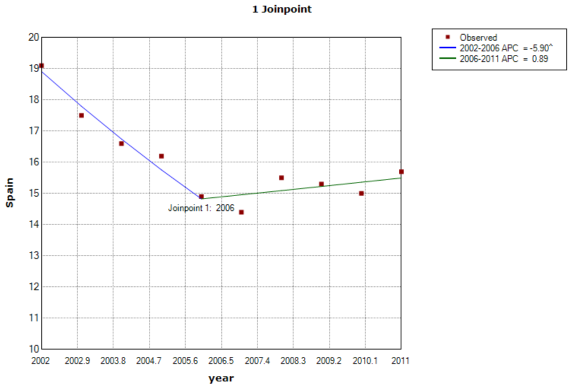

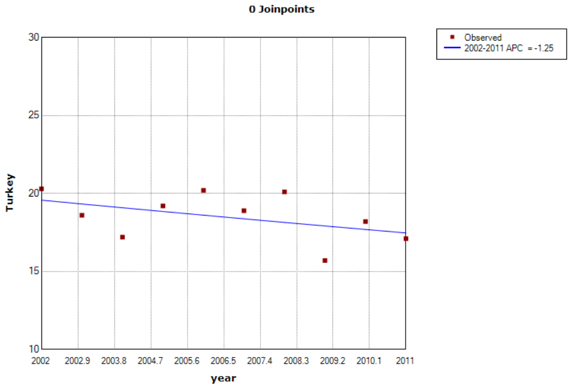

© 2021 Wong MCS et al. *JAMA Network Open.*

Eastern Europe

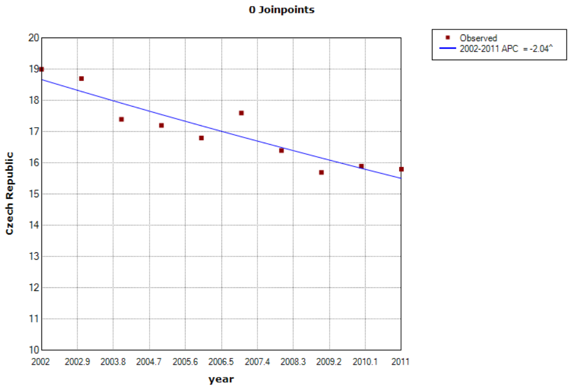

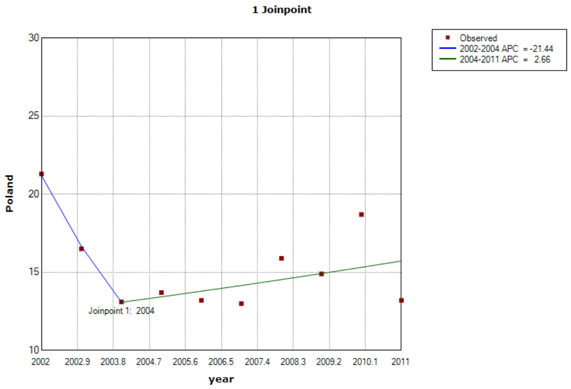

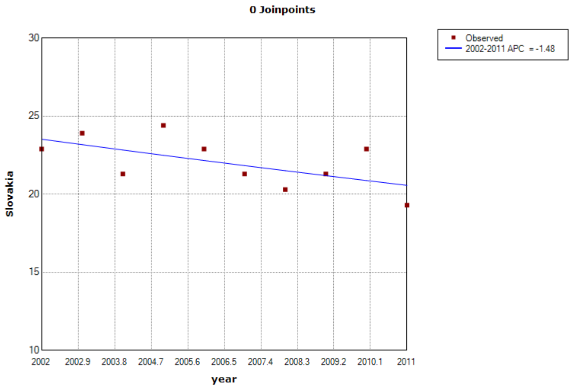

Africa

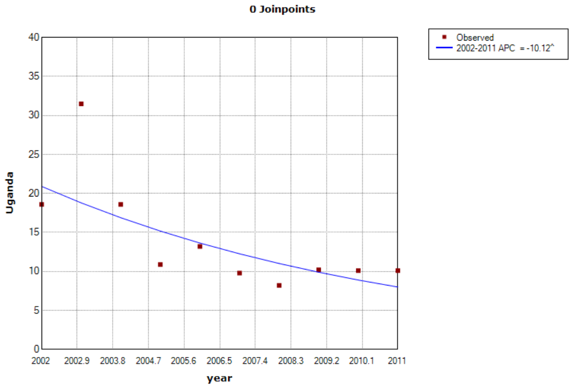

© 2021 Wong MCS et al. *JAMA Network Open.*

g.) Mortality male all ages

Asia

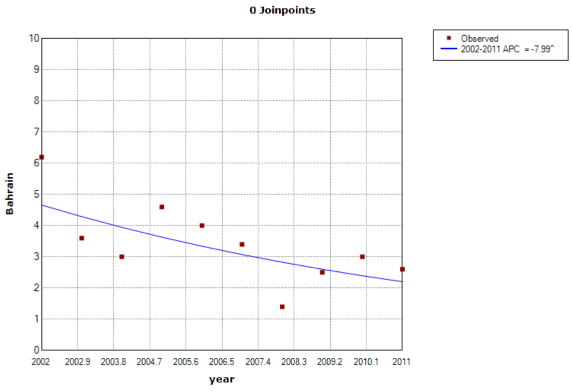

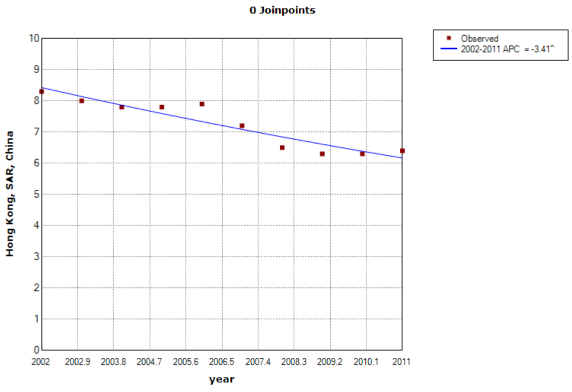

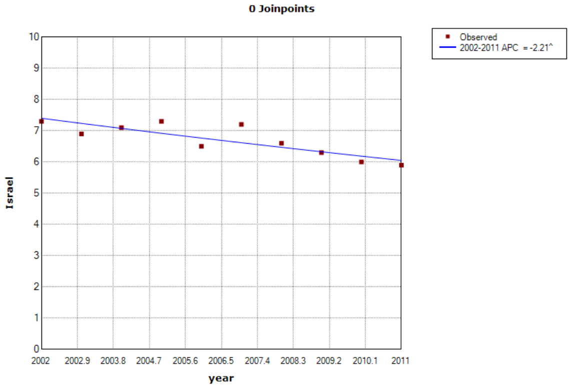

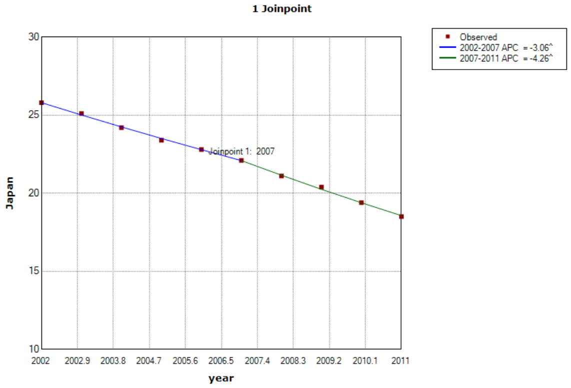

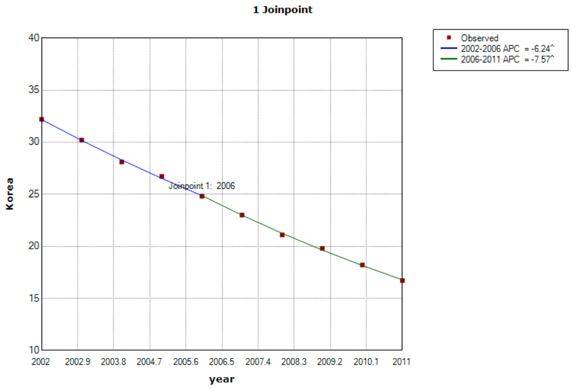

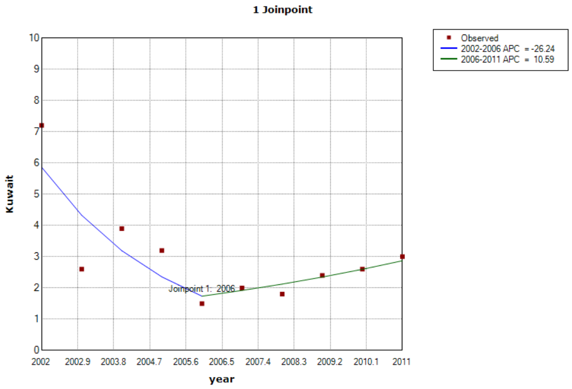

© 2021 Wong MCS et al. *JAMA Network Open.*

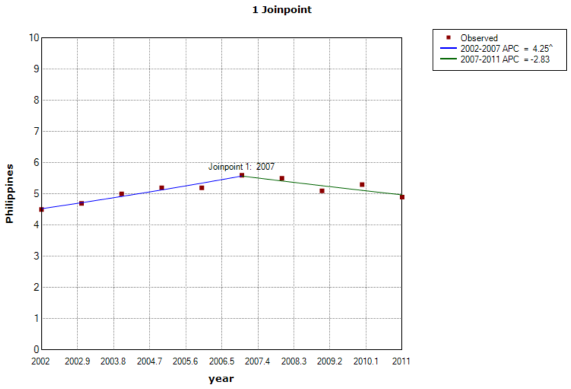

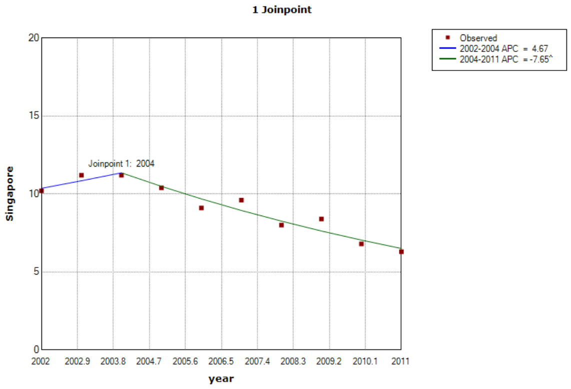

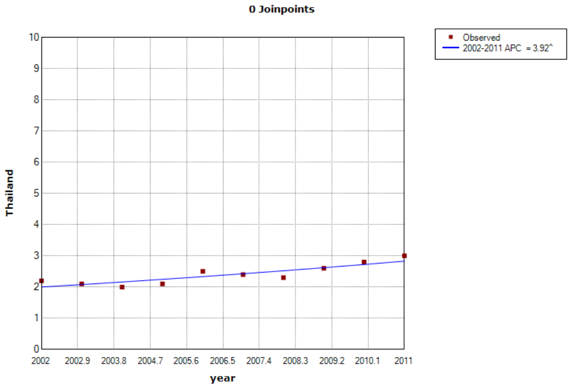

Oceania

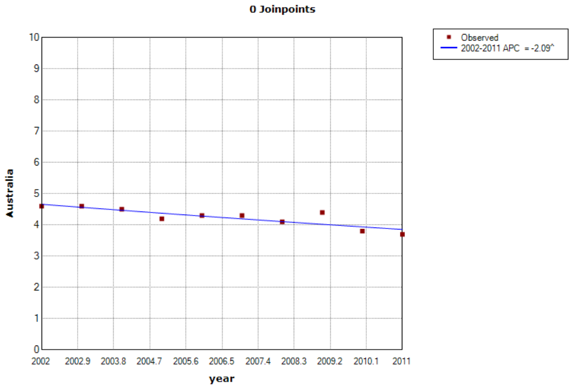

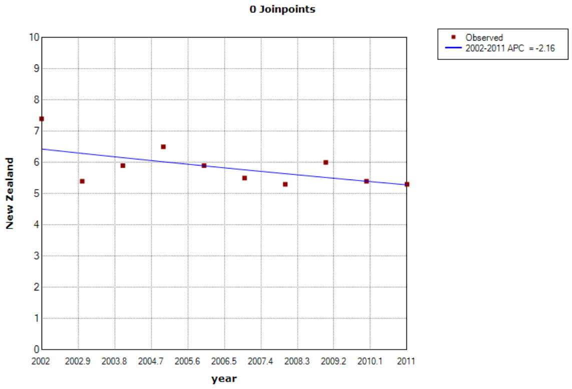

© 2021 Wong MCS et al. *JAMA Network Open.*

Northern America

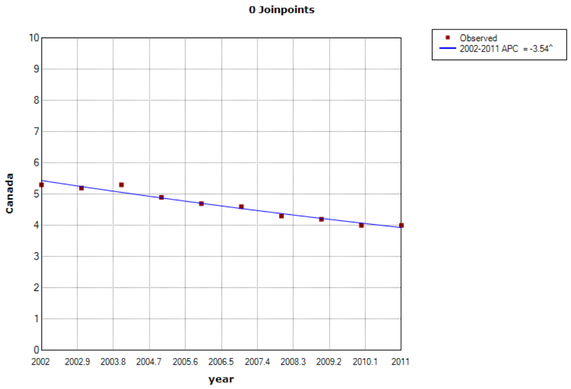

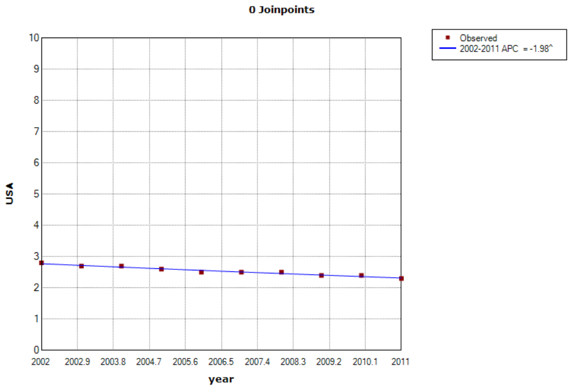

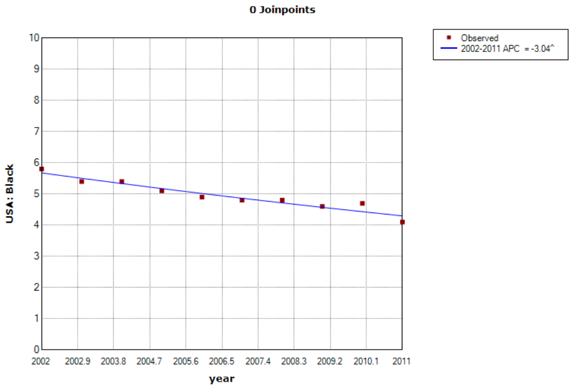

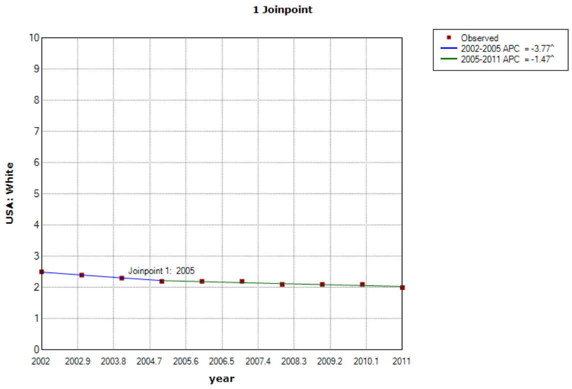

Southern America

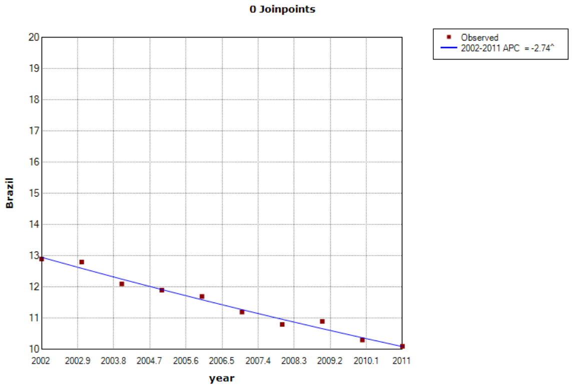

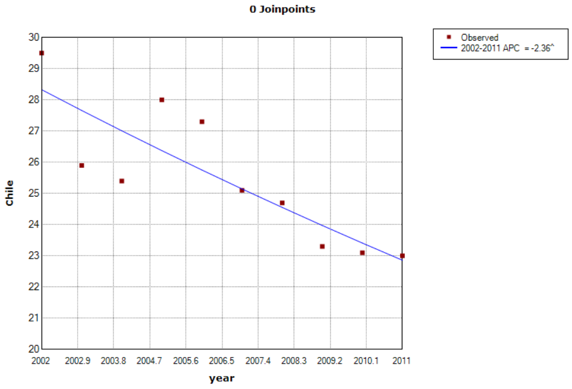

© 2021 Wong MCS et al. *JAMA Network Open.*

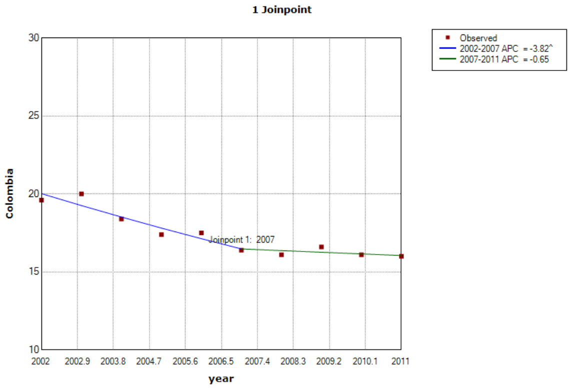

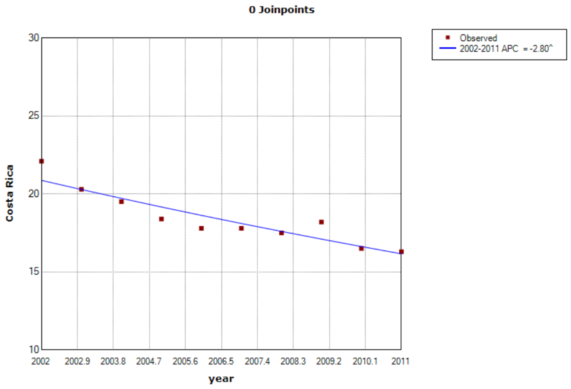

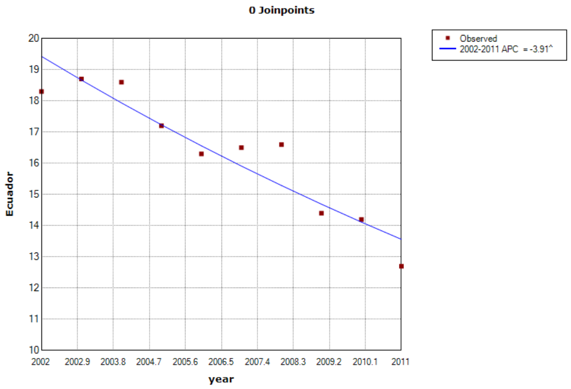

Northern Europe

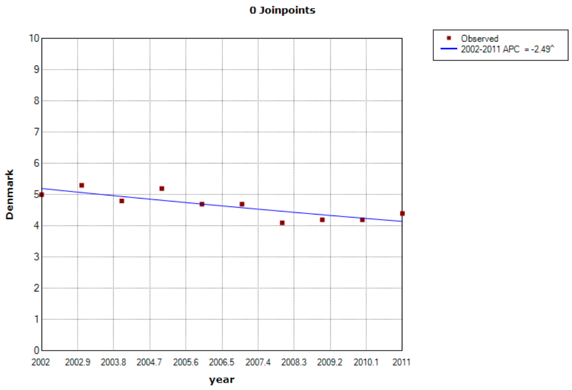

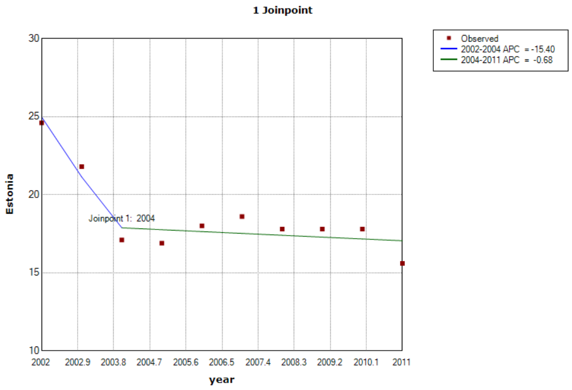

© 2021 Wong MCS et al. *JAMA Network Open.*

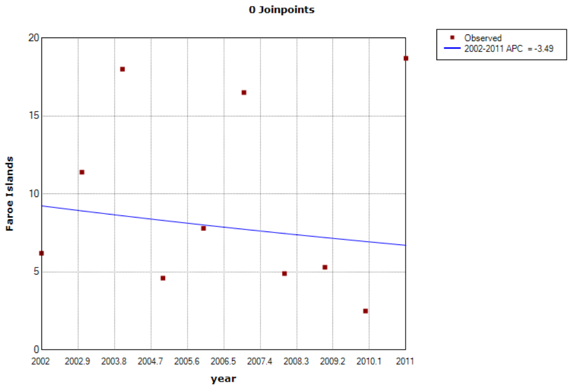
0 Joinpoints
Observed
2002-2011 APC = -3.49
Faroe Islands
year

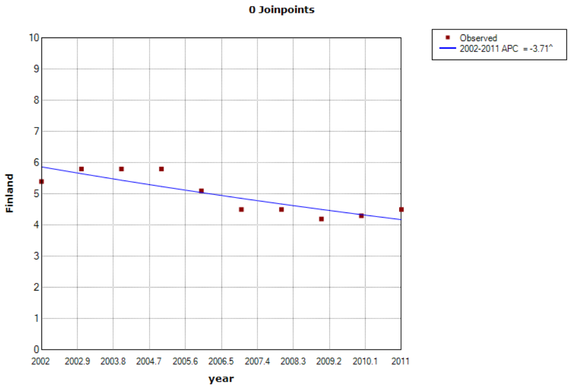
0 Joinpoints
Observed
2002-2011 APC = -3.71^
Finland
year

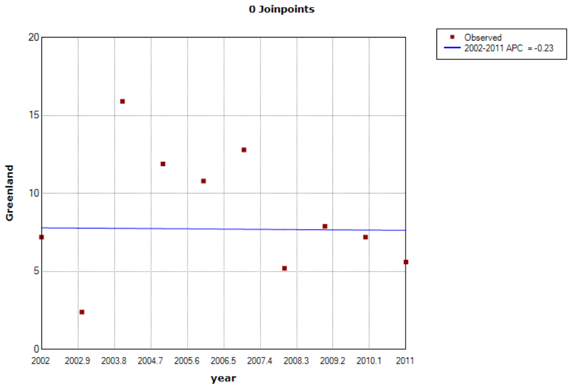
0 Joinpoints
Observed
2002-2011 APC = -0.23
Greenland
year

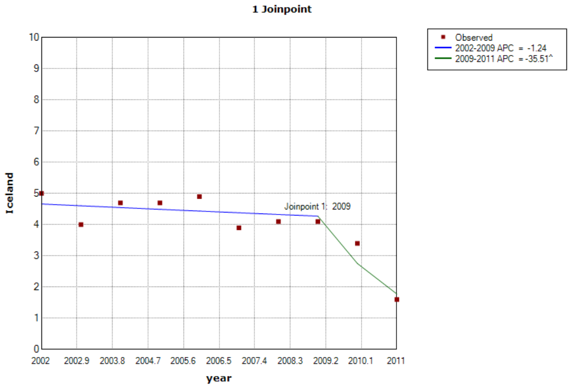
1 Joinpoint
Observed
2002-2009 APC = -1.24
2009-2011 APC = -35.51^
Joinpoint 1: 2009
Iceland
year

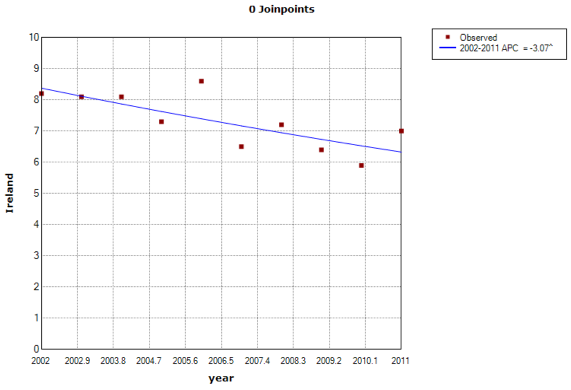
0 Joinpoints
Observed
2002-2011 APC = -3.07^
Ireland
year

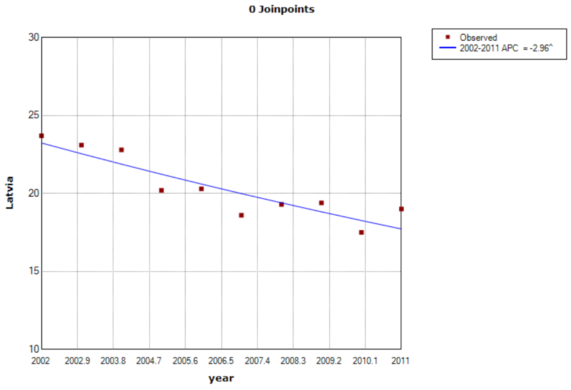
0 Joinpoints
Observed
2002-2011 APC = -2.96^
Latvia
year

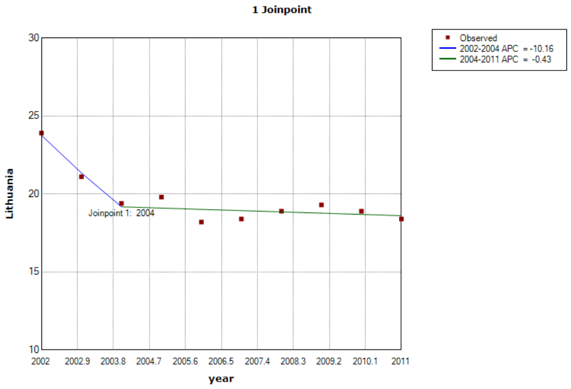

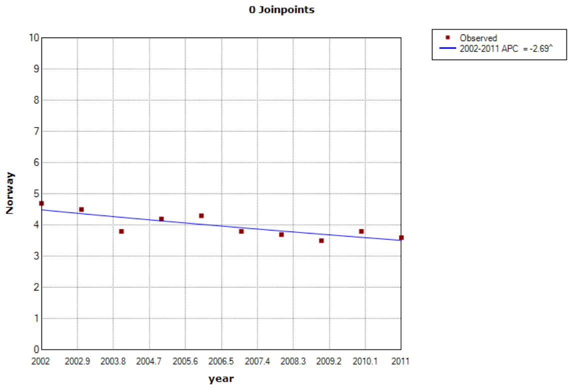

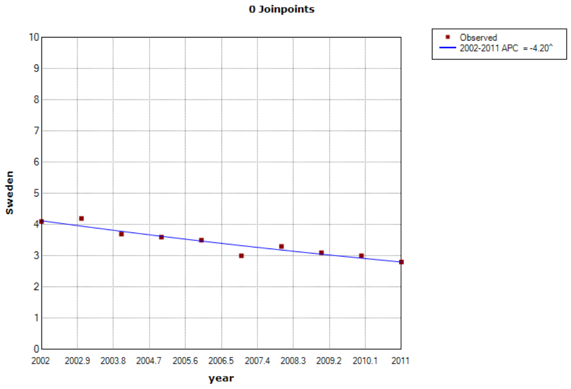

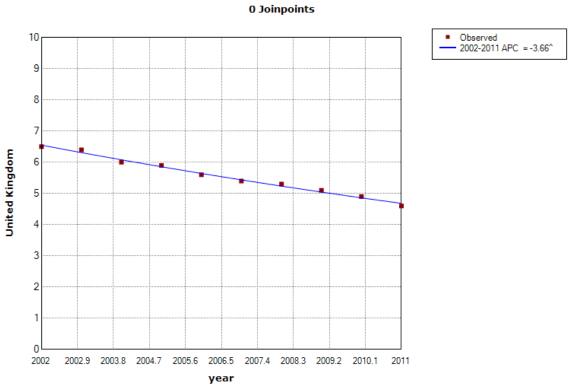

Western Europe

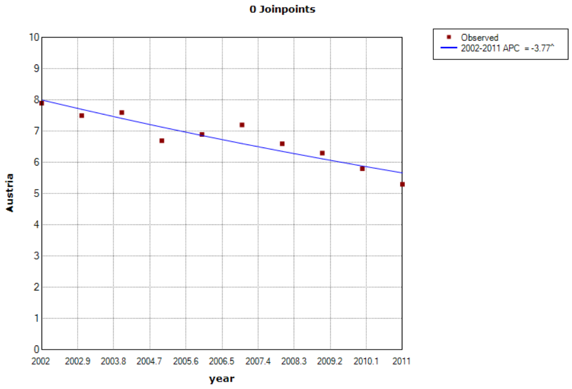

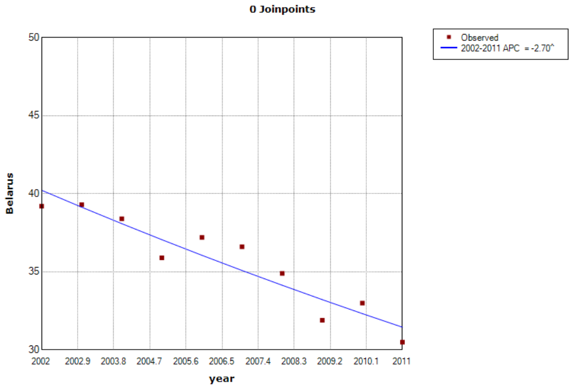

© 2021 Wong MCS et al. *JAMA Network Open.*

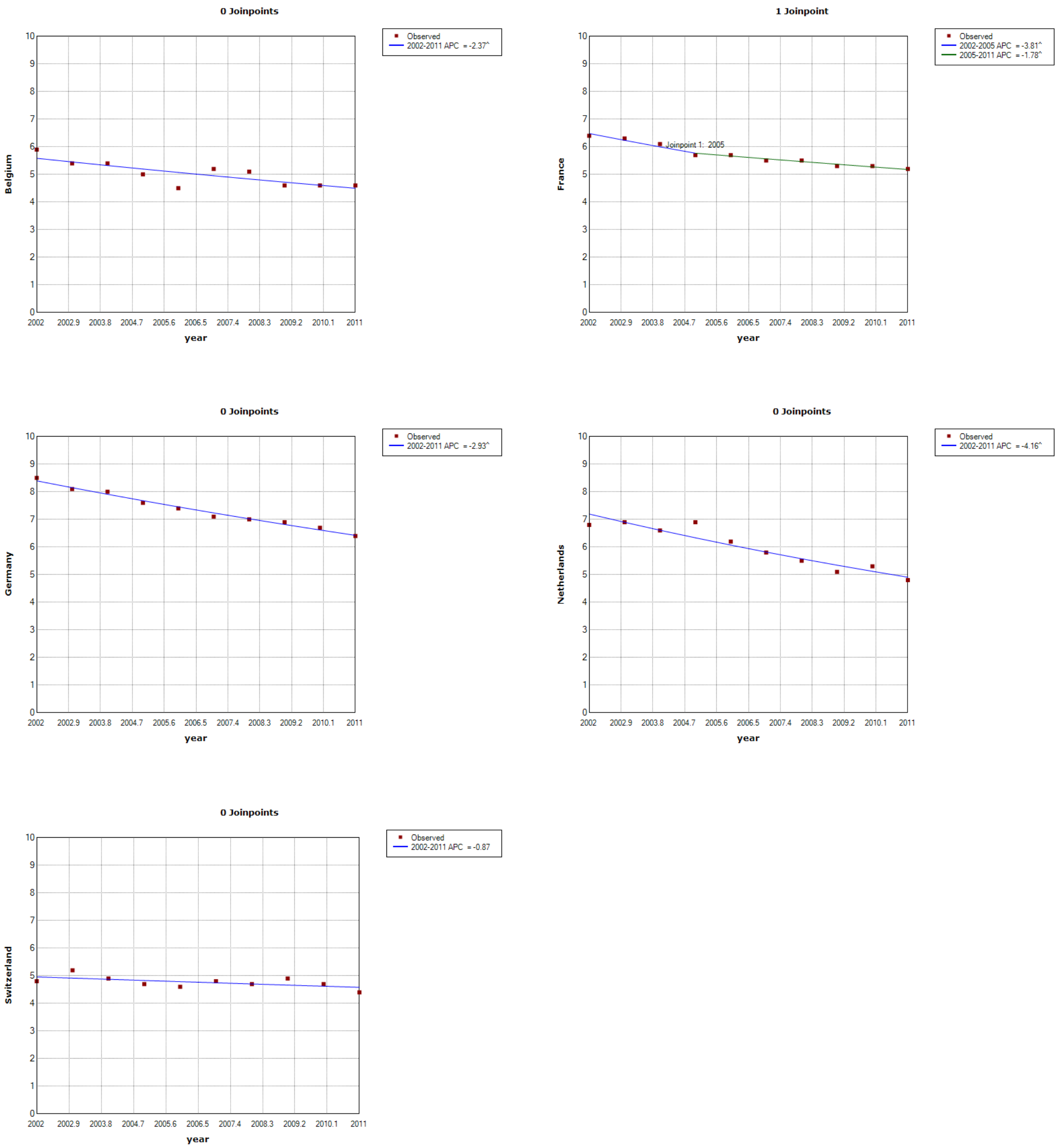

© 2021 Wong MCS et al. *JAMA Network Open.*

Southern Europe

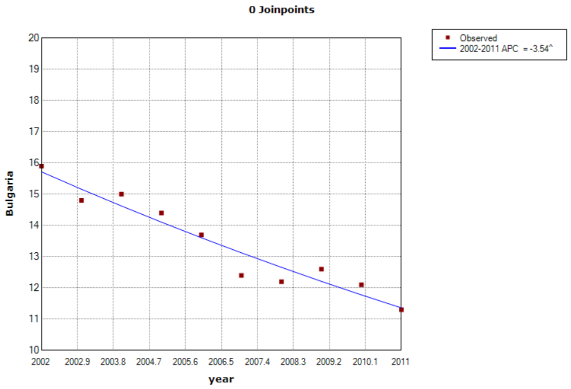

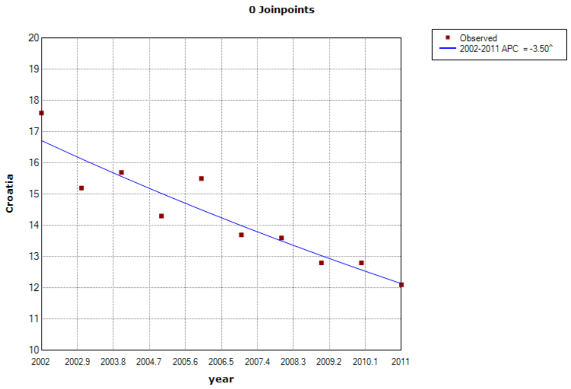

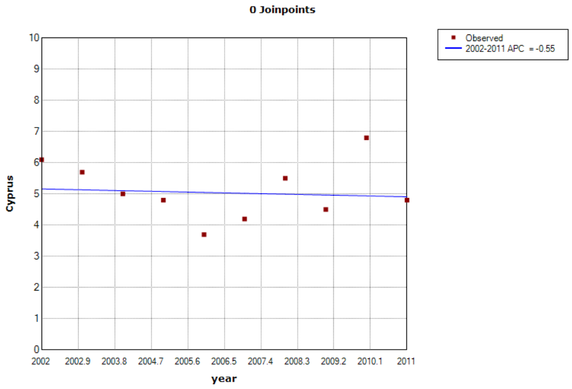

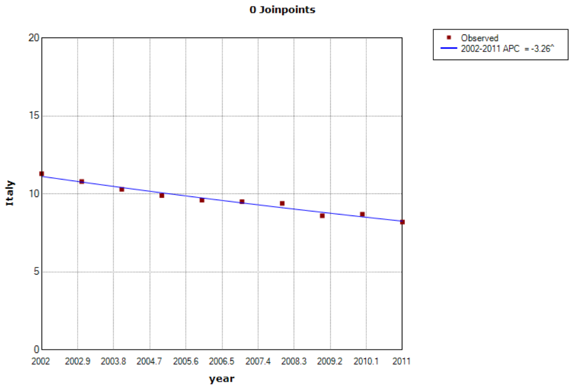

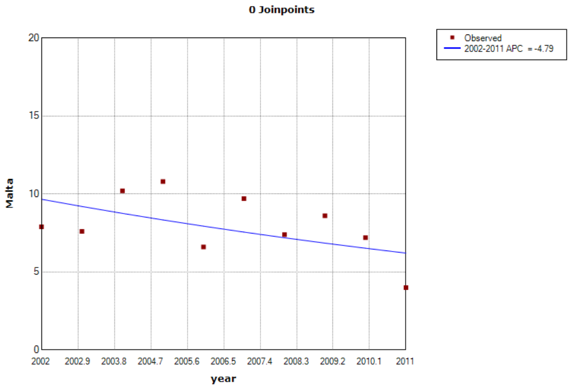

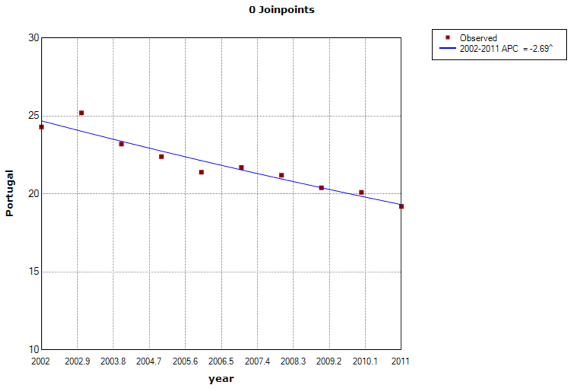

© 2021 Wong MCS et al. *JAMA Network Open.*

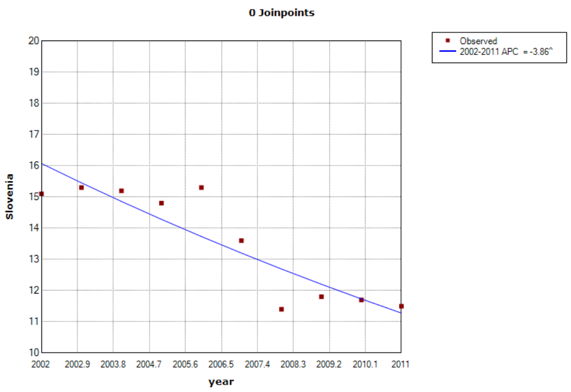

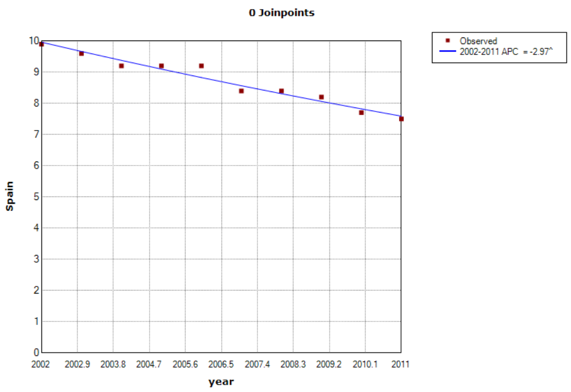

Eastern Europe

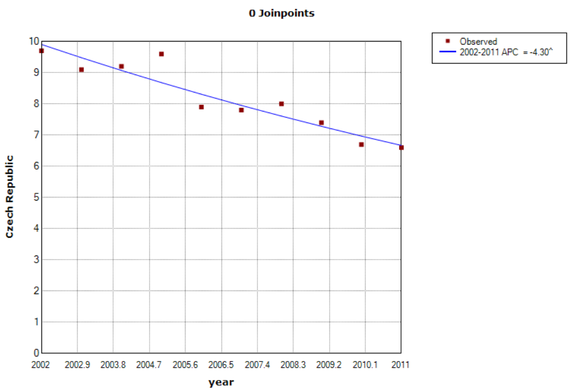

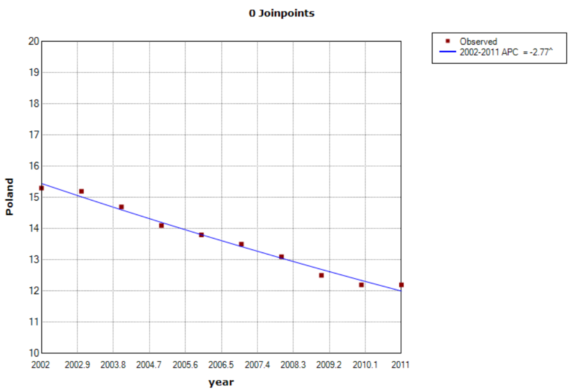

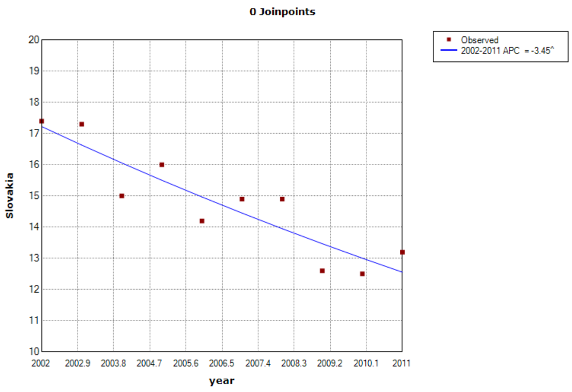

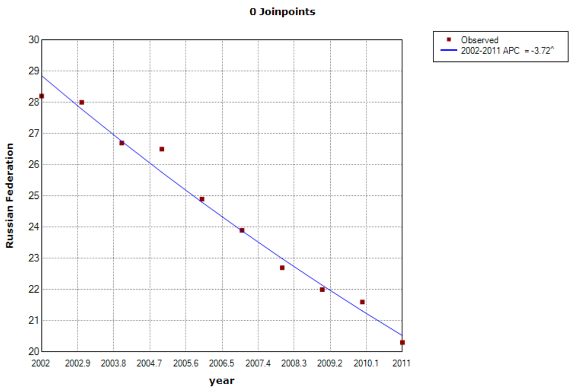

© 2021 Wong MCS et al. *JAMA Network Open.*

h.) Mortality female all ages

Asia

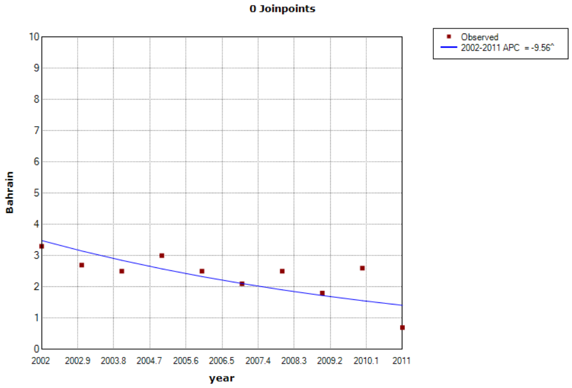

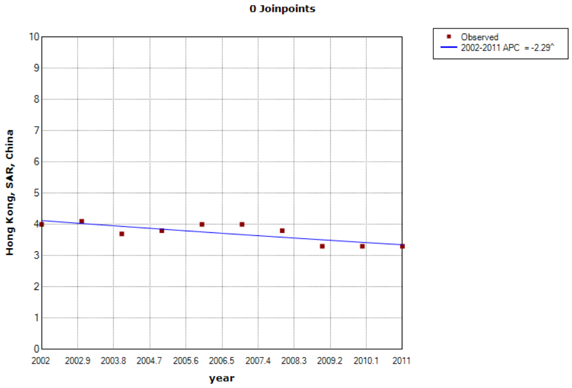

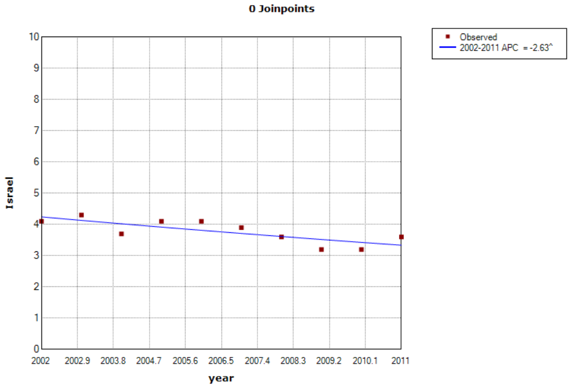

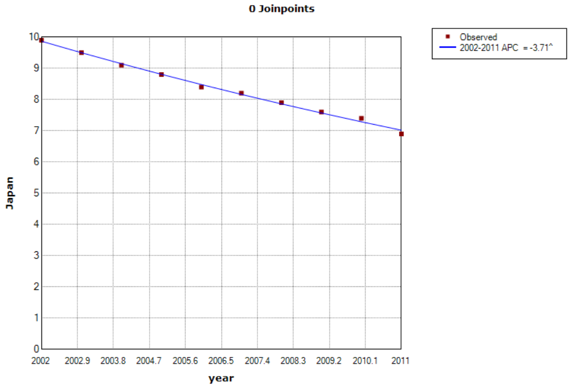

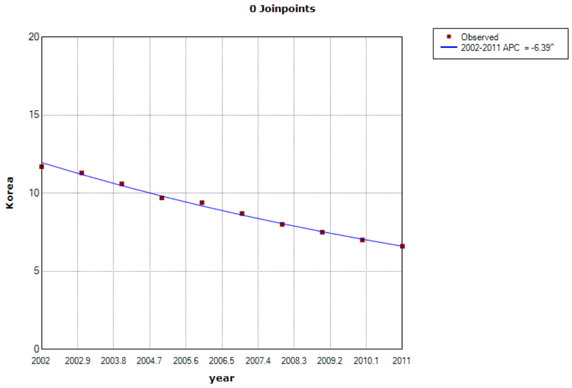

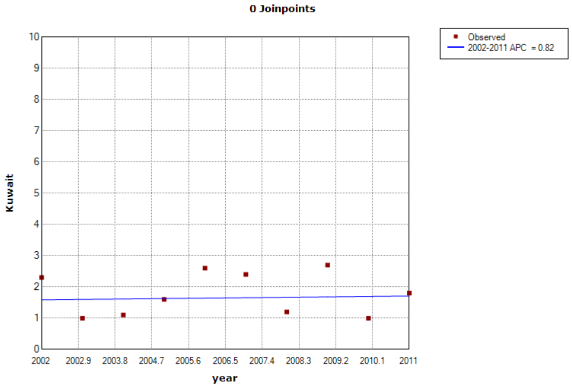

© 2021 Wong MCS et al. *JAMA Network Open.*

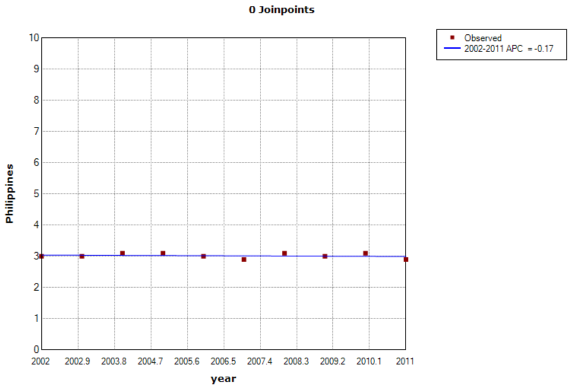

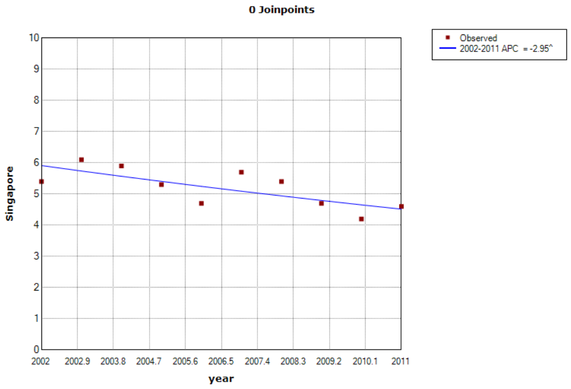

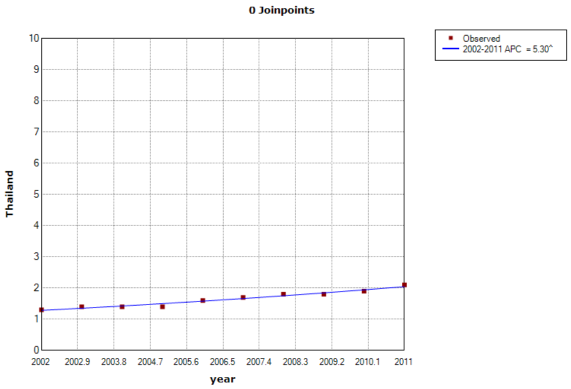

Oceania

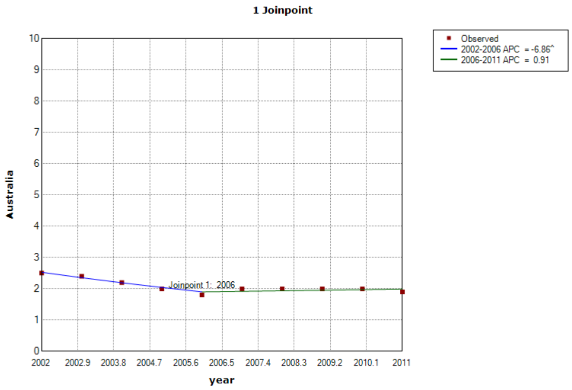

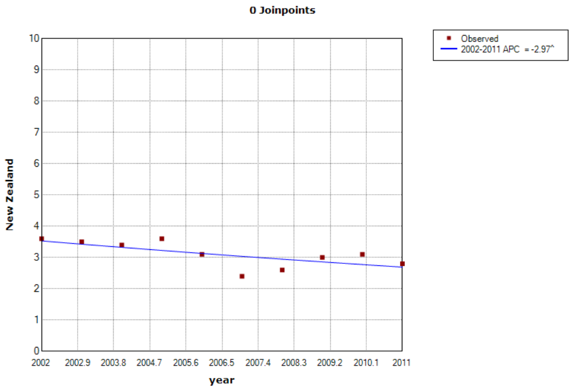

© 2021 Wong MCS et al. *JAMA Network Open.*

Northern America

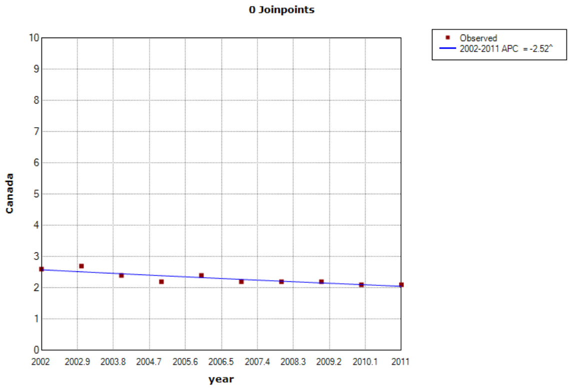

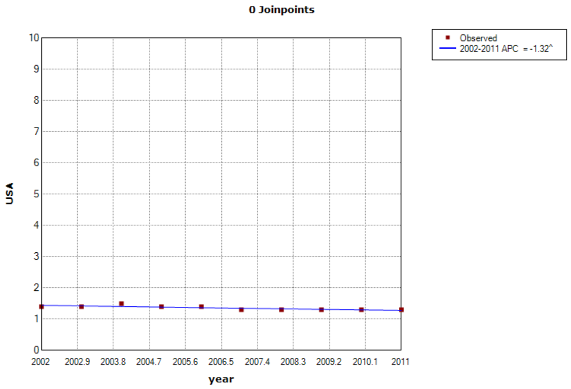

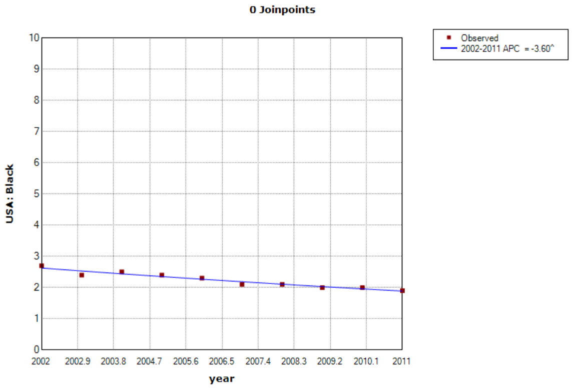

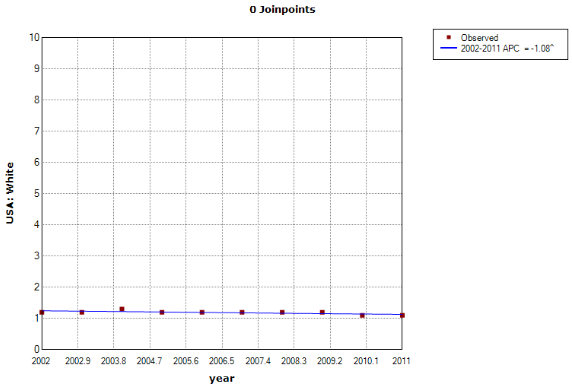

Southern America

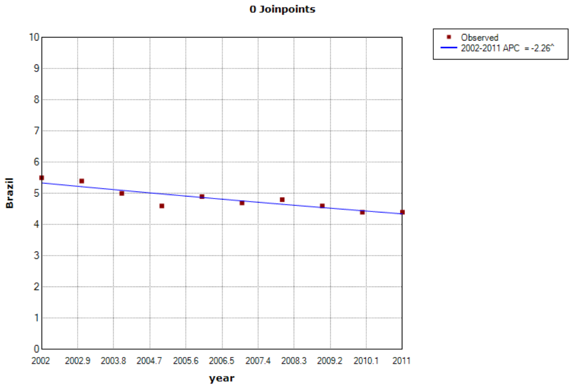

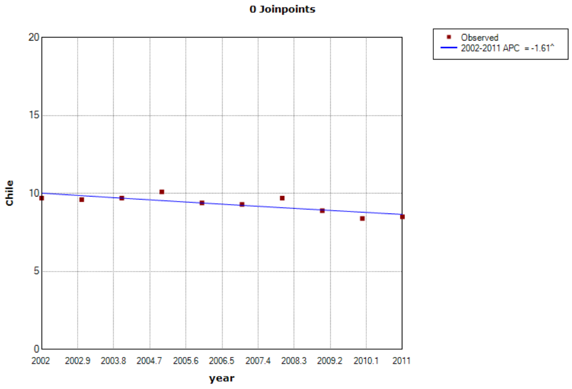

© 2021 Wong MCS et al. *JAMA Network Open.*

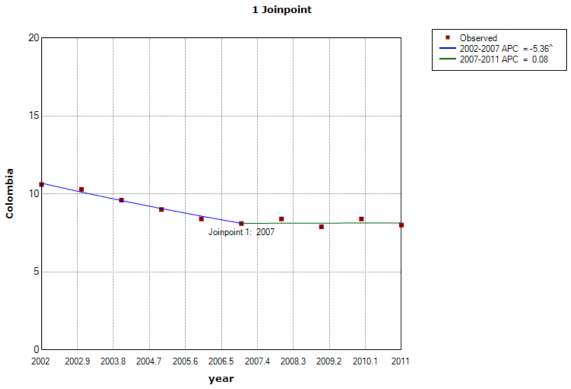

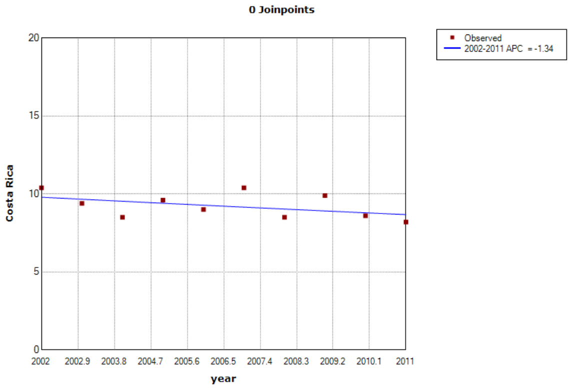

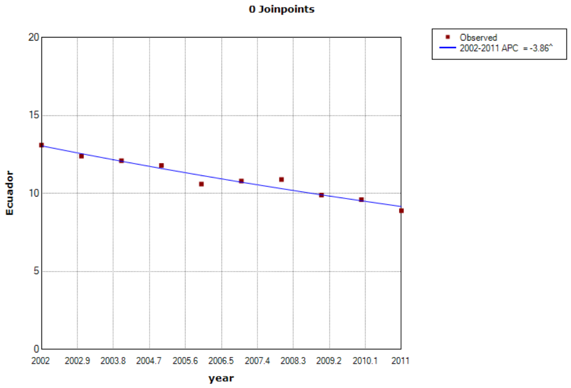

Northern Europe

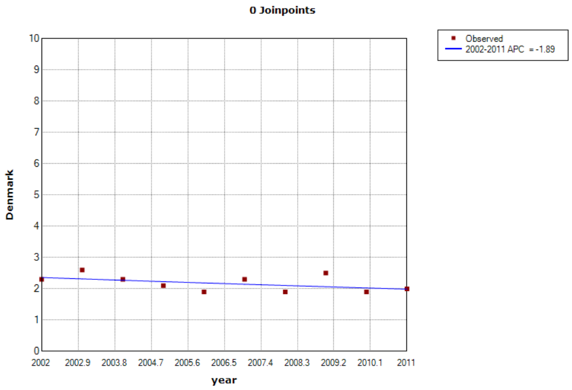

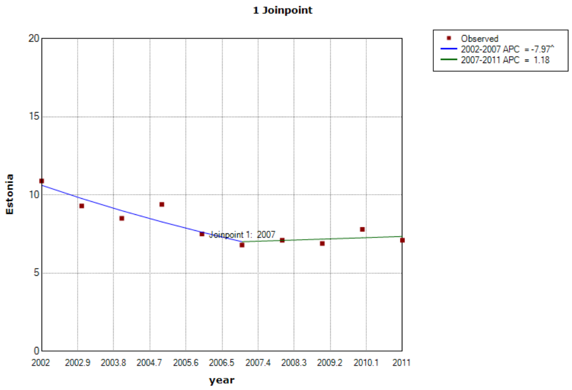

© 2021 Wong MCS et al. *JAMA Network Open.*

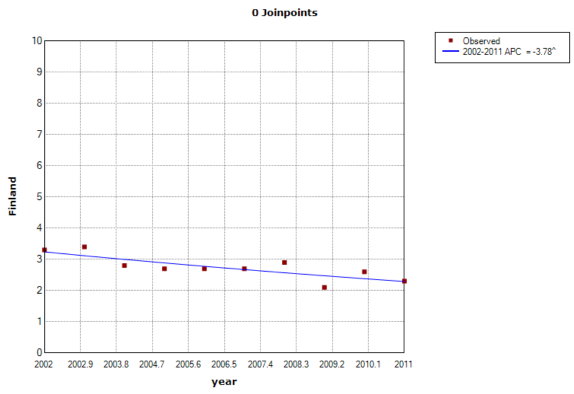

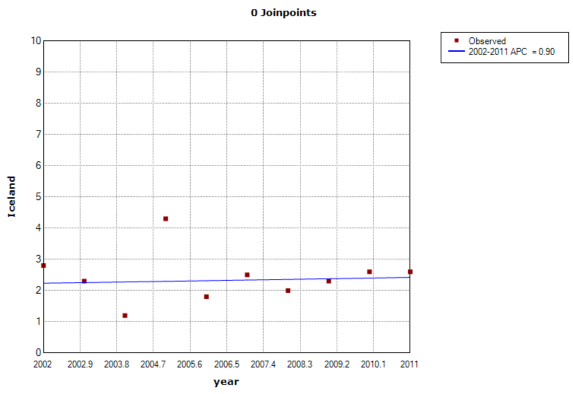

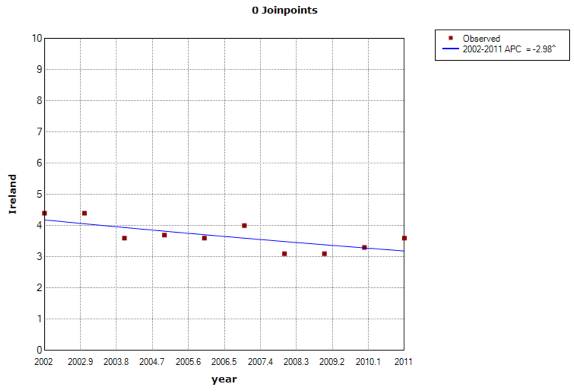

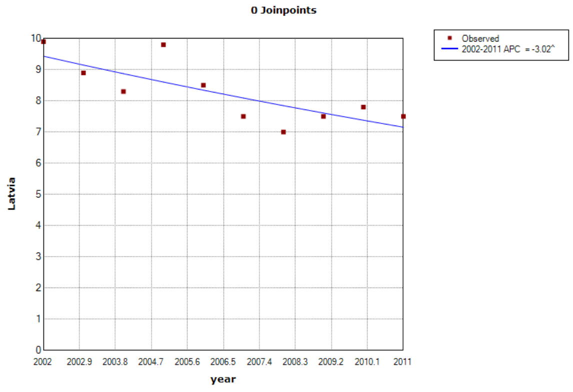

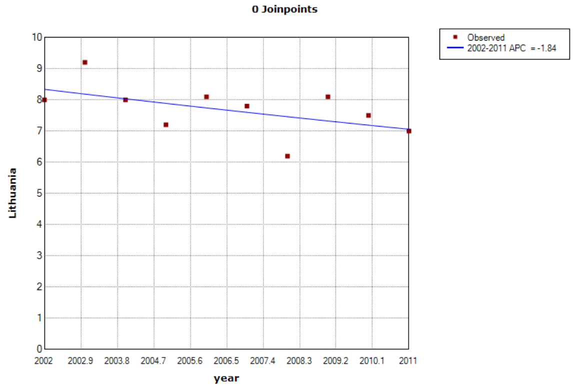

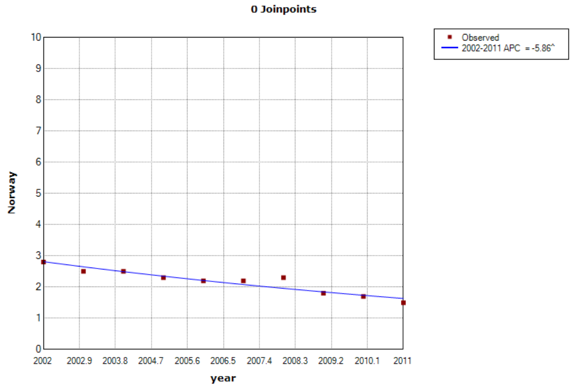

© 2021 Wong MCS et al. *JAMA Network Open.*

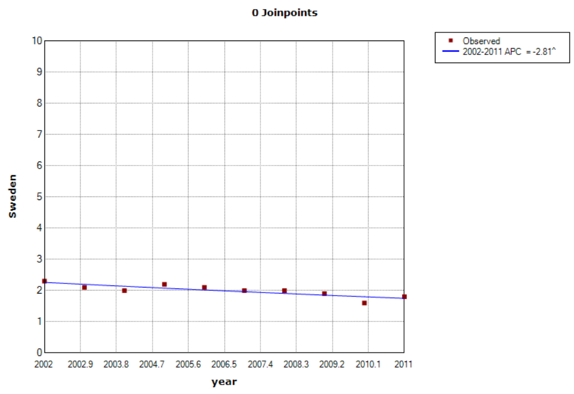

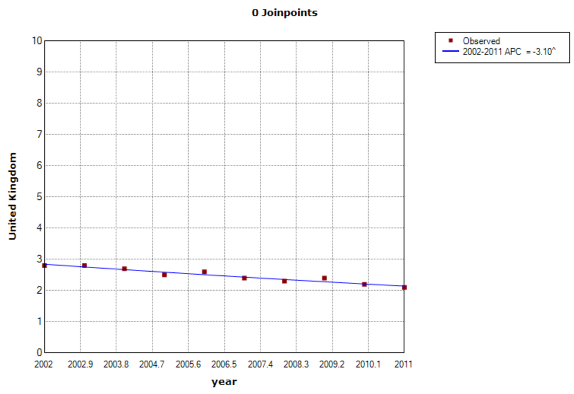

Western Europe

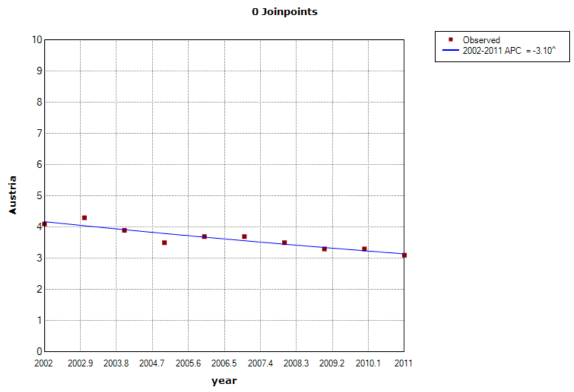

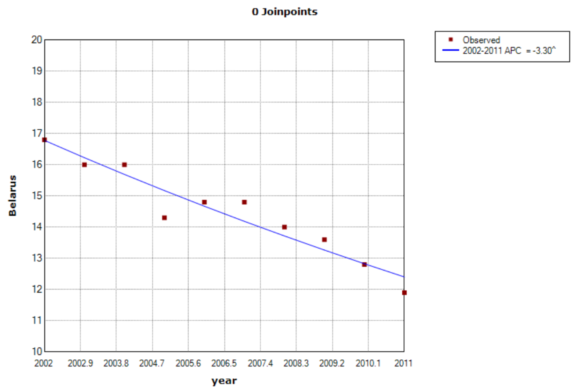

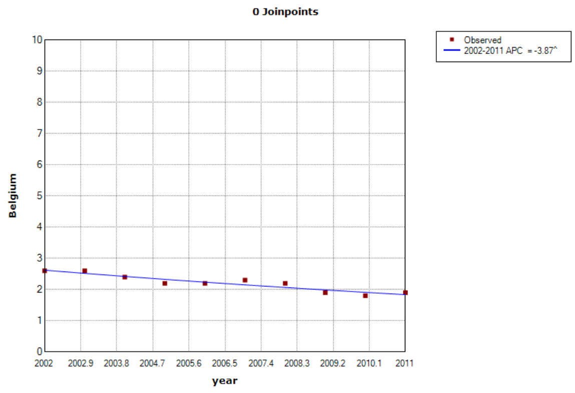

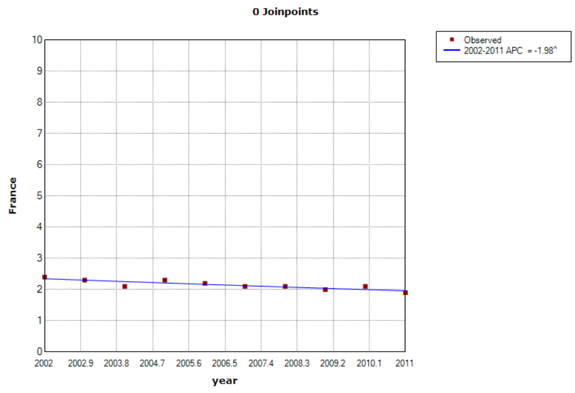

© 2021 Wong MCS et al. *JAMA Network Open.*

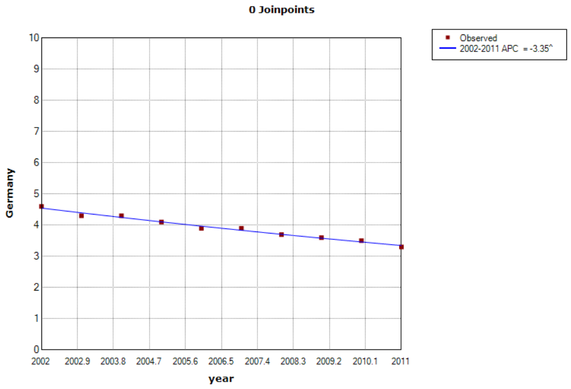

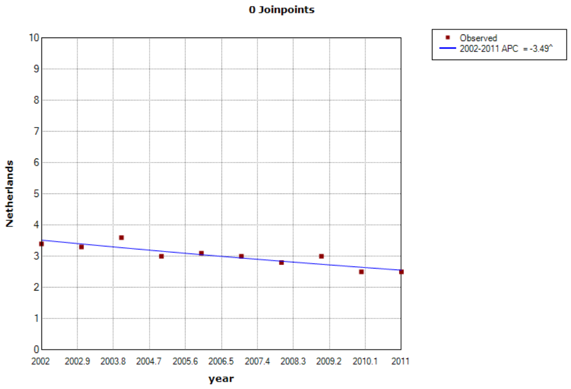

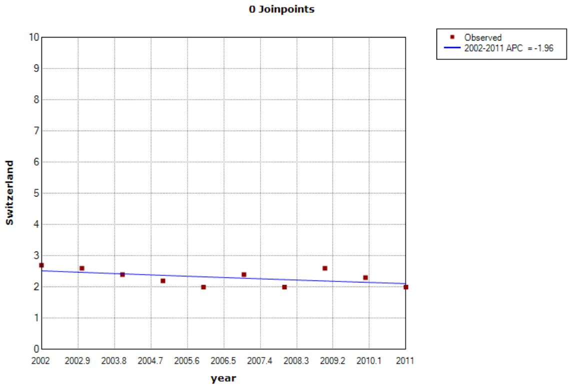

Southern Europe

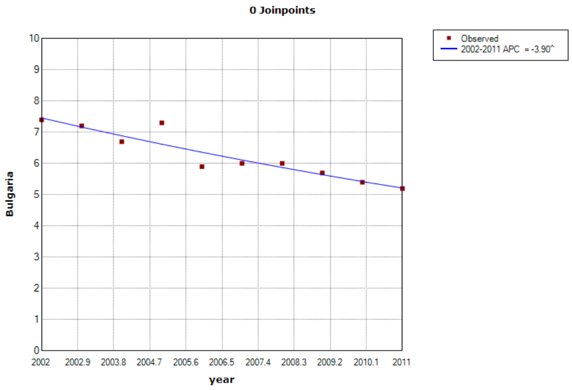

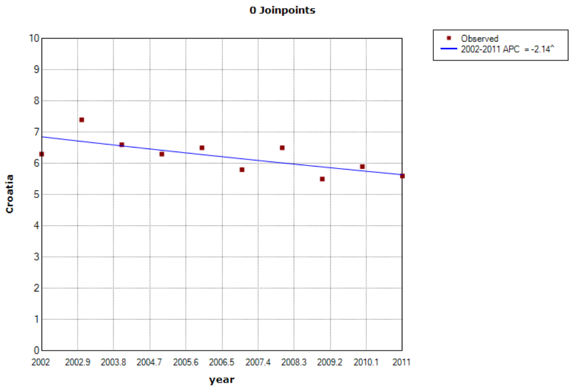

© 2021 Wong MCS et al. *JAMA Network Open.*

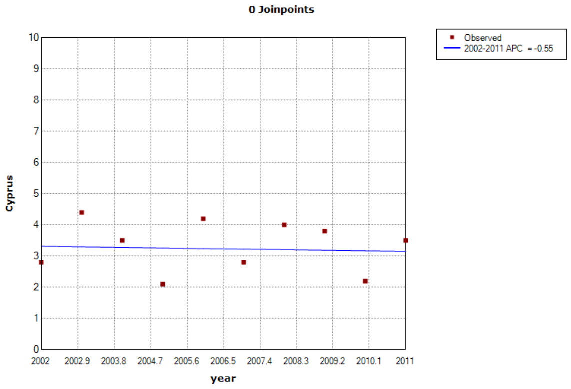

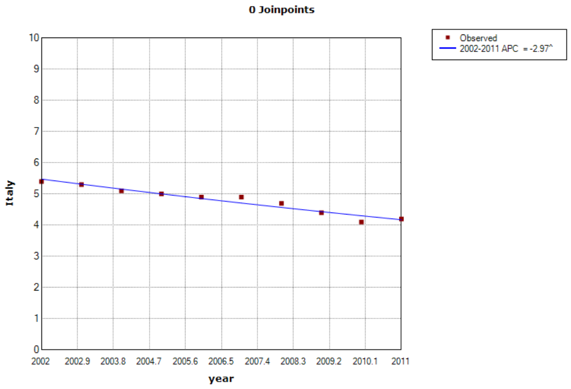

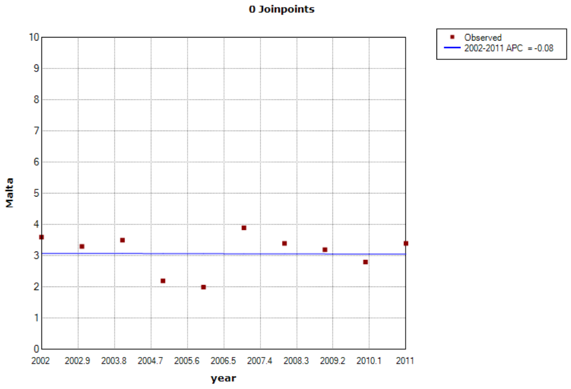

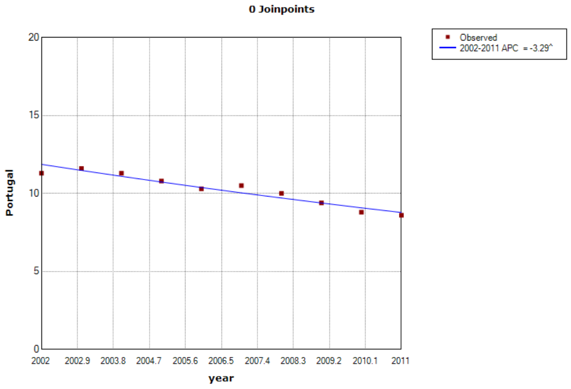

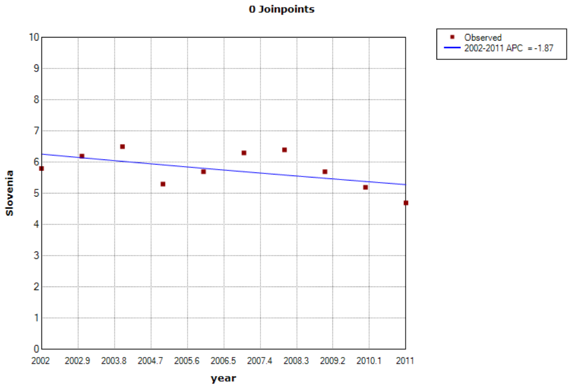

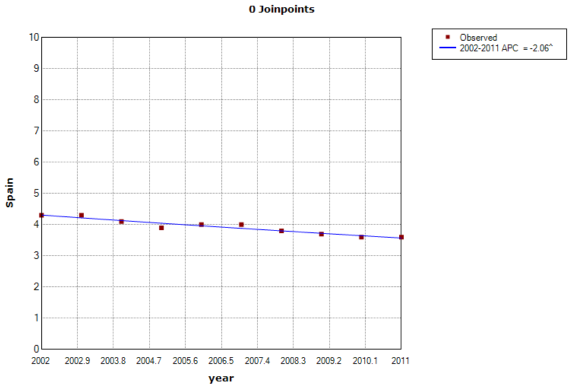

© 2021 Wong MCS et al. *JAMA Network Open.*

Eastern Europe

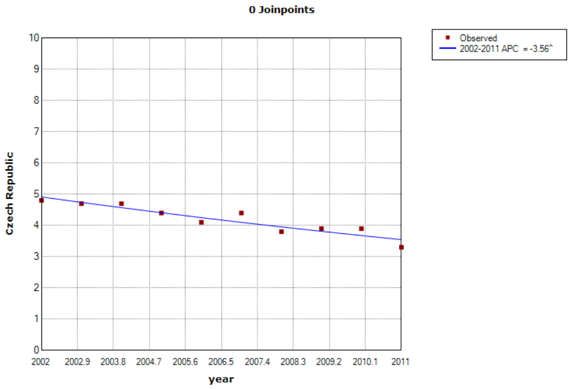

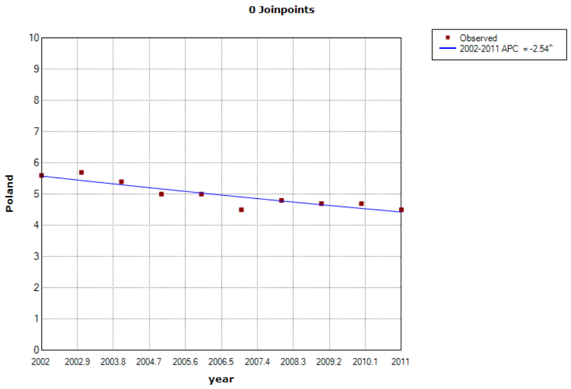

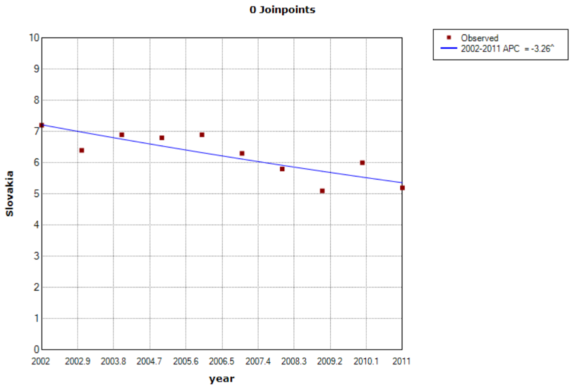

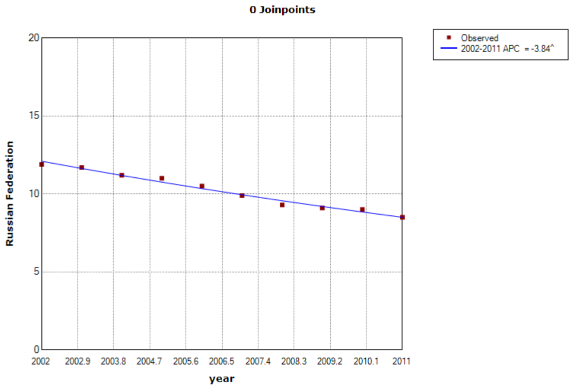

© 2021 Wong MCS et al. *JAMA Network Open.*
